# The PilB-PilZ-FimX regulatory complex of the Type IV pilus from *Xanthomonas citri*

**DOI:** 10.1371/journal.ppat.1009808

**Published:** 2021-08-16

**Authors:** Edgar E. Llontop, William Cenens, Denize C. Favaro, Germán G. Sgro, Roberto K. Salinas, Cristiane R. Guzzo, Chuck S. Farah

**Affiliations:** 1 Departamento de Bioquímica, Instituto de Química, Universidade de São Paulo, São Paulo, Brazil; 2 Departamento de Química Orgânica, Universidade Estadual de Campinas (UNICAMP), Campinas, Brazil; 3 Departamento de Microbiologia, Instituto de Ciências Biomédicas, Universidade de São Paulo, São Paulo, Brazil; Centre National de la Recherche Scientifique, Aix-Marseille Université, FRANCE

## Abstract

Type IV pili (T4P) are thin and flexible filaments found on the surface of a wide range of Gram-negative bacteria that undergo cycles of extension and retraction and participate in a variety of important functions related to lifestyle, defense and pathogenesis. During pilus extensions, the PilB ATPase energizes the polymerization of pilin monomers from the inner membrane. In *Xanthomonas citri*, two cytosolic proteins, PilZ and the c-di-GMP receptor FimX, are involved in the regulation of T4P biogenesis through interactions with PilB. *In vivo* fluorescence microscopy studies show that PilB, PilZ and FimX all colocalize to the leading poles of *X*. *citri* cells during twitching motility and that this colocalization is dependent on the presence of all three proteins. We demonstrate that full-length PilB, PilZ and FimX can interact to form a stable complex as can PilB N-terminal, PilZ and FimX C-terminal fragments. We present the crystal structures of two binary complexes: i) that of the PilB N-terminal domain, encompassing sub-domains ND0 and ND1, bound to PilZ and ii) PilZ bound to the FimX EAL domain within a larger fragment containing both GGDEF and EAL domains. Evaluation of PilZ interactions with PilB and the FimX EAL domain in these and previously published structures, in conjunction with mutagenesis studies and functional assays, allow us to propose an internally consistent model for the PilB-PilZ-FimX complex and its interactions with the PilM-PilN complex in the context of the inner membrane platform of the *X*. *citri* Type IV pilus.

## Author summary

Bacteria have to adapt their lifestyles to changing environments, at times deciding to hunker down and establish compact multicellular colonies with intricate 3-dimensional structures, called biofilms, that provide protection against hostile conditions; while at other times deciding to go out on their own to explore new habitats. Both of these lifestyles rely on Type IV pili, long extendable and retractable surface filaments that allow the bacteria to attach to and move across surfaces. Pilus extension and retraction are powered by two ATPases, PilB and PilT respectively, and there is significant interest in understanding the means by which the activities of these ATPases are controlled. In this study, we explore this theme in the phytopathogen *Xanthomonas citri* that causes citrus canker disease. In *X*. *citri*, the ATPase responsible for pilus extension, PilB, can form a complex with two regulatory proteins, PilZ and the c-di-GMP receptor FimX. We show that the *in vitro* ATPase activity of the PilB-PilZ complex is enhanced by FimX and that these three proteins co-localize at the leading poles of *X*. *citri* cells moving across surfaces. The X-ray structures of PilZ in complex with the N-terminal domain of PilB and with the C-terminal domain of FimX allow us to propose a model for the three-way interaction between these proteins.

## Introduction

Prokaryotes have evolved sophisticated surface nanomachines that allow them to colonize a large variety of niches [1]. One such structure is the type IV pilus (T4P), a flexible filament, 4 to 7 nm in diameter and often several micrometers in length, that can extend, attach to surfaces and retract. T4P are involved in a broad range of functions including twitching motility, adhesion, cell orientation, biofilm formation, pathogenicity, natural transformations and bacteriophage infection [2–7]. The protein machinery required for T4P biogenesis and function is highly conserved among phylogenetically distant bacterial species [8,9] and is related to the ubiquitous type II secretion systems (T2SS) as well as archaeal flagella [10].

T4P filaments are produced by the polymerization of pilin subunits in a process that depends on PilB, a hexameric ATPase associated with the bacterial inner membrane, while pilus retraction is powered by another hexameric ATPase, PilT [11–13]. We have a very rudimentary understanding about these polymerization/depolymerization processes except that, in addition to the above mentioned ATPases, they also require an outer membrane channel formed by the PilQ secretin and an inner membrane platform formed by integral proteins PilC, PilN, PilO, PilP and the cytoplasmic protein PilM [9,14–17]. In contrast to these highly conserved structural components, each of the principle model organisms for which T4P have been extensively studied, such as *Pseudomonas aeruginosa*, *Neisseiria* spp., *Synechocystis* spp., *Vibrio cholerae*, *Myxococcus xanthus* and *Xanthomonas* spp., present unique aspects which point to the evolution of a variety of different molecular mechanisms by which the T4P polymerization and retraction can be controlled [8,18–20]. For example, the function of the PilB homolog MshE from *V*. *cholerae* depends on the binding of Bis-(3′-5′)-cyclic diguanylate (c-di-GMP) to its N-terminal MshEN-N domain [21], while *Xanthomonas* and *Pseudomonas* PilB proteins lack the conserved amino acid residue motifs required for c-di-GMP binding.

In *Pseudomonas aeruginosa* and in several phytopathogenic bacteria of the genus *Xanthomonas*, two regulators of T4P function are PilZ [22,23] and FimX [4,24–27]. *Pseudomonas* and *Xanthomonas* PilZ are small proteins that do not bind c-di-GMP in spite of belonging to the PilZ superfamily of proteins, many known to be c-di-GMP receptors [23,27,28]. FimX, on the other hand, is a large protein with four domains: REC, PAS, GGDEF and EAL, three of which can be considered degenerate in relation to their canonical functions. The FimX REC domain lacks a site for phosphorylation by a cognate histidine kinase, the GGDEF domain does not possess diguanylate cyclase activity and the EAL domain does not possess phosphodiesterase activity, although it does retain the ability to bind c-di-GMP [24,25,29]. In *X*. *citri*, PilZ has been shown to interact with both FimX and PilB and knockouts of these proteins abolish T4P-dependent functions in *X*. *citri* [4,23,24] and *P*. *aeruginosa* [22,25,26]. PilZ-FimX interactions have been observed for the proteins from *X*. *campestris* pv. *campestris* [30] and *X*. *oryzae* pv. *oryzae* [31] and PilB-PilZ interactions have been observed in the closely related Xanthomonadaceae species *Lysobacter enzymogenes* [32]. Interestingly, in the more distantly related *P*. *aeruginosa*, experiments failed to detect interactions between PilZ and FimX [33]; instead direct interactions between FimX and PilB have been observed [34].

To better understand the three-way interactions between PilB, PilZ and FimX, we determined the crystal structure of the complex between PilZ and a PilB fragment (PilB_12-163_) corresponding to the N-terminal domain of PilB from the phytopathogen *Xanthomonas citri* that causes canker disease in citrus plants. PilB_12-163_ is made up of two sub-domains, ND0 and ND1, and is structurally similar to the N-terminal domains of *V*. *cholera* MshE (MshEN, residues 1–145) [21] and *X*. *campestris* XpsE (XpsE_Nt_, residues 1–149) [35]. NMR experiments show that PilB_12-163_ can undergo a large structural rearrangement upon interaction with PilZ. We demonstrate that full-length PilB, PilZ and FimX can interact to form a stable complex, as can PilB_12-163_, PilZ and C-terminal FimX fragments FimX_EAL_ or FimX_GGDEF-EAL_. We also crystallized the complex formed by PilZ_Δ107–117_ and FimX_GGDEF-EAL_. Evaluation of PilZ interactions with PilB_12-163_ and the FimX EAL domain via functional assays of specific mutants allow us to propose a consistent model for PilB-PilZ-FimX ternary complex and its interactions with other T4P components in the inner membrane. This is the first atomic resolution model of a PilB protein in complex with a specific protein regulator and so provides us with unique structural insights into the means by which the activity of a T4P polymerization ATPase can be controlled.

## Results

### PilZ interacts with the N-terminal domain of PilB

**Fig 1:**
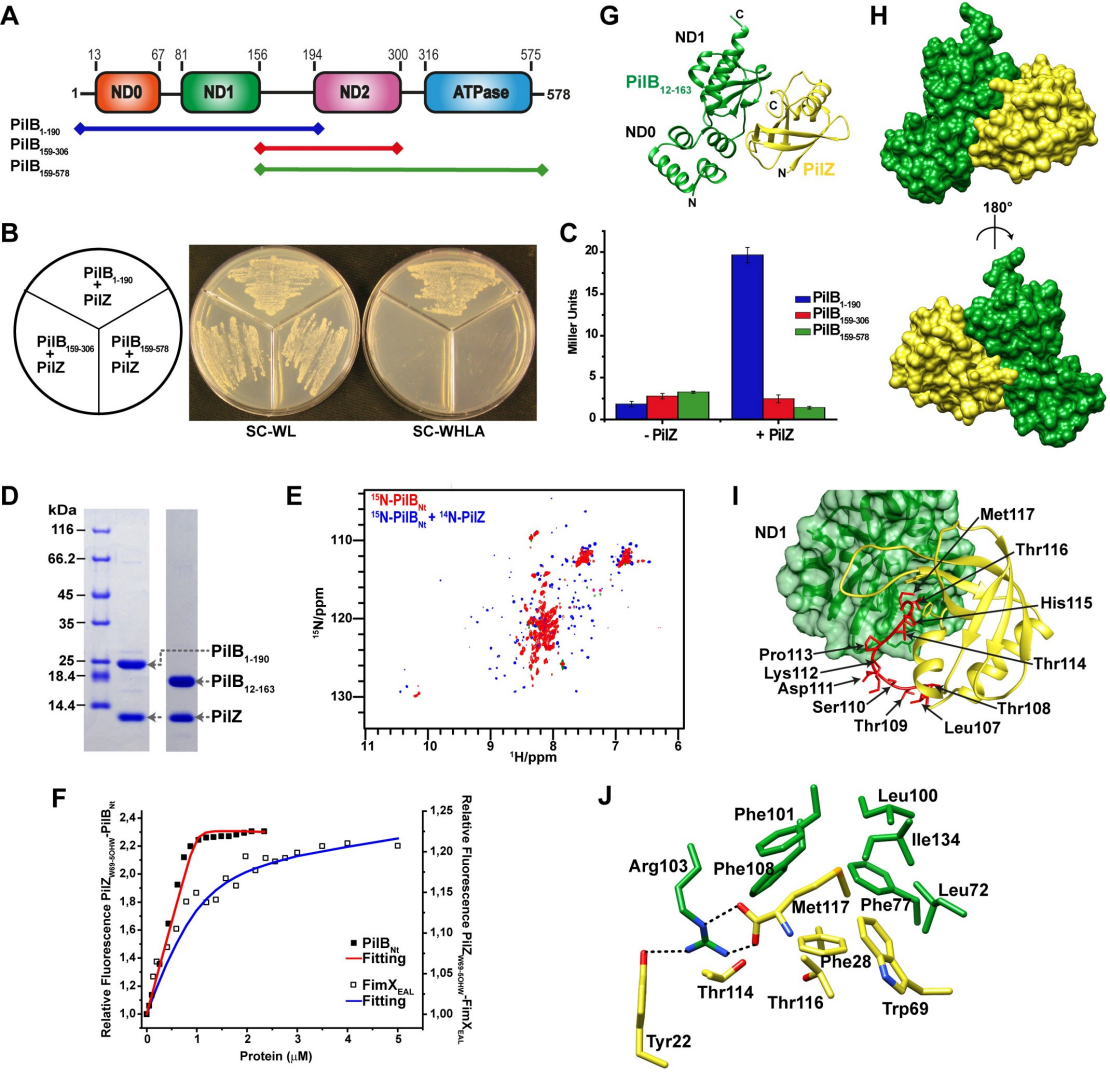
PilZ interacts with the N-terminal ND0/ND1 domain of PilB. **A)** Schematic model of *X*. *citri* PilB domains. Fragments used as preys in the two-hybrid assays are indicated with coloured lines: PilB_1-190_ (blue), PilB_159-306_ (red), PilB_159-578_ (green). **B)** Two hybrid assay showing yeasts cells grown on non-selective (SC-WL) and selective (SC-WHLA) media. Three different fragments of PilB were used as shown in A, only cells containing the PilZ-PilB_1-190_ bait-prey combinations are able to grow on SC-WHLA media. **C)** Two-hybrid assay showing β-galactosidase activity of yeast cells containing only the PilB preys (left) and the PilZ-PilB bait-prey combinations. Only cells containing the PilZ-PilB_1-190_ bait-prey combination exhibit significant β-galactosidase activity. **D)** Co-expressions and co-purification of PilB_1-190_-PilZ- and PilB_12-163_-PilZ complexes from *E*. *coli* cells. Complexes were first purified on a Ni^2+^-NTA column due to an N-terminal His-tag on PilB, the His tag removed by proteolytic cleavage, and the complex was further purified by passage through a Superdex 200 10/300 SEC column. **E)** ^1^H-^15^N HSQC spectrum of ^15^N-labeled PilB_12-163_ (lacking the 6xHis-tag) acquired at 25°C (red) shows that it has a partially disordered structure and that addition of unlabeled PilZ leads to folding of PilB_12-163_ (blue). **F)** The fluorescence of PilZ_W69_5OHW_ was monitored during its titration with His-tagged PilB_1-190_ (black boxes) or FimX_EAL_ (white boxes). 1 μM of PilZ_W69_5OHW_ was used. Each point is the average of at least two measurements. The fits (continuous lines) and the dissociation constants were calculated as previously described [82]. **G)** Ribbon representation of the PilB_12-163_-PilZ complex with PilB colored green and PilZ colored yellow. The ND0 and ND1 sub-domains of PilB are indicated. **H)** Surface representation of the complex. **I)** Residues at the interaction interface between PilB (*left*) and PilZ (*right*). The PilZ MV motif makes important and extensive contacts with the ND1 sub-domain of PilB. MV motif residues (red) are indicated. **J)** Residue Met117 of PilZ fits into a hydrophobic pocket lined by conserved hydrophobic residues of PilZ and PilB.

We have previously shown that PilZ interacts with the hexameric ATPase PilB, required for Type IV pilus biogenesis [23]. **Fig 1A** shows that the 578 residue PilB protein from *X*. *citri* can be divided into 3 domains: an N-terminal domain made up of sub-domains ND0 and ND1, a central ND2 domain and a C-terminal ATPase domain [36]. In order to determine which PilB domains are involved in the interaction with PilZ, we produced three preys consisting of fragments spanning sub domains ND0-ND1 (residues 1–190; PilB_1-190_), domain ND2 (residues 159–306, PilB_159-306_) and domains ND2-ATPase (residues 159–578, PilB_159-578_) for use in yeast two-hybrid interaction assays with a PilZ bait. **Fig 1B and 1C** shows that an interaction was only detected using the PilB_1-190_ prey. This result allows us to conclude that the PilB-PilZ interaction is mediated via the former´s N-terminal region.

Based on these results, we expressed and purified PilB_1-190_ and a smaller PilB N-terminal fragment corresponding to residues 12–163 (PilB_12-163_) that lacks regions predicted to be unstructured at the N- and C-terminal ends of PilB_1-190_. **Fig 1D** shows that PilB_1-190_-PilZ and PilB_12-163_-PilZ complexes can be co-purified after co-expression in *E*. *coli* cells (in these experiments the PilB fragments were expressed with a cleavable N-terminal 6xHis tag). ^15^N-labelled PilB_12-163_ has an ^1^H-^15^N HSQC NMR spectrum characteristic of a partially disordered polypeptide. However, in the presence of unlabelled PilZ, the ^1^H chemical shift dispersion increases which is indicative of a folded protein (**Fig 1E**). **S1 Fig** shows the results of typical SEC-MALS (size-exclusion chromatography coupled with multi-angle light scattering) experiments that show that PilB_1-190_ and PilB_12-163_ are predominantly monomeric on their own and each can form 1:1 binary complexes with PilZ.

In order to compare the affinities of PilZ for PilB and FimX, we expressed PilZ in a tryptophan auxotrophic strain of *E*. *coli* grown in minimal media containing 5-hydroxytryptophan (5OHW). The recombinant PilZ protein, PilZ_W69_5OHW_, now contains this modified amino acid at position 69, normally occupied by tryptophan. The titration of PilZ_W69_5OHW_ by PilB_1-190_ was accompanied by a 2.5-fold increase in 5OHW fluorescence and the data indicate that the dissociation constant is in the nanomolar or subnanomolar range (**Fig 1F**). The calculated *K*_D_ of 4.1 ± 0.9 nM should be taken as an upper limit since the PilZ protein concentration used in the experiment (1 μM) is between two and three orders of magnitude greater than the calculated dissociation constant. PilZ_W69_5OHW_ was also titrated by a fragment corresponding to the C-terminal EAL domain of FimX (FimX_EAL_) [24], in which case a more moderate 25% increase in fluorescence was observed and the dissociation constant was calculated to be 0.2 μM ± 0.1 μM (**Fig 1F**). These results indicate that the PilZ interaction with PilB_1-190_ is at least one or two orders of magnitude stronger than its interaction with FimX_EAL_. We note that these experiments were performed in the absence of added c-di-GMP and that the W69_5OHW substitution may be affecting PilZ´s interactions with PilB and FimX to different degrees (see below).

### The structure and interface of the PilB_12-163_-PilZ complex

Native and selenomethionine PilB_12-163_-PilZ complexes were crystallized as described in Materials and Methods, in space group P2_1_. Two native crystal datasets were obtained, one with the wild type sequences and another with a spontaneous P70S mutation in the PilB_12-163_ subunit. Data collection statistics for all three crystals are presented in **S1 Table**. Initial phases were estimated by single wavelength anomalous dispersion. The asymmetric unit contains two copies of the PilB_12-163_-PilZ complex (chains AC and chains BD) (**S2A Fig**) and the overall structure of the heterodimeric complex is shown in **Fig 1G and 1H**. The PilB_12-163_-PilZ interface buries 1026 Å^2^ and 1018 Å^2^ of surface area for the AC and BD PilB_12-163_-PilZ dimers, respectively, as calculated by PISA [37]. A list of residues found at the interface is presented in **S2 Table**. Structural alignment of the two PilB_12-163_ fragments (chains A and B) and the two PilZ molecules (chains C and D) show that they are very similar with a root mean square deviation (RMSD) of 0.6 Å and 0.4 Å for C^α^ atoms, respectively. No electron density was observed for the last 6 residues of PilB_12-163_ and the first 8 residues of PilZ.

PilB_12-163_ is made up of two sub-domains, a four helix ND0 (α1-α4) and an α/β ND1 (a β1-β3 antiparallel β-sheet surrounded by three helices (α5-α7) and a string of three consecutive short 3_10_ helices (η1-η3)) (**Figs 1G and S2B**). The ND0 and ND1 sub-domains are topologically similar to the MshEN_N and MshEN_C domains, respectively, of the *V*. *cholerae* T4P ATPase MshE. It is noteworthy that *X*. *citri* PilB sub-domain ND0 lacks the specific amino acid sequence motifs required for c-di-GMP binding found in the MshEN_N domain.

**Fig 2:**
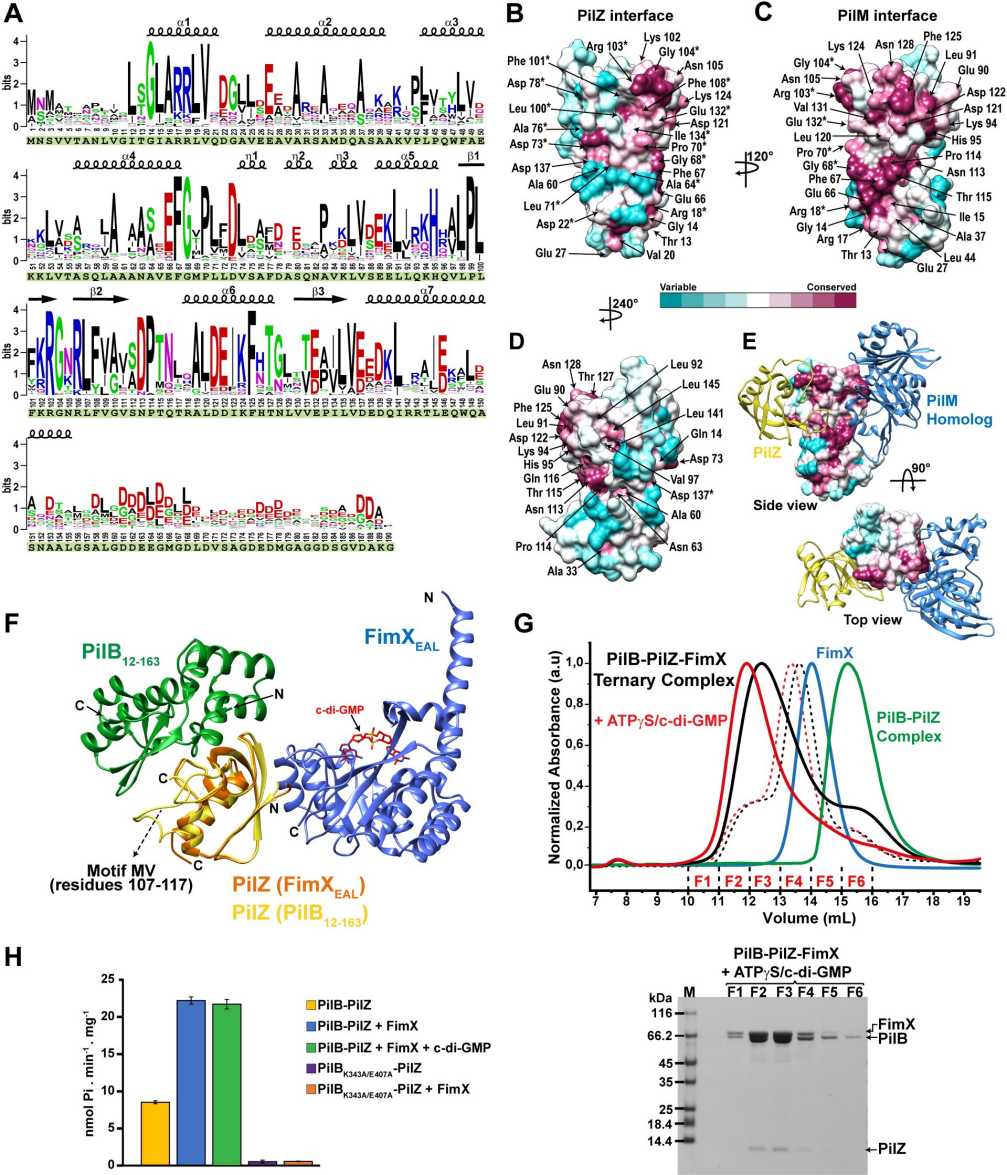
PilB sequence conservation and interactions. **A)** Sequence logo generated from an alignment of 50 non-redundant PilB N-terminal sequences that contain ND0 and ND1 sub-domains. The *X*. *citri* PilB N-terminal (residues 1–190) sequence is shown below the alignment (highlighted in green). The secondary structure elements observed in the crystal structure of *X*. *citri* PilB (residues 12–163) are indicated above the alignment. **B-D)** Surface residues of PilB_12-163_ colored according to degree of conservation. Panel **B** shows the PilB_12-163_ surface oriented towards PilZ in the PilB_12-163_-PilZ complex. * Indicates residues involved in direct contacts with PilZ. Panels **C** and **D** show PilB_12-163_ after 120^o^ and 240^o^ rotations with respect to **A.** The surface in **C** presents residues proposed to be involved in the interaction with PilM. **E)** Possible mode of interaction between PilB_,_ PilZ and PilM. Shown is the superposition of the *X*. *citri* PilB_12-163_-PilZ complex with homologous EpsE_ND1_-EpsL complex (PDB ID: 2BH1) using the ND1 sub-domains as reference (see **S12 and S13 Figs**). PilZ and the EpsL cytoplasmic domain (PilM homolog) are shown in ribbon representation colored in yellow and blue, respectively. **F)** Proposed model for the PilB-PilZ-FimX ternary complex based on the superposition of the PilB_12-163_-PilZ complex and the PilZ-FimX_EAL_ complex (interface 2). PilZ is the reference in the superposition. **G)** *Above*: SEC analysis of the mixture of full-length PilB-PilZ complex and full-length FimX shows that these proteins form a ternary complex (black continuous line). Addition of ATPγS and c-di-GMP results in a shift in the elution profile of the ternary complex (red continuous line). Also shown is the elution profile of the PilB_K343A/E407A_-PilZ and FimX mixture in absence (dashed black line) and presence (dashed red line) of ATPγS and cdi-GMP. In all cases, a 1:1 molar ratio of PilB-PilZ complex to FimX was used. The elution profiles of full-length FimX (blue continuous line) and the PilB-PilZ complex (green continuous line) are also shown. To facilitate comparison, peak heights were normalized. *Below*: SDS-PAGE of the fractions of the major peak observed for the PilB-PilZ-FimX ternary complex in presence of ATPγS and cdi-GMP. **H)** ATPase activity of the PilB-PilZ complex in the absence and presence of full-length FimX. ATPase activity was determined by measuring the production of inorganic phosphate (Pi) at 30°C using 1 μM of PilB-PilZ complex, 1 mM de ATP in the absence and presence of 1 μM FimX. Where indicated, c-di-GMP was added to a final concentration of 20 μM. When wild-type PilB was substituted with the PilB_K343A/E407A_ mutant, no significant ATPase activity was observed. Each experiment was performed at least three times. Error bars: standard deviation.

Bioinformatics analysis indicates highly conserved PilB residues at the PilB-PilZ interface. **S3 Table** lists 51 bacterial species from 51 different genera whose genomes, all annotated in the KEGG database [38], code for all three PilB, PilZ and FimX homologs as well as a homolog of the conserved T4P component PilM. All except one of these genera are from the class Gammaproteobacteria but are distributed among different orders and families. The coincidence of genes coding for close PilB, PilZ and FimX homologs in different genomes could reflect a shared and conserved mechanism of T4P regulation in these species. We note that all of the PilB sequences in this list present both ND0 and ND1 sub-domains and none of the PilB sequences carry residues implicated in the binding of c-di-GMP by MshE and its closest homologs. **Fig 2A** presents the conservation profile of the N-terminal domains of these PilB proteins using the numbered positions of *X*. *citri* PilB as a reference. This conservation profile is mapped onto the PilB_12-163_ structure in **Fig 2B–2D**. **Fig 2B** presents the PilB_12-163_ surface involved in interactions with PilZ (predominantly through the former´s ND1 sub-domain; see also **Fig 1G, 1H** and **S2 Table)**. **Fig 2C and 2D** presents the exposed PilB_12-163_ surfaces after rotations of 120^o^ and 240^o^ about the vertical axis, respectively. Interestingly, in addition to the conservation of residues interacting with PilZ, the PilB_12-163_ surface viewed by the 120^o^ rotation is also very well-conserved while the surface viewed by the 240^o^ rotation is relatively poorly conserved. Below, we will show that this second conserved PilB surface is most likely involved in interactions with PilM (**Fig 2E**).

The superposition of PilZ of the PilB_12-163_-PilZ complex with the previously described crystallographic structures of *X*. *citri* PilZ [23] and the PilZ-FimX_EAL_ complex [24] shows that they are very similar, with a RMSD of 0.7 Å and 0.5 Å for C^α^ atoms, respectively (**S2D Fig**). The most significant difference between these three PilZ structures is that the electron density for the last 11 PilZ residues (residues 107–117) is well-defined in the PilB_12-163_-PilZ complex (**Figs 1I and S2E and S2F**), while no density is observed for these residues for PilZ alone [23] and in the FimX_EAL_-PilZ complex [24] (**S2D Fig**). These C-terminal residues (called motif V, or MV) are highly conserved in *X*. *citri* PilZ orthologs, including PilZ_PA2960_ from *P*. *aeruginosa*, and are required for the interaction with PilB but not with FimX_EAL_ [23]. Specifically, in the PilB_12-163_-PilZ complex, the carboxylate group of the C-terminal Met117 residue of PilZ makes a salt bridge with highly conserved Arg103 of PilB while the Met117 side chain fits into a hydrophobic pocket made up of PilB residues Leu72, Phe77, Leu100, Phe101, Phe108 and Ile134 and conserved PilZ residues Phe28 and Trp69 (**Fig 1I and 1J and S2 Table**). In addition to motif V, many other PilZ residues conserved in the PA2960/XAC1133 orthologous group [23] also participate in interactions with PilB (**S2 Table**).

### Mutations at the PilB-PilZ interface destabilize the complex

We produced mutants in specific residues at the PilB-PilZ interface and tested the stability of these complexes using size-exclusion chromatography (SEC). **S3A–S3F Fig** shows the results using PilZ mutants in complex with PilB_1-190_ (this larger PilB fragment was used due to its greater solubility in the absence of PilZ). Mutations in conserved PilZ residues Y22 and F28 did not affect binding to PilB_1-190_ (**S3A and S3B Fig**). On the other hand, mutating PilZ residue W69 to alanine severely reduced the stability of the complex (**S3C Fig**). The PilZ_Δ107–117_ mutant also failed to make a complex with PilB_1-190_ (**S3E Fig**), consistent with previous observations [23]. Since the C-terminal residue M117 makes a number of contacts in the interface (**Fig 1J**), we produced two more PilZ mutants, one in which M117 was deleted (eliminating both carboxy-terminal and side chain interactions) and another in which it was substituted for a glycine residue (eliminating side-chain interactions). In both cases, interaction with PilB_1-190_ was abolished (**S3D and S3F Fig**). We also mutated PilB residues found at the PilB-PilZ interface: F77A, F101A, F108A, R103A and E132A and the double mutant F101A/F108A. **S3G–S3K Fig** shows that all of the single mutants in PilB_1-190_ retain the ability to interact with PilZ. However, the F101A/F108A double mutant no longer interacts (**S3L Fig**). Together these mutation studies point to the importance of hydrophobic interactions in the stabilization of the PilB-PilZ interface.

### Determination of the relevant PilZ-FimX interface in the PilB-PilZ-FimX complex: PilZ interacts with FimX through a second interface distinct from that between PilZ and PilB

We also crystallized the complex between PilZ_Δ107–117_ and a larger FimX fragment encompassing its GGDEF and EAL domains (FimX_GGDEF-EAL_, residues 255–689) in the presence of c-di-GMP (**S4 Fig**). The PilZ_Δ107-117_-FimX_GGDEF-EAL_ complex crystallized in space group P4_2_2_1_2 with one copy of each protein in the asymmetric unit (see **S1 Table for** data collection statistics**)**. Very clear electron density for PilZ_Δ107–117_ and the FimX EAL domain (beginning at residue 436) with bound c-di-GMP in the *syn/anti* conformation were observed (**S4C and S4D Fig**). However, no density for the FimX GGDEF domain could be detected (**S4D and S4E Fig**), a phenomenon similar to that observed for the crystal structure of the FimX_GGDEF-EAL_ fragment from *P*. *aeruginosa* [29] that is only 30% identical to *X*. *citri* FimX_GGDEF-EAL_.

**Fig 3:**
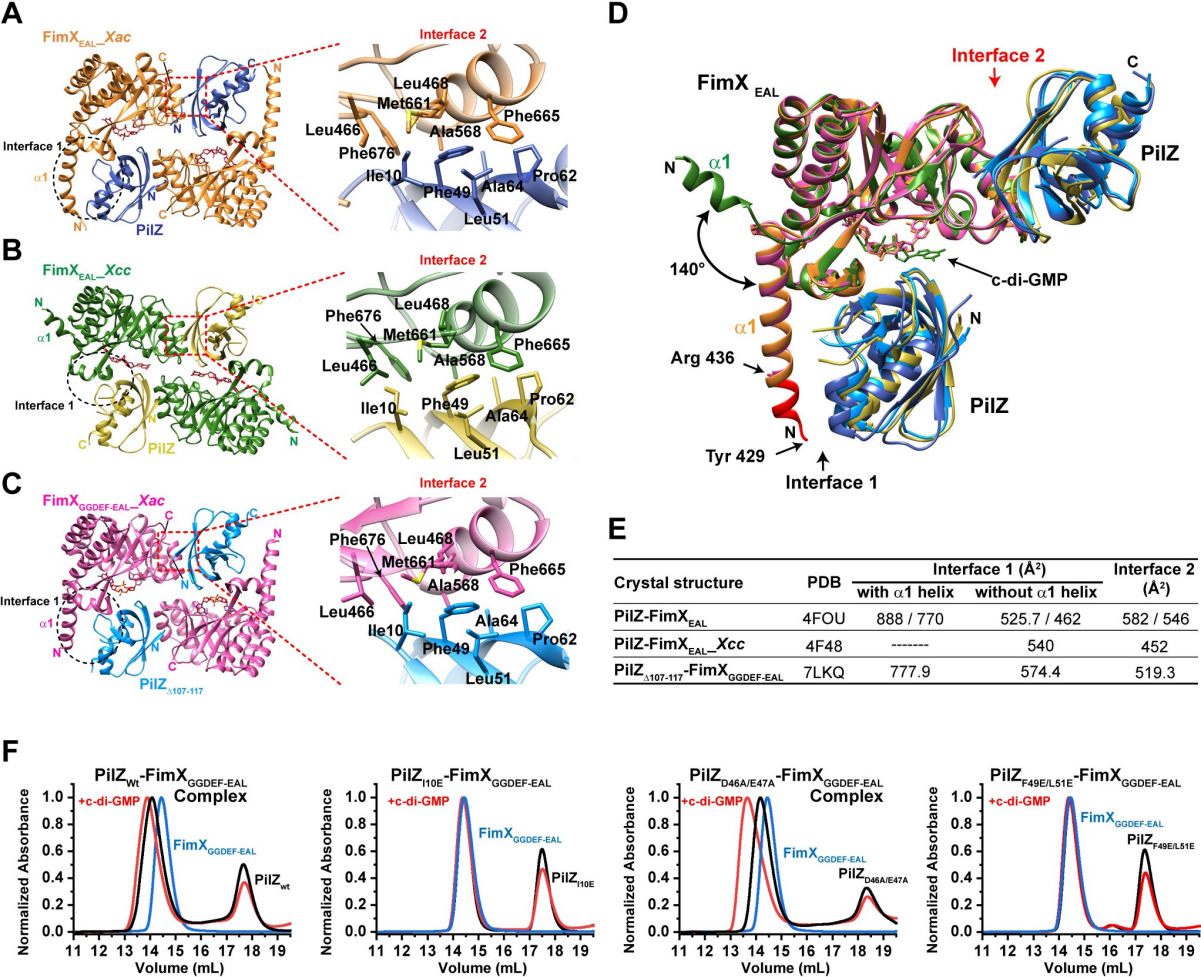
Alternative modes of contact between PilZ and the FimX EAL domain observed in different crystal structures. **A-C)***Left*: The principal crystal contacts observed between PilZ and FimX subunits in the **A**) PilZ-FimX_EAL_-c-di-GMP complex from *X*. *citri* (PDB: 4FOU; FimX_EAL_ colored in orange and PilZ colored in blue), **B**) PilZ-FimX_EAL_-c-di-GMP complex from *X*. *campestris* pv. *campestris* (PDB: 4F48; FimX_EAL_ colored in green and PilZ colored in yellow) and **C**) PilZ_Δ107-117_-FimX_GGDEF-EAL_-c-di-GMP complex from *X*. *citri* (this study; FimX_EAL_ colored in magenta and PilZ colored in cyan). The dashed black circles and dashed red boxes delimit the two principal crystal contacts (interface 1 and 2, respectively). *Right*: Detailed view of interface 2 in the three structures. **D**) Superposition of one FimX_EAL_ domain with the two contacting PilZ subunits via interfaces 1 and 2 observed in the three structures shown in A-C with the same coloring scheme. Note the different orientations of the first alpha helix in the *X*. *citri* and *X*. *campestris* pv. *campestris* FimX EAL domains. This explains the reduced contact surface at interface 1 in the latter structure (see part **E**). **E**) Buried surface areas at the two interfaces in the three structures as calculated by the PISA server [37]. **F**) Size exclusion chromatography of PilZ-FimX_GGDEF-EAL_ binary complexes containing wild-type PilZ (PilZ_Wt_) and its mutants (PilZ_F49E/L51E_, PilZ_I10E_ and PilZ_D46A/E47A_). In each chromatogram, the elution profile of the FimX_GGDEF-EAL_−PilZ mixture (1:1.5 molar ratio) was performed in the absence (black line) and in the presence (red line) of a 2-fold excess of c-di-GMP to FimX_GGDEF-EAL_. In these experiments, FimX_GGDEF-EAL_ has an N-terminal 6xHis-tag. Each experiment was performed at least three times and representative results are shown. These chromatograms are reproduced in **S5A Fig** with the respective SDS-PAGE analysis of the relevant fractions.

The PilZ_Δ107-117_-FimX_GGDEF-EAL_ crystal presents the same two principal modes of crystal lattice contacts between PilZ and the FimX EAL domain observed in the previously published PilZ-FimX_EAL_ crystal structures from *X*. *citri* and *X*. *campestris* (**Fig 3A–3C**), in spite of the different fragments used and different space groups [24,30]. We name these two modes of contact interfaces 1 and 2. The most significant difference among the three structures has to do with the orientation of the first helix in FimX_EAL_ domains: in the *X*. *citri* PilZ-FimX_EAL_ and PilZ_Δ107-117_-FimX_GGDEF-EAL_ complexes (residues 429–454 and 436–454, respectively), the helix is oriented so as to contribute significantly to the interaction with PilZ in interface 1 (362 or 308 Å^2^ and 234 Å^2^ of buried surface, respectively) while this helix is rotated approximately 140^o^, pointing away from PilZ in the *X*. *campestris* PilZ-FimX_EAL_ structure [30] (**Fig 3D and 3E**). If the contribution of this helix is ignored, then the two interfaces bury similar amounts of surface area (**Fig 3E**). There are significant differences in the nature of the interfaces, however. While interface 1 involves a β-sheet extension and tenuous contacts between PilZ and the c-di-GMP ligand bound to FimX [24,30], interface 2 is dominated by hydrophobic interactions.

**S5 Fig** shows that the PilZ residues involved in contacts with FimX_EAL_ via interface 1 overlap significantly with PilZ residues involved in its interactions with PilB. Since we have previously reported that a ternary PilB-PilZ-FimX_GGDEF-EAL_ complex can be detected in far-western overlay assays, and that mutating conserved PilZ residue Trp69 to alanine (located at interface 1) reduces interactions with PilB but does not abolish PilZ-FimX_EAL_ interactions [23,24] we made a set of PilZ mutants to test the relevance of interface 2 for the stability of the PilZ-FimX interaction. **Figs 3F** and **S6** show that mutations in PilZ residues Ile10, Phe49 and Leu51 (mutants I10E and F49E/L51E) significantly reduce the stability of the PilZ-FimX_GGDEF-EAL_ binary complex while the PilZ interaction with PilB_1-190_ is maintained. These mutants have thermal stabilities (66 ^o^C and 61 ^o^C, respectively) very similar to that of wild-type PilZ (61 ^o^C) (**S7 Fig**). On the other hand, simultaneously mutating non-interfacing PilZ residues D46 and E47 to alanine does not interfere with its interactions with FimX_GGDEF-EAL_ or with PilB_1-190_ (**Figs 3F** and **S6**), even though it presents a lower Tm (48.8 ^o^C) than wild-type PilZ (**S7 Fig**). We note that the binary complexes employing wild-type and D46A/E47A PilZ seem to be stabilized by the addition of c-di-GMP (**Figs 3F** and **S5**). Together, these observations raise the possibility that interface 2 between PilZ and FimX EAL domains may be physiologically relevant. Importantly, this second mode of contact would allow the simultaneous interaction of PilZ with both FimX and PilB as shown in **Fig 2F**. This hypothesis was tested in the experiments described below.

### PilZ bridges PilB and FimX to form a ternary complex

**Fig 4:**
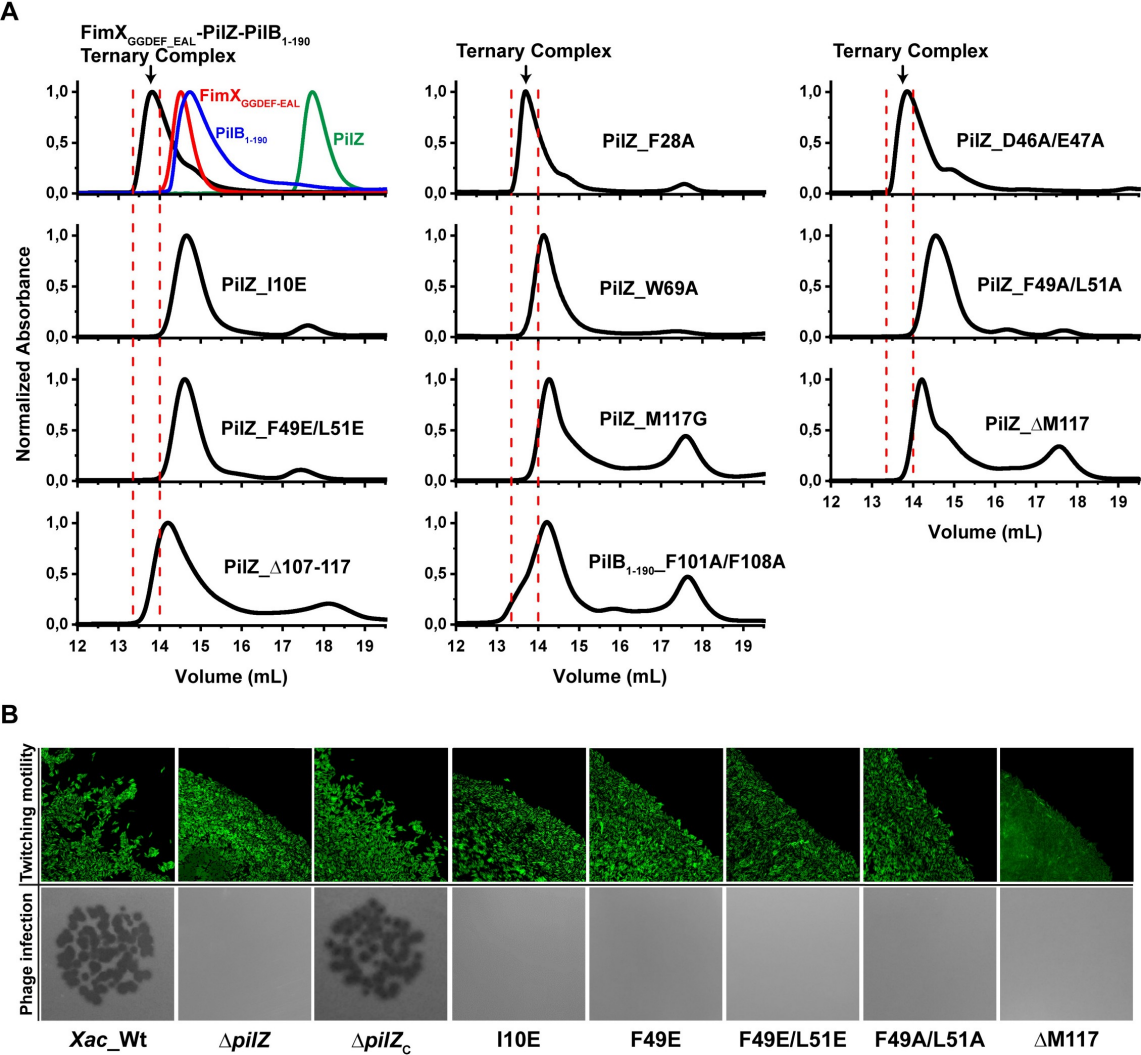
Residues important for PilB_1-190_-PilZ-FimX_GGDEF-EAL_ ternary complex stability and T4P function. **A)** Size exclusion chromatography (Superdex 200 resin, 10/300 column) analysis of PilB_1-190_-PilZ-FimX_GGDEF-EAL_ ternary complexes containing PilZ or PilB mutants. A set of mutants: PilZ_F28A_, PilZ_D46A/E47A_, PilZ_I10E_, PilZ_W69A_, PilZ_F49A/L51A_, PilZ_F49E/L51E_, PilZ_M117G_, PilZ_ΔM117_, PilZ_Δ107–117_ and PilB_1-190_F101A/F108A_ (indicated in each chromatogram panel) were used to test their effect on ternary complex stability. In all the cases, a 1:1:1 PilB_1-190_:PilZ:FimX_GGDEF-EAL_ molar ratio was used and the elution profile of the mixture is shown as a black line. All the mutants, except PilZ_F28A_ and PilZ_D46A/E47A_, affect the stability of the FimX_GGDEF-EAL_-PilZ-PilB_1-190_ ternary complex. In all of the panels, the elution volume for the ternary complex is delimited by the vertical red broken lines. In the first panel, the elution profiles for wild type complex (black line) is shown together with the profiles for the three individual components FimX_GGDEF-EAL_ (red line), PilB_1-190_ (blue line) and PilZ (green line). Each experiment was performed at least three times and representative results are shown. **B)** *Top row*: Fluorescence microscopy images of the edges of the twitching zones at the interstitial surface between the agar medium and the glass base of the microscopy chamber. For visualization, *X*. *citri s*trains were transformed with the pBBR2-GFP plasmid. Wild-type cells, that are able to twitch, can separate from the main body of the colony and migrate on their own or in small groups, producing a rough, less organized boundary between the dense interior of the colony and the surrounding medium. Cells with mutations that compromise T4P function are not able to separate from the main body of the colony and so the colony border is much smoother and well-defined. *Bottom row*: Phage ΦXacm4-11 infection assays. Dark plaques are indicative of phage-induced bacterial lysis in a confluent culture background. For both twitching motility and bacteriophage infection assays: *X*. *citri* wild type (*Xac*_Wt), *Xac*_Δ*pilZ*_XAC1133_ (Δ*pilZ*) and *Xac*_Δ*pilZ*_XAC1133_ complemented with a plasmid (pURF047) directing the expression of the wild-type PilZ protein (Δ*pilZ*c) or one of the following PilZ mutants: PilZ_I10E_, PilZ_F49E_, PilZ_F49E/L51E_, PilZ_F49A/L51A_,and PilZ_ΔM117_. **S14 Fig** shows that PilZ_I10E_, PilZ_F49E_, PilZ_F49E/L51E_, PilZ_F49A/L51A_, PilZ_ΔM117_ are all detected with anti-PilZ antibodies in these strains.

When employing PilB_12-163_ or PilB_1-190_, ternary complexes can be observed in SEC experiments using PilZ and FimX_EAL_ or FimX_GGDEF-EAL_ fragments, but not when using full-length FimX or FimX_PAS-GGDEF-EAL_ (a FimX fragment (residues 153–689) lacking the N-terminal REC domain) (**S8 Fig**). We therefore determined if mutations in the PilZ-FimX and PilB-PilZ interfaces affect the stability of the PilB_1-190_-PilZ-FimX_GGDEF-EAL_ complex. Indeed, mutations in PilZ residues participating in PilZ-FimX interface 2 (I10E, F49A/L51A, F49E/L51E) abolish ternary complex formation (**Fig 4A**). Furthermore, mutations in these PilZ residues (I10E, F49E, F49E/L51E, and F49A/L51A) compromised *X*. *citri* twitching motility and the infection of these cells by the bacteriophage ΦXacm4-11 (**Fig 4B**). On the other hand, simultaneously mutating PilZ residues Asp46 and Glu47 that are next to both PilZ-FimX_EAL_ interfaces but not directly participating in either one of them, did not compromise the stability of the PilB_1-190_-PilZ-FimX_GGDEF-EAL_ complex (**Fig 4A**). Furthermore, mutants that destabilized the PilB_1-190_-PilZ binary complex (W69A, M117G, ΔM117 and Δ107–117 in PilZ and the F101A/F108A mutant in PilB) also destabilize the ternary PilB_1-190_-PilZ-FimX_GGDEF-EAL_ complex (**Fig 4A**). Finally, the W69A, ΔM117 and Δ107–117 mutations in PilZ that destabilize its interactions with PilB_1-190_ and mutations in PilZ residues involved in interactions with FimX (I10E, F49E, F49E/L51E, and F49A/L49A) severely compromised *X*. *citri* twitching motility and the infection of these cells by the bacteriophage ΦXacm4-11 (**Fig 4B** and reference [4]). The above results clearly show that PilB, PilZ and FimX can form ternary complexes that involve the simultaneous interaction of PilZ with the N-terminal domain of PilB and the C-terminal EAL domain of FimX. A model for the PilB_12-163_-PilZ-FimX_EAL_ complex, arrived at by using PilZ as a reference to superpose the PilB_12-163_-PilZ structure and the PilZ-FimX_EAL_ structure based on interface 2, is shown in **Fig 2F**.

### Full-length PilB-PilZ-FimX complexes

**Fig 5:**
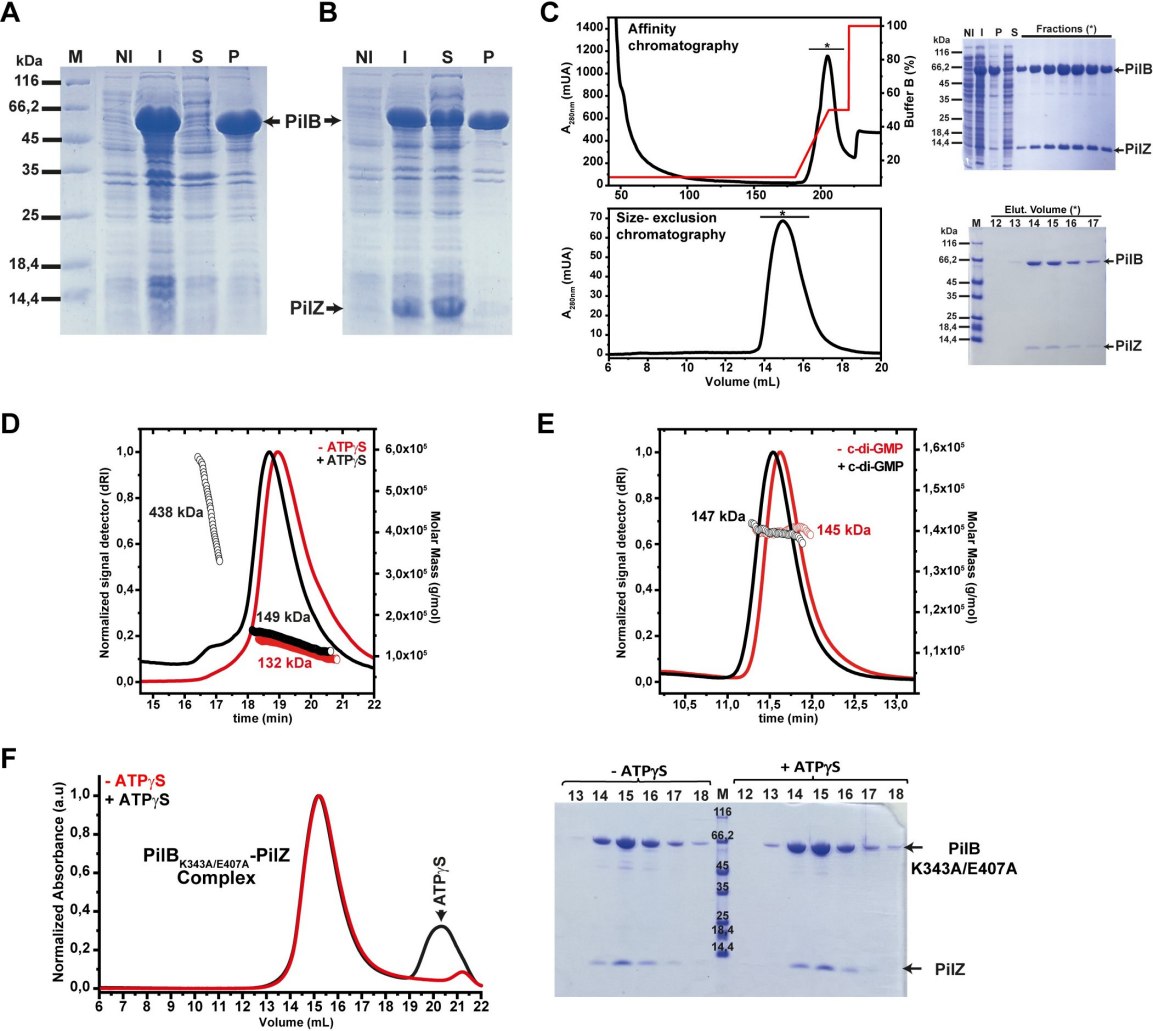
Expression of a soluble form of full-length PilB requires the co-expression of PilZ. **A-B)** SDS-PAGE analysis of *E*. *coli* cells expressing full-length PilB (**A**) and both PilB and PilZ (**B**). Lane labels: ‘M’: molecular weight markers, with the molecular weight for each band shown on the left; ‘NI’: total cell lysate before addition of IPTG; ‘I’: total cell lysates after induction with 0.2 mM IPTG; ‘S’: soluble fraction after cellular lysis; ‘P’: insoluble fraction after cellular lysis. **C)** Purification of the PilB-PilZ complex by affinity chromatography (Ni^2+^ HiTrap column; *above*) and size-exclusion chromatography (Superose 6 resin, 10/300 column; *below*). In these experiments, PilB contains an N-terminal polyhistidine tag. The elution profiles for each chromatography step are shown along with SDS-PAGE analysis of the relevant fraction. **D)** SEC-MALS analysis for the PilB-PilZ complex in the absence (continuous red line) and presence (continuous black line) of ATPγS (non-hydrolysable ATP analog). The red and black open circles show the calculated molecular mass distributions. **E)** SEC-MALS analysis for full-length FimX in the absence (continuous red line) and presence (continuous black line) of c-di-GMP. The red and black open circles show the calculated molecular mass distributions. In **D** and **E**, a silica-based WTC-050S5 (7.8/300) column (Wyatt Technology) was used. **F**) *Left*: SEC analysis of the PilB_K343A/E407A_-PilZ complex in the absence (continuous red line) and presence (continuous black line) of ATPγS (non-hydrolysable ATP analog). *Right*: SDS-PAGE analysis of the relevant fractions containing the PilB_K343A/E407A_-PilZ complex. In this experiment, a Superose 6 column (10/300, GE) was used.

Recombinant full-length *X*. *citri* PilB containing an N-terminal polyhistidine tag (theoretical MW of 64.5 kDa) is insoluble when expressed on its own in *E*. *coli* (**Fig 5A**) but is soluble when co-expressed with PilZ (theoretical MW of 12.4 kDa) (**Fig 5B**). This full-length PilB-PilZ complex is stable and can be purified by affinity and size-exclusion chromatography (**Figs 2G and 5C**). The complex elutes as a broad peak with molecular weight, estimated by SEC-MALS analysis, that varies between 100 kDa and 140 kDa (**Fig 5D**). However, in the presence of ATPγS, the elution volume of this peak shifts slightly with an estimated molecular weight of 120–160 kDa (**Fig 5D**). These molecular weights are suggestive of a dynamic equilibrium between PilB-PilZ heterodimers (theoretical MW of 77.4 kDa) and (PilB-PilZ)_2_ heterotetramers (theoretical MW of 154.8 kDa). Furthermore, the addition of ATPγS results in the appearance of a minor higher molecular weight peak with a large molecular weight distribution (between 350 and 600 kDa), indicative of larger aggregates (**Fig 5D**). These observations are consistent with previous reports on isolated PilB homologs from other systems that were observed as monomers, dimers and hexamers [12,39–45].

Full-length *X*. *citri* FimX on its own has a molecular weight estimated by SEC-MALS of 145 and 147 kDa, in the absence and presence of its c-di-GMP ligand, respectively (**Fig 5E**). These molecular weights are consistent with dimer formation (theoretical MW of 152 kDa) as previously reported for the homologous protein in *P*. *aeruginosa* [29]. In contrast to results using PilB N-terminal fragments, when using full-length PilB, we are able to clearly observe ternary PilB-PilZ-FimX complexes containing full-length FimX **(Fig 2G**). These results indicate that the complete PilB protein is required for the stable incorporation of full-length FimX into the complex. SEC-MALS analysis of the PilB-PilZ-FimX complex again points to a heterogeneous mixture with molecular weight varying between 500 and 650 kDa (**S9A Fig**). The addition of ATPγS and c-di-GMP seems to stabilize this higher molecular weight complex (**Figs 2G and S9A**). We note that a PilB-PilZ-FimX stoichiometry of 6:6:2 corresponds to a theoretical molecular weight of approximately 620 kDa (see Discussion below).

### FimX enhances PilB ATPase activity

PilB proteins from other bacterial species have been shown to exhibit ATPase activity *in vitro* [12,40,46]. **Fig 2H** shows that the *X*. *citri* PilB-PilZ complex can hydrolyze ATP *in vitro* with an intrinsic activity of approximately 8.5 nmol ATP hydrolyzed/min per mg PilB-PilZ complex, under the conditions tested. Upon addition of full-length FimX, an approximately 3-fold increase in ATPase activity was observed (22.5 nmol/min per mg PilB-PilZ complex; **Fig 2H**). As expected, no significant activity was observed for the PilB-PilZ and PilB-PilZ-FimX complexes containing PilB_K343A/E407A_ in which two active site residues, located within the ATPase domain, were mutated to alanine (**Fig 2H**). **Fig 5F** shows that this PilB mutant retains the ability to form a binary complex with PilZ but no mobility shift or evidence of higher order oligomers is observed upon addition of ATPγS. Finally, **Figs 2G and S9B** show that ternary complexes containing the PilB_K343A/E407A_ mutant, PilZ and FimX do form; but the higher-order oligomers seem to be less stable. These results show that i) FimX stimulates PilB ATPase activity, ii) the incorporation of FimX into the complex is not absolutely dependent on PilB ATPase activity and iii) PilB ATPase activity stabilizes higher order oligomeric forms of the PilB-PilZ binary and PilB-PilZ-FimX ternary complexes.

### Unipolar colocalization of PilB, PilZ, FimX and PilQ at the leading edge of twitching *X. citri* cells

**Fig 6:**
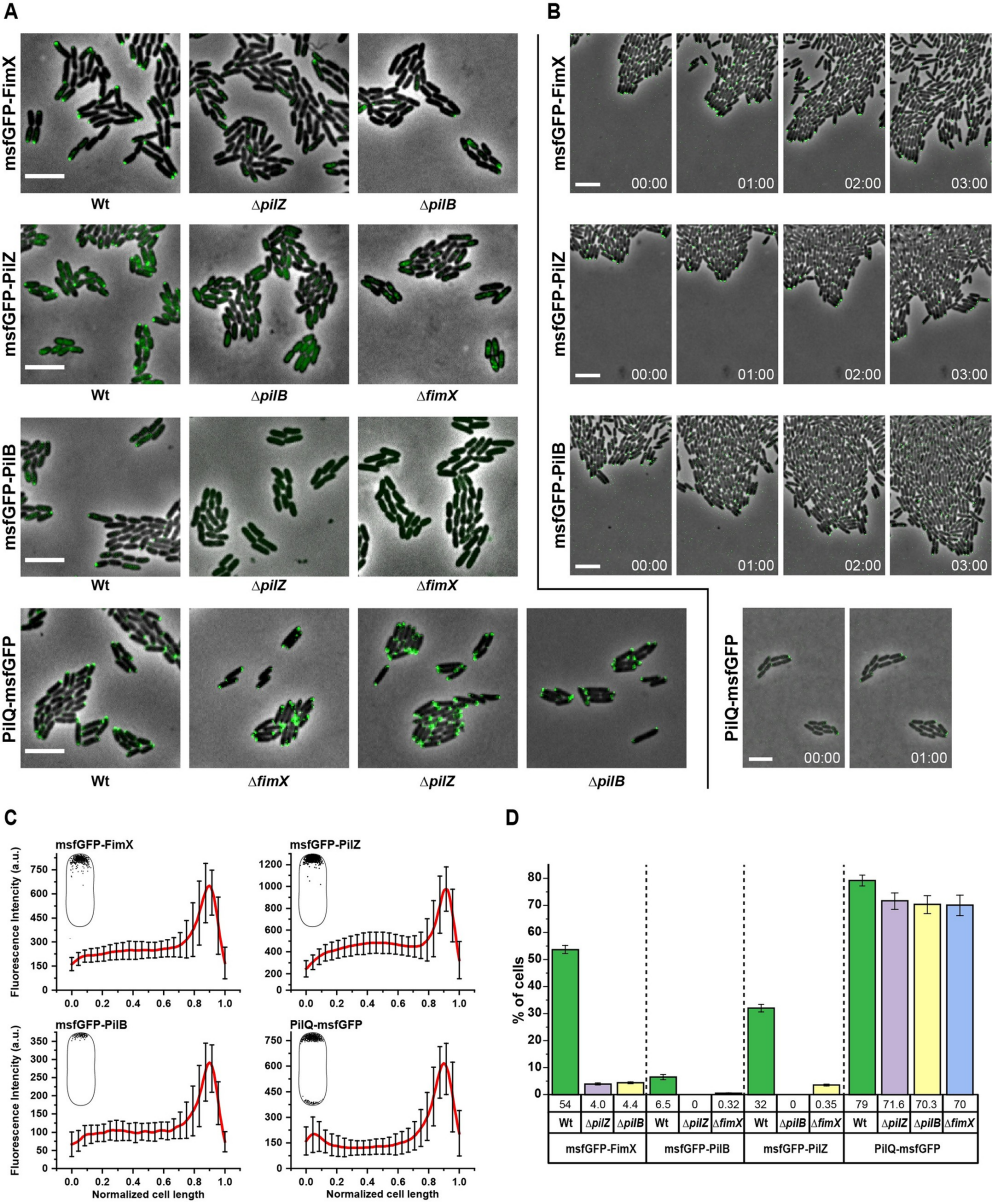
Subcellular localization of FimX, PilZ, PilB and PilQ. **A)** Representative images of fluorescent light microscopy of *X*. *citri* cells expressing chromosomal FimX, PilZ, PilB and PilQ fused with msfGFP (N-terminal fusion in FimX, PilZ and PilB and C-terminal fusion in PilQ) in wild type and mutants strains with deletion in indicated T4P regulatory components. The *X*. *citri* strains expressing the fluorescent fusion proteins were imaged by phase contrast and epifluorescence microscopy and analyzed at least five times independently with similar results. In these experiments, cells were grown on KB-agarose pads (1.5% w, using 0.2% casamino acids as nitrogen source) supplemented with 2 mM CaCl_2_. Scale bar, 3 μm. **B)** Representative time lapse images showing that msfGFP-FimX, msfGFP-PilZ, msfGFP-PilB and PilQ-msfGFP localize to the leading pole in wild type *X*. *citri* cells undergoing twitching motility. The time lapse interval (h) is indicated for each frame. Images were taken by using an epi-fluorescence light microscope. Scale bar, 5 μm. See also **S5–S8 Movies** for time lapse images at shorter intervals. **C)** Graphical representation of the fluorescence intensity profile over the length of *X*. *citri* cells expressing msfGFP-FimX, msfGFP-PilZ, msfGFP-PilB and PilQ-msfGFP. The fluorescence intensity profile was obtained from 100 individual *X*. *citri* cells for each case (cell length was normalized). The profiles of all individual cells are shown in **S10A Fig**. Insets show the foci localizations. **A** and **B** show that msfGFP-FimX, msfGFP-PilZ and msfGFP-PilB foci exhibited unipolar localization. PilQ-msfGFP exhibits a mixture of both unipolar and bipolar localization; in the latter case one of the foci was almost always much more intense than the other (see **S10A Fig**). **D)** Comparison of the frequency of polar localization of msfGFP-FimX, msfGFP-PilZ, msfGFP-PilB and PilQ-msfGFP foci in wild-type, Δ*fimX*, Δ*pilZ* and Δ*pilB* backgrounds.

In order to study the localization of the *X*. *citri* Type IV pilus components, we produced strains in which the FimX, PilZ, PilB and PilQ genes were replaced with msfGFP-FimX, msfGFP-PilZ, msfGFP-PilB and PilQ-msfGFP fusions, respectively. **Figs 6** and **S10A** show that FimX, PilZ and PilB all are localized predominantly at a single cell pole. The fraction of cells exhibiting polar foci was 54% for FimX, 32% for PilZ but only 6.5% for PilB under the conditions tested (**Fig 6D**). The outer membrane secretin subunit PilQ is also found mostly at one pole (79%) with a small fraction of cells showing foci at two poles simultaneously (**Fig 6C and 6D**). In the cases where bipolar PilQ localization was observed, one of the foci was almost always much more intense than the other (see **S10A Fig** for fluorescence intensity distributions along the major axes of all individual cells analyzed).

In order to determine if protein localization is influenced by whether the cells are grown in liquid (no twitching) or semisolid media (twitching) we grew mCherry-FimX and PilQ-msfGFP cells in liquid medium and observed them immediately after transfer to KB-agarose slabs (before they have a chance to begin twitching, time = 0 hours) and 6 hours after transfer during which time they are exhibiting twitching behaviour. **S10B Fig** shows that no mCherry-FimX foci are observed in cells immediately after transfer from liquid culture and that the foci appear only after growth for some time on semisolid media. In contrast, it was very common to observe PilQ-msfGFP foci at both cell poles (bipolar localization) immediately after growth in liquid medium; but after several hours growth on semi-solid media, most cells exhibited unipolar PilQ-msfGFP foci (**S10B Fig**). **S11 Fig** and **S1–S4 Movies** show that these *X*. *citri* strains all exhibit normal twitching motility and can all be infected with bacteriophage ΦXacm4-11, *X*. *citri* phenotypes that are dependent on a functional T4P [4].

Time-lapse fluorescence microscopy images of actively twitching *X*. *citri* msfGFP-FimX, msfGFP-PilZ, msfGFP-PilB and PilQ-msfGFP strains reveal that the most intense foci are found at the leading poles of the cells at the leading edge of the group of migrating cells (**Fig 6B and S5–S8 Movies**). These strains were then used to introduce deletions in specific Type IV pilus components. **Fig 6A and 6D** shows that deletion of *pilZ* or *pilB* genes results in a drastic reduction in the number of cells with fluorescent msfGFP-FimX foci. Likewise, deletion in the *pilZ* or *pilB* genes result in drastic reductions in the unipolar localization of msfGFP-PilB or msfGFP-PilZ, respectively (**Fig 6A and 6D**). In contrast, deletion of *fimX*, *pilZ* or *pilB* genes did not affect the polar localization PilQ-msfGFP (**Fig 6A and 6D**).

**Fig 7:**
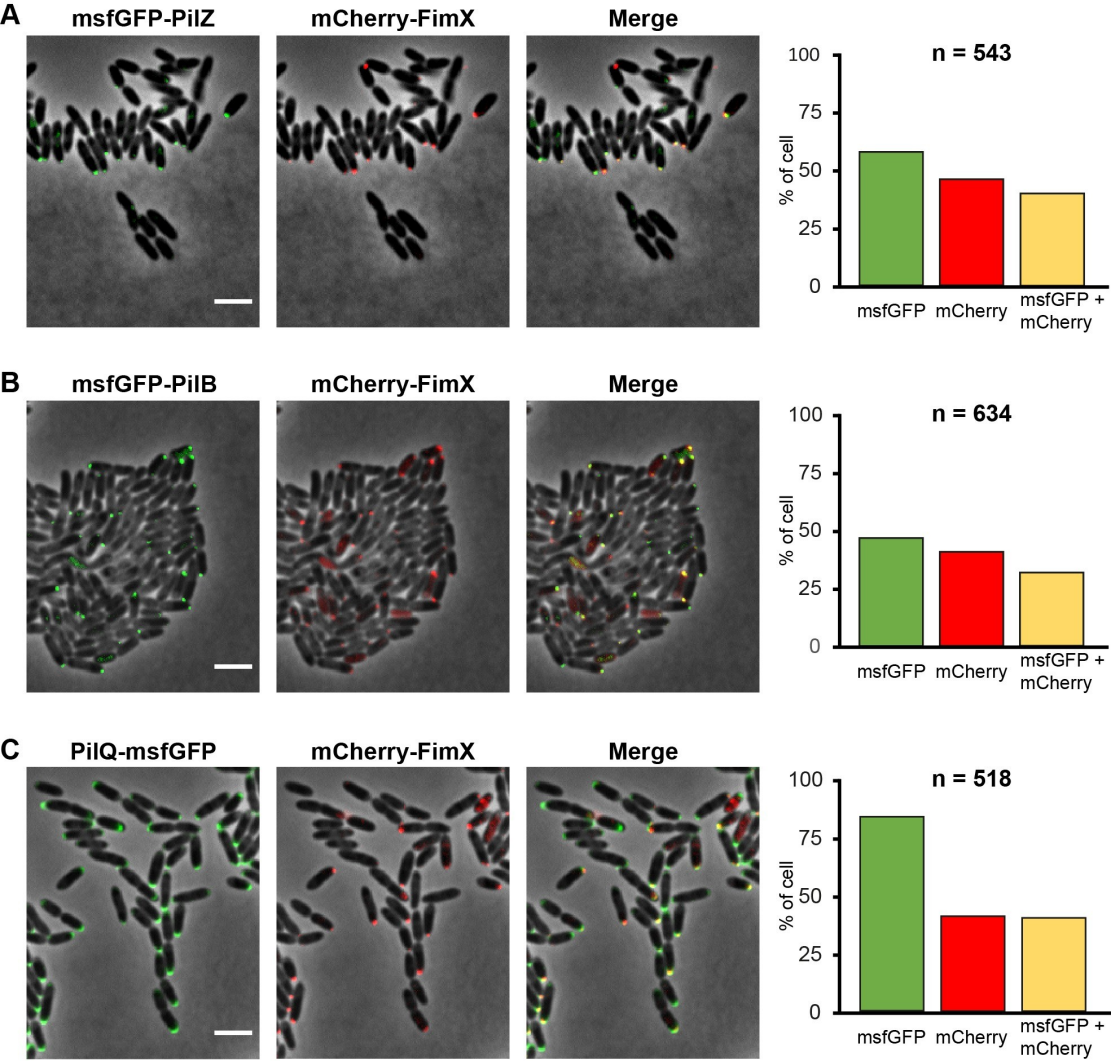
Subcellular Colocalization of FimX with PilZ, PilB and PilQ. Representative images of wild type *X*. *citri* cells co-expressing chromosomal msfGFP-PilZ and mCherry-FimX (**A**), msfGFP-PilB and mCherry-FimX (**B**) and PilQ-msfGFP and mCherry-FimX (**C**). *X*. *citri* expressing the fluorescent fusion proteins were imaged by phase contrast and epifluorescence microscopy at least three times with similar results. Scale bar, 3 μm. To the right, bar graphs present the fraction of cells presenting msfGFP, mCherry and both msfGFP and mCherry polar foci. the total number of cells analyzed (n) is given.

To confirm that FimX, PilZ, PilB and PilQ are in fact all localized at the same pole, mCherry-FimX fusions were used to substitute the FimX gene in the msfGFP-PilZ, msfGFP-PilB and PilQ-msfGFP strains to produce cells in which two T4P components could be simultaneously localized by fluorescence microscopy. These three strains are all able to be infected by ΦXacm4-11, indicative of normal T4P function (**S11B Fig**). **Fig 7** shows fluorescence microscopy images of groups of *X*. *citri* cells in which most of the cells displaying mCherry-FimX foci also display msfGFP-PilB, msfGFP-PilZ or PilQ-msfGFP foci at the same pole. These results are consistent with studies on the co-localization of FimX, PilB and PilQ homologs in *P*. *aeruginosa* [25,26,34,47].

## Discussion

The crystal structures of the N-terminal domains of four relatively distant PilB homologs are available in the Protein Data Bank (PDB): *V*. *cholerae* MshE_Nt_ (PDB: 5HTL), the *X*. *campestris* T2SS protein XpsE_Nt_ (PDB: 2D28), *V*. *vulnificus* T2SS GspE_Nt_ (PDB: 4PHT) and *V*. *cholerae* T2SS EpsE_Nt_ (PDB: 2BH1), the latter two in complex with the cytoplasmic domains of the PilM paralogs GspL and EpsL, respectively. These structures show a very similar topology in spite of rather poor sequence alignment with *X*. *citri* PilB_12-163_ (**S12 and S13 Figs**). MshE_Nt_ and XpsE_Nt_ contain both ND0 and ND1 sub-domains and align with *X*. *citri* PilB_12-163_ with r.m.s.d. of 3.3 Å and 3.1 Å, respectively. Interestingly, *V*. *cholerae* MshE_Nt_ binds the cyclic dinucleotide c-di-GMP [21,48]. However, residues involved in nucleotide binding [21] are not conserved in *X*. *citri* PilB and its homologs found in the genera listed in **S3 Table**. The N-terminal domains of GspE from *V*. *vulnificus* and EpsE from *V*. *cholerae* both lack the ND0 sub-domain and the structural alignment of their ND1 sub-domains with PilB_12-163_ have r.m.s.d.s of 1.9 Å and 2.5 Å, respectively (**S12C Fig)**.

The NMR spectrum of *X*. *citri* PilB_12-163_ indicates that it is at least partially disordered on its own and becomes fully folded upon interaction with PilZ. This conformational heterogeneity is consistent with the following observations: i) The XpsE N-terminal domain has been crystallized in two conformations in which the ND0 sub-domain is found in an open, less-compact configuration or in a closed configuration [35] that more closely resembles that observed in *X*. *citri* PilB_12-163_ in complex with PilZ. ii) The crystal structures of full-length PilB proteins from *Geobacter metallireducens* and *G*. *sulphurreducens* lacked observable electron density for their ND0/ND1 domains [35,36,44]. Both proteins are predicted to bind c-di-GMP, though the cyclic nucleotide was not included during crystallization. iii) The cryoEM structures of PilF from *Thermus thermophilus* only produced maps of very low resolution for the second and third ND0/ND1 domains while the first was not visible at all [49]. Again, two of the PilF ND0/ND1 domains have been shown to bind c-di-GMP [21,50,51] but the cyclic nucleotide was not included during cryo grid preparation. The physiological relevance of PilZ-induced folding of the N-terminal domain is not clear at the moment since we do not know whether the PilB-PilZ interaction is static or if PilZ dissociates and reassociates at some stage in the ATPase/polymerization cycle or during a signaling pathway that leads to assembly or disassembly of the PilB hexamer. Our estimation of the dissociation constant of the PilB_1-190_-PilZ_W69_5OHW_ complex in the nanomolar range makes PilZ dissociation from PilB unlikely.

In addition to its interactions with PilB, *X*. *citri* PilZ also interacts with the C-terminal EAL domain of FimX. Interface 2 observed in the different crystal forms of the *X*. *citri* PilZ-FimX_EAL_ and PilZ_Δ107-117_-FimX_GGDEF-EAL_ complexes and the *X*. *campestris* PilZ-FimX_EAL_ complex (**Fig 3**) allowed us to propose a model for the PilB-PilZ-FimX ternary complex (**Fig 2F**) that is consistent with a large amount of structural, biochemical, cellular and genetic data described above. We note that while homologous PilZ-FimX interactions have been observed for the highly similar proteins in *X*. *campestris* pv. *campestris* [30] and *X*. *oryzae* pv. *oryzae* [31], *in vitro* assays were not able to detect interactions between FimX and PilZ from *P*. *aeruginosa* [33]; instead, direct interactions between FimX and PilB were reported, though the precise domains of the two proteins involved in this interaction were not identified [34]. We therefore speculate that there are probably strong parallels in the *Pseudomonas* and *Xanthomonas* systems but that binary interactions between purified components from the different species may have different *in vitro* affinities that may make them difficult to detect out of the context of the native T4Ps.

At this point it is worth considering the orientation of the PilB-PilZ-FimX complex with respect to other T4P components embedded in or associated with the inner membrane (the so-called inner membrane platform). The orientation of the PilB hexamer and its T2SS homologs has been proposed based on crystal structures of the *G*. *metallireducens* PilB ND2-ATPase fragment hexamer in the presence of ADP and the non-hydrolysable ATP analogue AMP-PNP [34,36], the GspE-GspL complex from *V*. *vulnificus* [52] and cryo-tomography maps of the *M*. *xanthus* T4P [9] and the *Legionella pneumophila* T2SS [53]. In all of these models, the plane of the PilB hexameric ring is parallel with that of the membrane, with the C-terminal ATPase domain oriented towards the cytosol and the ND2 domain pointing towards the membrane. Since the ND0/ND1 and ND2 domains are connected by what is expected to be a negatively charged flexible linker (see below), the ND0/ND1 domain is expected to be free to make contacts with interaction partners in the inner membrane platform. One strong candidate to mediate such interactions is PilM since interactions between PilB and PilM homologs have been observed for T4P of *M*. *xanthus* [54], *T*. *thermophilus* [41], *P*. *aeruginosa* [55], the bundle-forming pilus machinery of enteropathogenic *E*. *coli* [56] and the T2SS of *X*. *campestris* [35]. Furthermore, complexes between PilB and PilM homologs in T2SS have been crystallized: full-length ATPase GspE with the cytosolic domain of GspL from *V*. *vulnificus* [52] and EpsE ND1 domain with the cytosolic domain of EpsL from *V*. *cholerae* [57]. **S13 Fig** shows the superposition of the *X*. *citri* PilB_12-163_-PilZ complex with that of the GspE-GspL and EpsE_ND1_-EpsL complexes using the ND1 sub-domains as reference. In both GspE-GspL and EpsE_ND1_-EpsL structures, the PilM homolog (GspL or EpsL) binds to a surface of the PilB homolog (GspE or EpsE) which we name the PilM interface of PilB. Importantly, the PilM interface does not overlap with the PilB surface to which *X*. *citri* PilZ binds (called the PilZ interface in **Figs 2E and S13**). The residues on this ND1 surface are particularly well conserved in PilB proteins from organisms that also code for PilZ and FimX homologs (**Fig 2C**).

**Fig 8:**
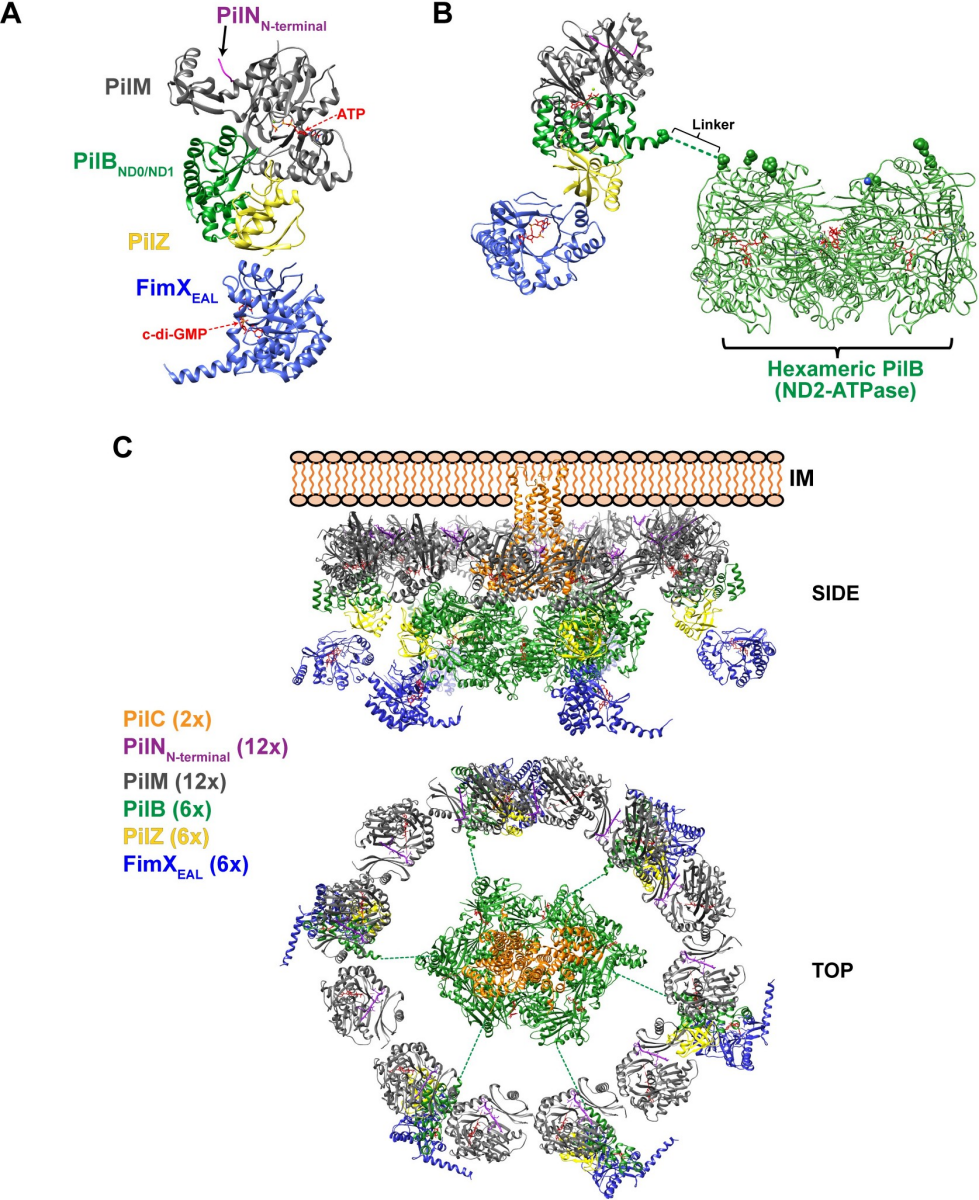
The PilB-PilZ-FimX complex in the context of the inner membrane platform of the Type IV pilus. **A)** Model for interactions between the PilB_ND0/ND1_-PilZ-FimX_EAL_ complex and the PilM-PilN_N-terminal_ complex. PilB_ND0/ND1_ (green) and PilZ (yellow) are shown as determined in the PilB_12-163_-PilZ structure (present study). FimX_EAL_ (blue) is placed as observed in interface 2 of the *X*. *citri* PilZ_Δ107-117_-FimX_GGDEF-EAL_ (this study), *X*. *citri* PilZ-FimX_EAL_ [24] and the *X*. *campestris* pv. *campestris* PilZ-FimX_EAL_ [30] complexes (see **Fig 3**). The homology model for *X*. *citri* PilM (see Materials and Methods) was oriented with respect to PilB_ND0/ND1_ based on the *V*. *vulnificus* GspE-GspL [52] and *V*. *cholerae* EpsE-EpsL [57] crystal structures (see **Figs 2E** and **S13**). **B)** The PilB_ND0/ND1_ domain is connected to the hexameric PilB core (made up of ND2 and ATPase domains) via a highly acidic and glycine-rich linker (approximately 30 residues; see **Fig 2A**). The homology model of the *X*. *citri* PilB hexameric core was built based on the *G*. *metallireducens* PilB core structure as described in Materials and Methods. **C)** Depiction of possible interactions of the PilB-PilZ-FimX_EAL_complex with the proposed PilM-PilN dodecamer and PilC dimer based on the cryo-electron tomography model of the *M*. *xanthus* T4P [9]. The homology model of the PilC dimer (depicted in orange) was built as described in Materials and Methods. Coloring scheme for the other subunits is the same as in part **A**. Top and side views are shown. Note that the REC, PAS and GGDEF domains of FimX are not shown and that *X*. *citri* (this study) and *P*. *aeruginosa* FimX is a homodimer, probably due to interactions between N-terminal REC domains [29]. Here the model assumes a PilB-PilZ-FimX stoichiometry of 6:6:6. However, since the PilB hexamer may exhibit C2 symmetry, stoichiometries of 6:6:4 or 6:6:2 are also possible (see main text).

The above considerations allow us to envision how the PilB-PilZ-FimX complex can interact with the inner membrane platform via PilM which is in turn bound to the cytosolic N-terminal portion of the integral membrane protein PilN [55,58] as depicted in **Fig 8**. Studies in *M*. *xanthus* suggest that PilM and PilN form a dodecameric ring around a concentric PilB hexamer [9]. We used this architecture to incorporate our model of the PilB_ND0/ND1_-PilZ-FimX_EAL_ complex. In this model, the central hexameric core made up of PilB ND2 and ATPase domains is surrounded by six copies of the PilM-PilN-PilB_ND0/ND1_-PilZ-FimX_EAL_ complex. Here, we point out that the PilB ND1 sub-domain is separated from the ND2 domain by a flexible linker of approximately 30 amino acids that is rich in acidic residues (**Fig 8B**). For example, the sequence from positions 161 to 193 in *X*. *citri* PilB (**DDEEG**M**GD**L**D**VSA**GDED**M**G**A**GGD**S**G**V**D**AK**GDD**T) contains 8 glycines, 10 aspartates, 3 glutamates and only one positively charged lysine (**Fig 2A**). This suggests that the orientation of the ND0/ND1 domain of PilB with respect to the central hexameric core made up of ND2 and ATPase domains could be highly variable or at least susceptible to modulation by other regulatory components. Furthermore, each PilN-PilM-PilB_ND0/ND1_-PilZ-FimX_EAL_ complex is shown to alternate with another PilM-PilN complex (**Fig 8C**). Finally, the central lumen of the hexameric core of PilB is thought to accommodate the cytosolic domains of the dimeric integral membrane protein PilC (**Fig 8C**) to which conformational changes induced by ATP hydrolysis are coupled so as to promote the incorporation of pilin subunits into the base of the growing pilus [36,59,60].

The interactions depicted in the model shown in **Fig 8** immediately suggest possible schemes by which FimX and PilZ may regulate T4P function. Such mechanisms would eventually take into account the binding of ATP by PilM, PilM binding to PilN and PilB (as well as PilT and PilC [55]), the binding of c-di-GMP to FimX, the possibility of alternative PilZ-FimX binding modes (via interfaces 1 and 2), the conformational flexibility of the PilB N-terminal domain and the conformational flexibility afforded by the acidic and glycine-rich linker between ND1 and ND2, the structural transitions induced during ATP binding and hydrolysis by PilB [36] and PilB interactions with PilC. In this model we assume that one PilZ binds to each PilB ND0/ND1 domain, but the stoichiometry of FimX binding to the PilB-PilZ complex is not at the moment clear. One possibility is that one FimX binds to each PilZ-PilB resulting in a PilB-PilZ-FimX stoichiometry of 6:6:6 as depicted in **Fig 8C**. However, the crystal structures of most PilB homologs are hexameric rings with C2 symmetry, in which three different subunit conformations are adopted, one by each pair of opposing subunits. This opens up the possibility that FimX has different affinities for each of the three conformational states of the PilB-PilZ units in the hexamer and, assuming that FimX dimers are maintained, alternative stoichiometries such as 6:6:4 and 6:6:2 are possible. Structural transitions induced by c-di-GMP binding have been observed in FimX from *P*. *aeruginosa* [61] as well as in the present study for *X*. *citri* (**Fig 5E**). Thus, c-di-GMP-induced transitions in FimX could in principle be transferred, through PilZ and the PilB ND0/ND1 domain, to the ND2-ATPase domains in the internal PilB hexameric ring and in this way modulate ATPase activity, its interactions with PilC and the rate of pilus subunit incorporation at the base of the growing pilus. Our observations that FimX promotes the polar localization and ATPase activity of the PilB-PilZ complex is consistent with this general idea. Since depolymerization of the pilus probably requires the partial dissociation of the PilB-PilZ-FimX complex from the T4P platform to give way to the homo-hexameric complex of PilT (and/or PilU) [55,62], one could envision a scheme by which structural transitions in the PilB-PilZ-FimX interface could be transmitted to the PilB-PilM interface so as to promote or impede PilB docking into the platform.

Most bacterial species for which T4P function has been studied in detail, such as *M*. *xanthus*, *Neisseiria* spp., *Synechocystis* sp., *V*. *cholerae*, *T*. *thermophilus*, *Clostridium perfringens and P*. *aeruginosa* seem to be regulated by different mechanisms [18,19,40,63–65]. We have characterized important interactions between the *X*. *citri* T4P ATPase PilB and its regulators PilZ and FimX. At least 50 different bacterial genera from the Gammaproteobacteria class have species whose genomes code for homologs of *X*. *citri* PilZ, FimX and PilB (only one species from each genus is included in **S3 Table** in order to avoid redundancy). We note that i) all of the PilB homologs in the list have both ND0 and ND1 sub-domains even though many from T4P or T2SS in other species lack an ND0 domain, ii) none of the ND0 sub-domains have the conserved motifs that mediate binding to c-di-GMP in other more distant PilB homologs, iii) all of the PilZ homologs belong to the PA2960/XAC1133 orthologous group [23] and iv) all FimX homologs have the same domain architecture as that of *X*. *citri* and *P*. *aeruginosa* FimX proteins. These observations suggest that in many of these genera, we can expect that PilZ and FimX orthologs interact with PilB and participate in T4P regulation in a manner similar to that described here for *X*. *citri*.

## Materials and methods

### Bacterial strains, bacterial growth and cloning

**S4 Table** shows all strains used in this study. Primer sequences and plasmids are listed in **S5 Table**. *Escherichia coli* DH5α was used for DNA cloning. The coding sequence for the different constructions of FimX_*XAC2398*_, PilZ_*XAC1133*_ and PilB_*XAC3398*_ were amplified by PCR from genomic DNA from *X*. *citri* pv. *citri* strain 306 genomic DNA and cloned into the expression vector pET28a or pET3a [66] in the NdeI and BamHI sites. The full-length PilB and PilZ were cloned into the co-expression vector pETDuet. The coding sequencing for PilB fragment (PilB_1-190_, PilB_156-307_ and PilB_156-578_) was amplified by PCR from *X*. *citri* genomic DNA (primers listed in **S3 Table**) and cloned into the two-hybrid bait vector pOBD [67] in the NcoI and XhoI sites. A series of substitutions of amino-acid residues in the PilZ and PilB were carried out by via a single-step PCR protocol using the QuickChange Site-Directed Mutagenesis kit (STRATAGENE) (primers listed in **S5 Table**). The mutants were used for expression in *E*. *coli* or for complementation of *X*. *citri* strains. The PilZ wild type and F28A, W69A and Δ107–117 mutants were previously described [23,24]. All clones were confirmed by DNA sequencing. Antibiotics were used at the following final concentrations: kanamycin 50 μg/mL, ampicillin 100 μg/mL. In order to complement the ΔpilZXAC1133 knockouts, fragments coding for wild type PilZ (XAC1133) and its mutants M117G, ΔM117, I10E, F49E, F49E/L51E, F49A/L51A were amplified and cloned into pUFR047, as described previously [23].

### Two-hybrid assays

*Saccharomyces cerevisiae* strain PJ694-a (*MAT*a *trp1*-*901 leu2*-*3*,*112 ura3*-*52 his3*-*200 gal4*Δ *gal80*Δ *LYS2*::*GAL1-HIS3 GAL2-ADE2 met2*::*GAL7-lacZ*)[68] was grown in SC medium (0.66% (w/v) nitrogen base without amino acids, 2% (w/v) glucose, 0.008% (w/v) adenine, 0.083% (w/v) amino acids mixture, pH 5.6). When necessary, SC medium was prepared lacking one or more of the components adenine, histidine, tryptophan, and leucine. Solid medium also contained 1.6% (w/v) Bacto-agar and 5 mM 3-aminotriazole. β-galactosidase activity was measured by selecting cells from SC-WL plates to inoculate SC-WL liquid cultures that were grown overnight at 30°C, collected by centrifugation, resuspended in 1 ml of YPD medium (20 g/L Tryptone, 10 g/L yeast extract, 20 g/L glucose, pH 5.8) and grown for another 4 h. Cells were collected, resuspended in the same volume of Z buffer (0.06 M Na_2_HPO_4_, 0.04 M NaH_2_PO_4_, 0.01 M KCl, 0.001 M MgSO_4_, 0.05 M β-mercaptoethanol, pH 7.0) and the A_600nm_ of the resuspended cells was measured. After that, 100 μl of chloroform and 50 μl of 0.1% (w/v) SDS were added, vortex mixed and incubated for 5 min at 28°C. The reaction was initiated by adding 0.2 ml of 4 mg/mL o-nitrophenyl-β-d-galactoside (ONPG), vortex mixed, incubated at 28°C and time measured until the reaction was terminated by the addition of 0.5 ml of 1 M Na_2_CO_3_. The solution was centrifuged for 5 min at 13000 g and the A_420nm_ and at A_550nm_ were recorded. The Miller units were calculated as: Miller Unit = 1000 [(A_420nm_− 1.75 A_550nm_)]/(TVA_600nm_) where T is reaction time (in minutes), and V is volume (in ml).

### Protein expression and purification

*X*. *citri* PilZ (XAC1133), PilZ mutants (PilZI_10E_, PilZ_Y22A_, PilZ_F28A_, PilZ_D46A/E47A_, PilZ_F49E/L51E_, PilZ_F49A/L51A_, PilZ_W69A_, PilZ_Δ107–117_, PilZ_M117G_ and PilZ_ΔM117_) and the FimX fragments (full-length FimX, FimX_EAL_, FimX_GGDEF-EAL_) were expressed and purified as described previously [23,24]. Conditions for protein expression (host *E*. *coli* strain and temperature) for FimX_GGDEF-EAL_-PilZ_Δ107–117_ complex, PilB, PilB fragments, their mutants (F77A, F101A, F108A, F101A/F108A, R103A e E132A) and PilB-PilZ complexes are summarized in **S6 Table**. Proteins were produced by growing *E coli* cells in 2×TY medium (16 g/L of bacto-tryptone, 10 g/L of yeast extract and 5 g/L of sodium chloride) supplemented with kanamycin (50 mg/mL) and/or ampicillin (100 mg/mL) under agitation of 200 r.p.m. to an OD_600nm_ of 0,6–0.8, at which point 0.4 mM isopropyl-β-D-1-thiogalactopyranoside was added. After induction, the cells were collected and resuspended in 25 mL of lysis buffer (50 mM Tris–HCl pH 8.0, 200 mM NaCl, 25% sucrose and 20 mM imidazole)/1L of culture and lysed by sonication. The lysate was centrifuged at 37,500 g, 4°C for 45 min. The soluble fraction was applied to Ni-HiTrap column (GE Healthcare) previously equilibrated in buffer A (50 mM Tris–HCl pH 8.0, 200 mM NaCl, 1mM MgCl_2_, 20 mM imidazole and 5% glycerol). The resin was washed with 10–20 column volumes of buffer A and proteins were eluted with a gradient containing 20–500 mM imidazole in buffer A over 10 column volumes. The pooled protein fractions were concentrated, using centrifugal filter devices (Millipore), and further purified by SEC using a Superdex 200 26/600 column (GE Healthcare) equilibrated in gel-filtration buffer (20 mM Tris–HCl pH 8.0, 100 mM NaCl, 1 mM MgCl_2_). PilB-PilZ complex was purified using Superose 6 10/300 column equilibrated with 20 mM Tris–HCl pH 8.0, 50 mM NaCl and 5mM MgCl_2_. Fractions containing the recombinant proteins were pooled and concentrated to 4–8 g/L using Amicon ultra concentrators (Millipore) with a 10 kDa membrane cutoff. Protein aliquots were stored at −20°C after the addition of 20% glycerol. Selenomethionine-containing protein was produced by growing a 500 ml culture in M9 medium to an OD_600nm_ of 0.8 at 37°C at which point 100 mg/L, lysine, 100 mg/L phenylalanine, 100 mg/L threonine, 50 mg/L isoleucine, 50 mg/L valine and 60 mg/L selenomethionine were added. After 15 min, 0.4 mM isopropyl β-d-1-thiogalactopyranoside (IPTG) was added and the cells were grown for 16 h at 18°C. ^15^N-labeled PilB_12-163_ was produced by growing transformed *E*. *coli* BL21(DE3) cells in M9 medium in the presence of 1 g/l of ^15^N-NH_4_Cl and 4 g/l of unlabeled glucose. PilZ containing 5-hydroxytryptophan at the unique tryptophan position (PilZ_W69_5OHW_) was expressed [69] and purified [23] as previously described.

### Crystallization and structural determination of the PilB_12-163_-PilZ complex

The crystallization of unlabeled and selenomethionine-labeled PilB_12-163_-PilZ complexes was carried out in sitting-drop plates by mixing equal volumes (1 μL) of 4 mg/ml protein and well solution (0.1 M Tris pH 8.5; 2.0 M ammonium sulfate) and growing at 18°C. For the selenomethionine-labeled crystal, X-ray radiation at 0.97889 Å was used (corresponding to the peak of the fluorescence spectrum). The native crystal dataset was collected using 1.4587 Å radiation for the crystal containing the PilB P70S mutation and 1.5418 Å radiation for the crystal containing the wild-type PilB sequence (**S1 Table**). The data were indexed and integrated using the XDS program[70]. Selenium sites were found using AutoSol [71]. The resulting electron density map was used to construct a preliminary polyalanine model with ARP/wARP [72]. Interpretation of the electron density maps and construction of the missing residues was performed with COOT [73]. Structural refinement of the model was performed using REFMAC [74], Phenix [75] and COOT. Water molecules were added automatically using REFMAC and manually using COOT. Details of the refinement data statistics are shown in **S1 Table**. Structural alignments and figures were produced using Chimera [76]. The atomic models and experimentally determined structure factors have been deposited in the protein data bank with pdb codes 7LKM, 7LKN and 7LKO.

### Crystallization and structural determination of the PilZ_Δ101-117_-FimX_GGDEF-EAL_ complex

The crystallization of native PilZ_Δ101-117_-FimX_GGDEF-EAL_ complex was performed in sitting-drop plates by mixing equal volumes (1 μL) of 10 mg/ml protein and well solution (0.1 M MES pH 6.5; 1.6 M Mg_2_SO_4_) and growing at 18°C. The data were indexed and integrated using the HKL2000 software [77]. Phases were calculated by molecular replacement using the model of the *X*. *citri* PilZ-FimX_EAL_ complex (PDB ID 4FOU; [24]). Structural refinement was performed as described above using REFMAC, Phenix and COOT. Details of the refinement data statistics are shown in **S1 Table**. Structural alignments and figures were produced using Chimera. The atomic model and experimentally determined structure factors have been deposited in the protein data bank with pdb code 7LKQ.

### Size-exclusion chromatography (SEC)

A Superdex S200 10/300 (GE Healthcare) column was used to investigate protein–protein interactions between the PilB_12-163_ or PilB_1-190_, PilZ and the different constructions of FimX. The column was previously equilibrated with 50 mM Tris-HCl pH 8,0, 50 mM NaCl, 5mM MgCl_2_. Protein samples, for binary complex (PilB_1-190_ and wild type PilZ or their mutants) and ternary complex (PilB_12-163_-PilZ and PilB_1-190_-PilZ complex with FimX fragments), were mixed at an equimolar ratio (60–100 μM) in a final volume of 100 μL and applied to the SEC column. In SEC experiments studying the binary complex formed between FimX_GGDEF-EAL_ and wild type or mutant PilZ, a 1:1.5 molar ratio was employed (100 μM FimX_GGDEF-EAL_ and 150 μM PilZ). Where indicated, a 2-fold excess of c-di-GMP to FimX was added. In SEC experiments studying complexes containing full-length PilB, PilZ and FimX, a Superose S6 10/300 (GE Healthcare) column was used, equilibrated in the same buffer. In this case, protein samples were mixed in equimolar ratio (30–50 μM) in a final volume of 100 μL and applied to the SEC column. Where indicated, two-fold molar excess of ATPγS and/or c-di-GMP was added to the sample.

### Multi-angle laser light scattering coupled with size-exclusion chromatography (SEC-MALS)

SEC-MALS analysis was used to determine the molar mass of PilB_1-190_, PilB_12-163_, PilB_1-190_-PilZ complex and PilB_12-163_-PilZ complex at two concentrations (3.0 mg/ml to 4.0 mg/ml) in 50 mM Tris–HCl (pH 8.0) and 50 mM NaCl, 1 mM MgCl_2_, 1 mM β-mercaptoethanol. Proteins samples (100 μL injection volume) were separated using a Superdex 200 10/300 column coupled to a miniDAWN TREOS multi-angle light scattering system and an Optilab rEX refractive index detector. For FimX, a silica-based column 7.8/300 was used (WTC-050S5, Wyatt Technology). For PilB-PilZ complex and PilB-PilZ-FimX ternary complex, a silica-based column 7.8/300 was used (WTC-015S5, Wyatt Technology). In these cases, the column was equilibrated with 50 mM Tris–HCl (pH 8.0) and 50 mM NaCl, 5mM MgCl_2_. The ligands (ATPγS and /or c-di-GMP) were added at molar ratio 1:2 protein to ligand when indicated. Data analysis was performed using the Astra Software package, version 7.1 (Wyatt Technology Corp). Molecular mass was calculated assuming a refractive index increment dn/dc = 0.185 ml/g [78].

### NMR spectroscopy

NMR sample of ^15^N-labeled PilB_12-163_ was prepared in 20 mM Tris-HCl pH 7.0, 50 mM NaCl and 1 mM MgCl_2_ at 150 μM concentration. The ^15^N-HSQC experiments were acquired in the absence and presence of unlabeled PilZ at molar ratio of 1:1.5, at 298 K on a Bruker Avance III spectrometer operating at 800 MHz (^1^H frequency) and equipped with a cryogenic TCI probe. NMR spectra were processed with NMRPipe [79] and analyzed using CCPNMR Analysis 2.4.1 [80].

### ATPase assay

The ATPase activity of PilB-PilZ complex was analyzed by malachite green assay as previously described [81]. Briefly, 1 μM of PilB-PilZ complex was used for each reaction in the absence and presence of 1 μM FimX. The assay was carried out in reaction buffer containing 25 mM Tris-HCl pH 7,0, 50 mM NaCl, 2 mM MgCl_2_, 2 mM β-mercaptoethanol and 1 mM ATP (and 20 μM c-di-GMP where indicated) and incubated for 12 h at 30°C. An aliquot (50 μL) of each reaction was transferred to a 96-well plate and the reaction was stopped by the addition of 200 μl of the phosphate assay reagent. Phosphate assay reagent was freshly prepared using 3 volumes of 0.045% malachite green hydrochloride (Sigma-Aldrich), 1 volume of ammonium molybdate (4.2% in 4 N HCl) and 1/100 volume of 1% Triton X-100. After 10 min of incubation at room temperature, the optical density was measured at 650 nm using a 96-well microplate reader (Spectramax–Molecular Devices). Reactions lacking PilB-PilZ complex or ATP were included as negative controls. The phosphate released during the reaction was measured using a standard curve of 1 to 150 μM KH_2_PO_4_.

### Fluorescence titration

The formation of the PilB_1-190_-PilZ_W69_5OHW_ and FimX_EAL_-PilZ_W69_5OHW_ complexes were monitored by changes in the 5-hydroxytryptophan fluorescence emission spectra of PilZ_W69_5OHW_ upon addition of different amounts PilB_1-190_ or FimX_EAL_. The assay was carried out in buffer containing 50 mM Tris-HCl pH 8,0, 50 mM NaCl, 1 mM MgCl_2_, 1 mM β-mercaptoethanol and the initial concentrations of PilZ_W69_5OHW_ was 1 μM. The samples were equilibrated for 2 min before each measurement. Titration experiments were performed using a RF-6000 fluorescence spectrophotometer (SHIMADZU). The excitation wavelength was 310 nm (bandwidth: 5 nm), and the emission spectra were recorded between 325–445 nm (bandwidth: 5 nm). Dissociation constants were calculated assuming a simple 1:1 bonding model, as previously described [82] using the SigmaPlot 11 software (Systat Software Inc.).

### Cloning of constructs for genomic deletions and insertions

Primers, plasmid and strains used for cloning and PCR verifications are listed in **S4 and S5 Tables**. Genomic deletion and insertion in the *X*. *citri* (strain 306) genome were introduced by two-step allelic exchange method [83] with small modifications as described [84]. Briefly, for gene deletions, two fragments of approximately 1000 bp corresponding to up- and downstream from the region of interest were amplified by PCR (Phusion polymerase, Thermo Scientific) using primers containing either homology region to the pNPTS138 vector backbone or the up or down-fragment and cloned into the pNPTS138 suicide vector by Gibson assembly (NEB). For the gene insertion of fluorescent reporter, the *msfgfp or mcherry* genes was amplified from pDHL1029 [85] and pDHL-mCherry [86], respectively, introducing a flexible N- or C-terminal liker (Ser-Gly-Gly-Gly-Gly). Separately, ~1,000 bp fragments of the upstream and downstream regions from the star (N-terminal fusion) or stop (C-terminal fusion) codon were amplified by PCR (Phusion polymerase, Thermo Scientific) from *X*. *citri* genomic DNA, using primers containing either homology to the pNPTS138 vector backbone or the *msfgfp or mcherry* gene (**S1 Table**). The three fragments were cloned into the pNPTS138 vector using Gibson Assembly (NEB). The resulting plasmid was used to transform the appropriate *X*. *citri* strain by electroporation (2.0 kV, 200 Ω, 25 μF, 0.2 cm cuvettes; Bio-Rad). A first recombination event was selected for on LB plates containing 50 μg/ml kanamycin. Transformants were streaked for single colonies on kanamycin plates after which several single colonies of the merodiploids (Kan^R^, Suc^S^) were streaked on NaCl-free LB-agar supplemented with sucrose (10 gl^−1^ tryptone, 5 gl^−1^ yeast extract, 60 gl^−1^ sucrose and 15 gl^−1^ agar), selecting for a second recombination event creating either a wild-type (reversion) or mutant allele. After confirmation of the loss of the kanamycin resistance cassette together with sacB, a PCR was performed using primers that hybridize outside of the homology regions to identify the genomic deletions or insertion and confirmed by Sanger sequencing of the PCR product.

### Twitching motility assay

Twitching motility was assayed by the stab-inoculation method in KB medium in a microscopy chamber covered with glass slides [4]. Briefly, *X*. *citri* strains were grown on LB agar (1.5% wt/vol) supplemented with the appropriate antibiotic at 28°C for 48 hours. Using a sterile toothpick, *X*. *citri* cells were collected from an isolated colony and stabbed through KB-agar (1%wt/vol) supplemented with 2 mM CaCl_2_ to the plastic surface of microscopy chamber (Nu155411; Lab-Tek, NUNC). Chambers were statically incubated in a humidified chamber at 28°C for 48 hours. Twitching zone was visualized by an inverted fluorescence microscope (Nikon Eclipse Ti-E) with specific excitation and emission filters for GFP. The images obtained were analyzed with Fiji (ImageJ) software [87]. For time-lapse experiments, overnight cultures of *X*. *citri* strains were grown in 2xTY medium at 28°C with shaking at 200 rpm. After a first overnight growth period, cells were transferred at a 100-fold dilution into 2xTY media for a second overnight growth to synchronize growth. Cells were then diluted 100-fold in fresh media and 1 μL of cell suspension was spotted on a thin slab of KB-agar (1% w/v) supplemented with 2 mM CaCl_2_, after 4- to 6-hour growth, phase contrast images were obtained with a Leica DMi-8 epifluorescent microscope. The KB-agar slabs were constructed as described [88].

### Fluorescence microscopy

Overnight cultures of *X*. *citri* strains were grown as described above. Cells were then diluted 100-fold in KB fresh media (using 0,2% casamino acids as nitrogen source) and 1 μL of cell suspension was spotted on KB-agarose (1,5% w/v, using 0,2% casamino acids as nitrogen source) supplemented with 2 mM CaCl_2_ and incubated at 30°C for 6 hours before imaging. The KB-agar slabs were constructed as described [88]. Phase contrast and msfGFP and/or mCherry emission images were obtained with a Leica DMi-8 epifluorescent microscope. Fluorescence emission of msfGFP and mCherry were captured using 2000 and 3000 ms exposure times at maximum excitation light intensities. The microscope was equipped with a DFC365 FX camera (Leica), a HC PL APO 100x/1.4 Oil ph3 objective (Leica) and excitation-emission band-pass filter cubes for GFP (Ex.: 470/40, DC: 495, Em.: 525/50; Leica) and mCherry (Ex.: 540/80, DC: 585, Em.: 592–668; Leica) foci. To determine the polar localization of foci and quantify the polar fluorescence intensities, the images were analyzed using the MicrobeJ software package [89].

### Bacteriophage plaque assay

Overnight cultures *X*. *citri* wild-type and mutant strains were grown in 2×TY medium, collected by centrifugation, and resuspended in fresh medium at an OD_600nm_ of 0.3. *X*. *citri* strains were mixed with warm liquefied KB agar (0.7% wt/vol) supplemented with 2 mM CaCl_2_ to form the top agar layer poured into petri dishes carrying previously solidified KB agar (1% wt/vol) + 2 mM CaCl_2_. Plates were dried for 5 min at room temperature and then spotted with 5 μL of dilutions (from 10^0^ to 10^10^) of the bacteriophage ΦXacm4-11 stock solution (10^12^ PFU/mL) and incubated at 28°C for 24 h as described [4].

### Western blot assay

Western blot assays were performed using total protein extract of *X*. *citri* wild type or Δ*pilZ* strains carrying the pUFR047 vector directing the expression of wld-type or mutant PilZ proteins (I10E, F49E, F49E/L51E, F49A/L51A and ΔM117). *X*. *citri* cells were grown in petri dishes on KB-agar 1% supplemented with 2 mM CaCl_2_ and 10 μg mL^-1^ of gentamicin and incubated at 28°C for 3 days. Bacteria were collected using a plastic spatula and resuspended in 15 mL of 1xPBS and the OD_600nm_ for all strains was measured and adjusted to 1.5. Bacterial cells were collected by centrifugation and the bacterial pellet washed three times with 10 mL of 1xPBS. After this, the bacterial pellet was resuspended in 400 μL of 1xPBS and 100 μL of denaturing sample buffer (5x) and incubated for 5 min at 95°C. Samples (20 μl) were resolved in 10-well Tricine–SDS–PAGE gels, transferred to nitrocellulose membrane (Bio Rad), and blocked for 12 h using 5% skimmed milk in 1×PBS. Primary antibodies produced in rabbit against PilZ (AbPilZ; 1:5000) [23] and VirB8 (AbVirB8; 1:8000) [90] were used. Secondary goat anti-rabbit IgG-IRDye 800CW (Li-Cor 32211; 1:8000) were used for AbPilZ and visualizing using a ChemiDoc MP Imaging System (Bio Rad), and secondary goat anti-rabbit IgG-AP conjugate (Bio Rad 1706518; 1:8,000) was used for and AbVirB8 with BCIP (VWR 0885) and NBT (Sigma-Aldrich N6876) for detection.

### Thermal denaturation of PilZ and mutants

The thermal denaturation of wild-type PilZ and its mutants (I10E, D46A/E47A and F49E/L51E) was accompanied by differential scanning fluorescence (DSF) as previously described [91] using a QuantStudio 3 Real-Time PCR (Thermo Fisher Scientific) instrument. Briefly, 50 μL aliquots of purified protein dissolved in 50 mM Tris-HCl pH 8.0, 50 mM NaCl, 5 mM MgCl_2_, 125x concentrated SYPRO Orange dye (Thermo Fisher Scientific) were distributed into optical 96-well plates (Life Technologies). The final protein concentrations were: PilZ_Wt_: 240 μM; PilZ_F49E/L51E_: 240 μM; PilZ_I10E_: 240 μM; and PilZ_D46A/E47A_: 60 μM. The plates were sealed with real-time compatible adhesive film (Life Technologies). Fluorescence data were collected using the built-in ROX filter while the temperature was raised from 25°C to 95°C at a rate of 3°C/minute. Data were subsequently processed using the Protein Thermal Shift software (Thermo Fisher Scientific), and the Tm values were determined based on the maxima of the first derivative of the fluorescence versus temperature plot. The reported Tm values represent the mean and standard error of three different experiments.

### Construction of homology models

Homology models were built using the I-Tasser [92] and Phyre2 [93] servers. The homology model for the *X*. *citri* PilB hexameric core (ND2 and ATPase domains, residues 202–568) was based on *G*. *metallireducens* PilB ND2-ATPase structure (PDB code 5TSH) [36]. The homology model of the cytosolic portion of the *X*. *citri* PilM-PilN complex (PilM residues 15–359 and PilN residues 1–8) was built based on the *P*. *aeruginosa* PilM-PilN structure (PDB: 5EOU) [55]. The homology model for *X*. *citri* PilC dimer (residues 74–418) was built based on the low resolution cryo-EM tomography model of *M*. *xanthus* PilC (PDB: 3JC8) [9].

## Supporting information

S1 Fig SEC-MALS analysis of PilB N-terminal fragments and their complexes with PilZ. A 0.1 ml protein sample (3.0 mg/ml– 4 mg/mL) was separated by passage through a Superdex 200 column (S200 10/300, GE) coupled to a multi-angle light scattering system and refractive index detector. Protein elution was monitored at 280 nm (continuous line). Open circles indicate the calculated molecular mass distributions. The following measured molecular weights (MW) were calculated: **A)** PilB_1-190_ = 22 kDa (20 kDa MW_theoretical_), **B)** PilB_12-163_ = 17 kDa (17 kDa MW_theoretical_), **C)** PilB_1-190_-PilZ complex = 33 kDa (33 kDa MW_theoretical_) and **D)** PilB_12-163_-PilZ complex = 30 kDa (29 kDa MW_theoretical_).
(TIF)

S2 Fig Crystal structure of the PilB_12-163_-PilZ complex. **A)** Cartoon representation of the asymmetric unit of the PilB_12-163_-PilZ crystal that contains two copies of each subunit. PilB_12-163_ chains are colored in green (chain A) and blue (chain B) and PilZ chains are colored in yellow (chain C) and magenta (chain D). **B)** Sub-domains and secondary structure elements of PilB_12-163_. *Left*: The ND0 sub-domain is shown in orange and the ND1 sub-domain is shown in green. *Right*: Topology diagram for PilB_12-163_. **C)** Surface representation of *X*. *citri* PilZ with the conserved motifs MI-MV in the PA2960/XAC1133 orthologous group colored, as described in Guzzo et al. (2009). **D)** Superposition of *X*. *citri* PilZ crystal structures. PilZ crystal structure on its own (PDB: 3CNR), PilZ within the PilZ-FimX_EAL_ complex (PDB: 4FOU) and PilZ within the PilB_12-163_-PilZ complex (this study). One important difference between the three PilZ structures is that the last 11 residues of PilZ (residues 107–117) are well structured in the PilB_12-163_-PilZ complex, but unstructured in PilZ on its own and in the FimX_EAL_-PilZ complex. **E)** 2F_0_-F_C_ electron density map (contoured at 1.0 σ) for the 1.7 Å PilB_12-163_-PilZ structure in the region around PilZ residue M117 and the hydrophobic pocket made up of conserved PilB and PilZ residues. **F)** Surface representation of the PilB_12-163_-PilZ complex with PilB_12-163_ colored in green and PilZ colored in yellow except for residues 107–117 (the conserved motif MV) in red.
(TIF)

S3 Fig Size exclusion chromatography (SEC) analysis of the interactions between PilZ and PilB_1-190_ and their mutants. Size exclusion chromatography (Superdex 200, 10/300 column) analysis of interactions of PilB_1-190_ with PilZ mutants **(A-F)** and of PilB_1-190_ mutants with PilZ **(G-L)**. In each panel, the elution profile of the PilB_1-190_-PilZ mixtures (1:1 molar ratio, black line) are shown on the *left* and SDS-PAGE analysis of representative fractions are shown on the *right*. The elution profiles for PilB_1-190_ and PilZ mutants on their own are shown in blue and red respectively. Note that, due to its partially unfolded nature, PilB_1-190_ alone elutes with a volume less than that of the PilB_1-190_-PilZ complex. PilZ mutants: **A**) F28A, **B**) Y22A, **C)** W69A, **D)** M117G, **E)** Δ107–117, **F)** ΔM117. PilB_1-190_ mutants: **G)** F77A, **H)** F101A, **I)** F108, **J)** R103A, **K)** E132A, **L)** F101/108A. Each experiment was performed at least three times and representative results are shown.
(TIF)

S4 Fig The PilZ_Δ107-117_-FimX_GGDEF-EAL_-c-di-GMP complex. A and B) Ribbon representations of the two main modes of contact observed in the crystal lattice of the PilZ_Δ107-117_-FimX_GGDEF-EAL_-c-di-GMP complex. PilZ_Δ107–117_ is colored blue and FimX_GGDEF-EAL_ colored magenta. A: PilZ-FimX interface 1. B: PilZ-FimX interface 2. No density for the FimX GGDEF domain was observed and is therefore missing from the model. C) 2F_O_-F_C_ electron density map (contoured at 1.0 σ) for the PilZ_Δ107-117_-FimX_GGDEF-EAL_-c-di-GMP structure in the region around the c-di-GMP ligand (shown in stick). D) 2F_O_-F_C_ electron density map (contoured at 1.0 σ) for inter subunit contacts at interface 1 in the PilZ_Δ107-117_-FimX_GGDEF-EAL_-c-di-GMP structure in the region around the c-di-GMP ligand (shown in stick). E) Depiction of the crystal lattice along the **a** and **b** (left), **a** and **c** (center) and **b** and **c** (right) axes. Here, the coloring scheme is the same as in A except that the first helix from the EAL domain (residues 436–454) are colored black.
(TIF)

S5 Fig Superposition of PilB_12-163_-PilZ and PilZ-FimX_EAL_ structures. **A)***Left*: Cartoon representations of the PilZ-FimX_EAL_-c-di-GMP (FimX_EAL_ colored in blue, PilZ colored in orange and c-di-GMP (stick model) colored in red) and PilB_12-163_-PilZ (PilB_12-163_ colored in green and PilZ colored in yellow) complexes. *Right*: Superposition of PilB_12-163_-PilZ and PilZ-FimX_EAL_ complexes using PilZ as reference. **B)** Structural alignment of the PilZ structures from **A** showing the common interface residues in both complexes as sticks. Note that in this figure, the interaction interface (interface 1) between FimX_EAL_ and PilZ is as described previously[24]. An alternative mode of interaction (interface 2) is proposed and tested as described in the main text and detailed in **Figs S6, 2F and 4**.
(TIF)

S6 Fig Size exclusion chromatography of PilZ-FimX_GGDEF-EAL_ and PilB_1-190_-PilZ binary complexes. Size exclusion chromatography (Superdex 200, 10/300 column) analysis of interactions of PilZ mutants (PilZ_F49E/L51E_, PilZ_I10E_ and PilZ_D46A/E47A_) with FimX_GGDEF-EAL_ **(A)** and PilB_1-190_ **(B)**. In each chromatogram, the elution profile of the PilZ–FimX_GGDEF-EAL_ (1.5:1molar ratio, black line) (**A**) and PilB_1-190_ –PilZ (1:1 molar ratio, black line) mixtures (**B**) are shown on the *left* and the SDS-PAGE analysis of representative fractions are shown on the *right*. Where indicated, c-di-GMP was added to the PilZ–FimX_GGDEF-EAL_ mixture (2-fold excess of c-di-GMP to FimX_GGDEF-EAL_ (continuous red line in **A**)). Note that the addition of c-di-GMP to the PilZ_wt_ (wild type PilZ)–FimX_GGDEF-EAL_ and PilZ_D46A/E47A_ –FimX_GGDEF-EAL_ mixture results in a shift in its elution profile. The elution profiles for FimX_GGDEF-EAL_ alone is shown in blue in **A**. The elution profiles for PilB_1-190_ and PilZ mutants on their own are shown in blue and red respectively in **B**. In these experiments, FimX_GGDEF-EAL_ and PilB_1-190_ have N-terminal 6xHis-tags. Each experiment was performed at least three times and representative results are shown.
(TIF)

S7 Fig Evaluation of PilZ stability by thermal denaturation accompanied by differential scanning fluorescence (DSF). **A)** Calculated thermal melting temperatures (T_m_) values for PilZ_Wt_ (240 μM), PilZ_F49E/L51E_ (240 μM), PilZ_I10E_ (240 μM), and PilZ_D46A/E47A_ (60 μM). T_m_ values are reported as the mean and standard error derived from three different experiments. **B)** Normalized fluorescence vs temperature (upper panel) and normalized first derivative of fluorescence vs temperature (lower panel). PilZ_Wt_ (solid line), PilZ_F49E/L51E_ (long dashed line), PilZ_I10E_ (short dashed line), and PilZ_D46A/E47A_ (dotted line).
(TIF)

S8 Fig PilB_12-163_-PilZ and PilB_1-190_-PilZ can form ternary complexes with FimX_EAL_ and FimX_GGDEF-EAL_ fragments. Size exclusion chromatography (Superdex 200 resin, 10/300 column) analysis of the interactions of PilB_12-163_-PilZ and PilB_1-190_-PilZ complexes with different FimX fragments. Chromatograms are shown for the PilB_12-163_-PilZ or PilB_1-190_-PilZ complexes on their own (blue), FimX fragments on their own (green) and 1:1 mixtures PilB-PilZ and FimX fragments (100 μM) in the absence (red) or presence (black) of c-di-GMP (2-fold excess of c-di-GMP to FimX). (**A**) PilB_12-163_-PilZ complex and FimX_EAL_. (**B**) PilB_1-190_-PilZ complex and FimX_EAL_. (**C**) PilB_12-163_-PilZ complex and FimX_GGDEF-EAL_. (**D**) PilB_1-190_-PilZ complex and FimX_GGDEF-EAL_. (**E**) PilB_12-163_-PilZ complex and FimX_PAS-GGDEF-EAL_. (**F**) PilB_12-163_-PilZ complex and full-length FimX. SDS-PAGE analysis of representative fractions is shown on the right of each panel (vertical dotted lines in D, E and F indicate parts of the gel removed between molecular mass markers and protein samples). Ternary complexes are observed when using FimX_EAL_ and FimX_GGDEF-EAL_ but not when using FimX_PAS-GGDEF-EAL_ or full-length FimX. Each experiment was performed at least three times and representative results are shown.
(TIF)

S9 Fig SEC analysis of ternary complexes containing wild-type and mutant PilB. **A)** SEC-MALS analysis for PilB-PilZ complex and full-length FimX mixture in absence (continuous black line) and presence (continuous red line) of ATPγS and c-di-GMP. The red and black open circles show the calculated molecular mass distributions. In this experiment, a silica-based 7.8/300 column was used (WTC-050S5, Wyatt Technology). **B)** *Above*: SEC analysis of the PilB_K343A/E407A_-PilZ-FimX complex in the absence (continuous black line) and presence (continuous red line) of ATPγS and c-di-GMP. The elution profiles for the PilB_K343A/E407A_-PilZ complex (blue broken line) and FimX (green broken line) are also shown. *Below*: SDS-PAGE analysis of the relevant fractions eluted during SEC of the PilB_K343A/E407A_-PilZ-FimX complex +/- ATPγS/c-di-GMP. In this experiment, a Superose 6 column (10/300) column was used.
(TIF)

S10 Fig The fluorescence intensity profiles for *X*. *citri* cells expressing msfGFP-FimX, msfGFP-PilZ, msfGFP-PilB and PilQ-msfGFP. **A)** The fluorescence intensity profiles of 100 individual *X*. *citri* cells expressing msfGFP-FimX, msfGFP-PilZ, msfGFP-PilB or PilQ-msfGFP during growth on KB-agarose (1.5% w, using 0.2% casamino acids as nitrogen source) supplemented with 2 mM CaCl_2_. Cell lengths were normalized. **B)** *Left*: Fluorescence microscopy images of *X*. *citri* cells expressing mCherry-FimX or PilQ-msfGFP when grown in liquid culture (0h) or after 6 h growth (6h) on KB-agarose (1.5% w, using 0.2% casamino acids as nitrogen source) supplemented with 2 mM CaCl_2_. *Right*: Graphical representation of the fluorescence intensity profile over the length of *X*. *citri* cells expressing mCherry-FimX or PilQ-msfGFP. Note that mCherry-FimX foci are observed when grown on agarose (conditions leading to twitching) but not in liquid culture. On the other hand, PilQ-msfGFP foci are observed under both conditions with bipolar localization more common during growth in liquid media.
(TIF)

S11 Fig Type IV pilus-dependent phenotypes are maintained in *X*. *citri* strains carrying fluorescent chimeras. **A)** Time-lapse of in *X*. *citri* msfGFP-FimX, msfGFP-PilZ, msfGFP-PilB and PilQ-msfGFP strains exhibiting twitching motility. The time lapse interval (h) is indicated for each frame. Images were taken by using a phase-contrast microscope. Scale bar, 20 μm. Also see **S1–S4 Movies**. **B)** Phage ΦXacm4-11 infection assays for *X*. *citri* msfGFP-FimX, msfGFP-PilZ, msfGFP-PilB, PilQ-msfGFP, mCherry-FimX/msfGFP-PilZ, mCherry-FimX/PilQ-msfGFP and mCherry-FimX/msfGFP-PilB strains. Dark plaques are indicative of phage-induced bacterial lysis in a confluent culture background.
(TIF)

S12 Fig Structural and sequence alignment of PilB N-terminal domain homologs. **A)** Structural and **B)** sequence alignment of the N-terminal regions of *X*. *citri* PilB (green) with homologs from the Mannose-Sensitive Haemagglutinin Type IV Pilus of *V*. *cholerae* (MshE, PDB: 5HTL, light red) and the Type II secretion systems of *X*. *campestris* (XpsE, PDB: 2D28, light blue), *Vibrio vulnificus* (GspE, PDB: 4PHT, orange) and *Vibrio cholerae* (EpsE, PDB: 2BH1, yellow). The structural alignment in **A** shows both side and top views. The secondary structure elements observed in the crystal structure of *X*. *citri* PilB_12-163_ are indicated above of the sequence alignment in **B**. **C)** Summary of the structural and sequence alignment statistics described in **A** and **B**.
(TIF)

S13 Fig Superposition of the *X*. *citri* PilB_12-163_-PilZ complex with GspE-GspL_cyto_ and EpsE_Nt_-EpsL_cyto_. Superposition of the *X*. *citri* PilB_12-163_-PilZ complex (colored in green for PilB and yellow for PilZ) with (**A**) GspE-GspL_cyto_ (PDB: 4PHT, colored in orange for GspE and blue for GspL_cyto_) and (**B**) EpsE_Nt_-EpsL_cyto_ (PDB: 2BH1, colored in magenta for EpsE_Nt_ and light blue for EpsL_cyto_). The ND1 sub-domain of *X*. *citri* PilB and de N-terminal domains of GspE and EpsE were used as reference in the alignments.
(TIF)

S14 Fig Western blot analysis of heterologous PilZ expression in *Xanthomonas citri*. Western blot assays using polyclonal antibodies (Ab) against PilZ (*above*) and VirB8 (*below*). The first lane contains total extract from wild type *X*. *citri* strain containing the pUFR047-PilZ_WT_ vector. The following lanes contain total extracts from *X*. *citri* Δ*pilZ* cells carrying the empty pUFR047 vector and the vector directing the expression of PilZ_Wt_, PilZ_I10E_, PilZ_F49E_, PilZ_F49E/L51E,_ PilZ_F49A/L51A_, PilZ_ΔM117_. The same amounts of total protein were loaded on the gel. Detection of the *X*. *citri* VirB8 protein (XAC2621) was used as a control. Experiments were repeated three times with similar results.
(TIF)

S1 Table Data collection and refinement statistics of the PilB_12-163_-PilZ and PilZ_Δ107-117_-FimX_GGDEF-EAL_-c-di-GMP crystal structures. (DOCX)

S2 Table Interface residues in the PilB_12-163_-PilZ complex. (DOCX)

S3 Table Selected bacterial species in the KEGG database that code for homologs of *X*. *citri* PilZ, FimX, PilB and PilM. (DOCX)

S4 Table Cellular strains used in this study. (DOCX)

S5 Table Oligonucleotides and plasmids used in this study. (DOCX)

S6 Table Conditions for protein expression. (DOCX)

S1 Movie Bright field time-lapse microscopy movie showing actively twitching *X*. *citri* cells expressing msfGFP-FimX. Scalebar: 10μm. Time stamp (hh:mm) at top left of the movie. See Materials and Methods for growth conditions.
(AVI)

S2 Movie Bright field time-lapse microscopy movie showing actively twitching *X*. *citri* cells expressing msfGFP-PilZ. Scalebar: 10μm. Time stamp (hh:mm) at top left of the movie. See Materials and Methods for growth conditions.
(AVI)

S3 Movie Bright field time-lapse microscopy movie showing actively twitching *X*. *citri* cells expressing msfGFP-PilB. Scalebar: 10μm. Time stamp (hh:mm) at top left of the movie. See Materials and Methods for growth conditions.
(AVI)

S4 Movie Bright field time-lapse microscopy movie showing actively twitching *X*. *citri* cells expressing PilQ-msfGFP. Scalebar: 10μm. Time stamp (hh:mm) at top left of the movie. See Materials and Methods for growth conditions.
(AVI)

S5 Movie Time-lapse fluorescence microscopy movie showing actively twitching *X*. *citri* cells expressing msfGFP-FimX. Scalebar: 5μm. Time stamp (hh:mm) at top left of the movie. See Materials and Methods for growth conditions.
(AVI)

S6 Movie Time-lapse fluorescence microscopy movie showing actively twitching *X*. *citri* cells expressing msfGFP-PilZ. Scalebar: 5μm. Time stamp (hh:mm) at top left of the movie. See Materials and Methods for growth conditions.
(AVI)

S7 Movie Time-lapse fluorescence microscopy movie showing actively twitching *X*. *citri* cells expressing msfGFP-PilB. Scalebar: 5μm. Time stamp (hh:mm) at top left of the movie. See Materials and Methods for growth conditions.
(AVI)

S8 Movie Time-lapse fluorescence microscopy movie showing actively twitching *X*. *citri* cells expressing PilQ-msfGFP. Scalebar: 5μm. Time stamp (hh:mm) at top left of the movie. See Materials and Methods for growth conditions.
(AVI)
