# Supplementary figures and images for: The PilB-PilZ-FimX regulatory complex of the Type IV pilus from Xanthomonas citri
Source: PLoS Pathog. 2021 Aug 16;17(8):e1009808. doi: 10.1371/journal.ppat.1009808 (PMC8389850; doi:10.1371/journal.ppat.1009808)

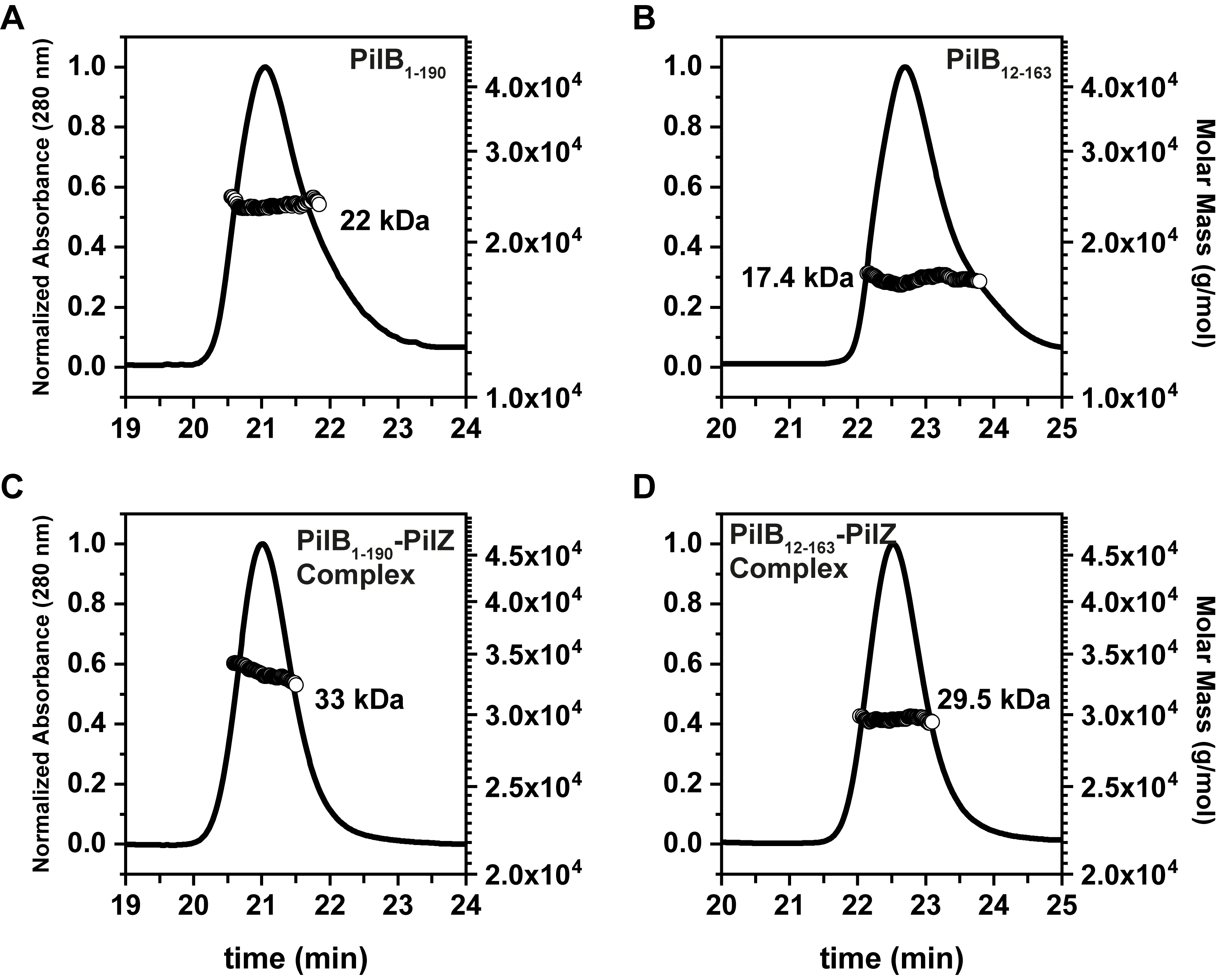

Supplement: S1 Fig — A 0.1 ml protein sample (3.0 mg/ml– 4 mg/mL) was separated by passage through a Superdex 200 column (S200 10/300, GE) coupled to a multi-angle light scattering system and refractive index detector. Protein elution was monitored at 280 nm (continuous line). Open circles indicate the calculated molecular mass distributions. The following measured molecular weights (MW) were calculated: A) PilB1-190 = 22 kDa (20 kDa MWtheoretical), B) PilB12-163 = 17 kDa (17 kDa MWtheoretical), C) PilB1-190-PilZ complex = 33 kDa (33 kDa MWtheoretical) and D) PilB12-163-PilZ complex = 30 kDa (29 kDa MWtheoretical). (TIF) [file ppat.1009808.s001.tif]

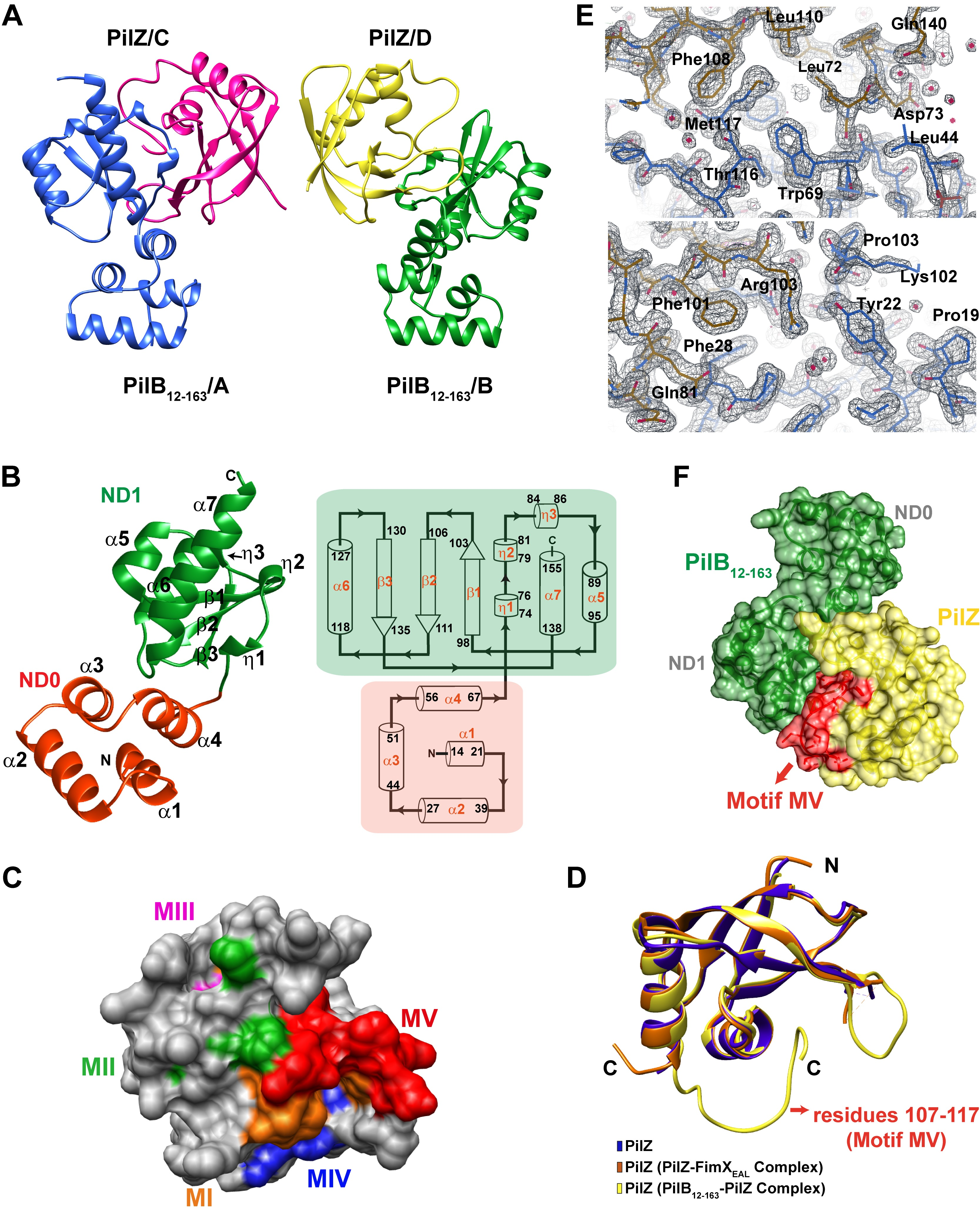

Supplement: S2 Fig — A) Cartoon representation of the asymmetric unit of the PilB12-163-PilZ crystal that contains two copies of each subunit. PilB12-163 chains are colored in green (chain A) and blue (chain B) and PilZ chains are colored in yellow (chain C) and magenta (chain D). B) Sub-domains and secondary structure elements of PilB12-163. Left: The ND0 sub-domain is shown in orange and the ND1 sub-domain is shown in green. Right: Topology diagram for PilB12-163. C) Surface representation of X. citri PilZ with the conserved motifs MI-MV in the PA2960/XAC1133 orthologous group colored, as described in Guzzo et al. (2009). D) Superposition of X. citri PilZ crystal structures. PilZ crystal structure on its own (PDB: 3CNR), PilZ within the PilZ-FimXEAL complex (PDB: 4FOU) and PilZ within the PilB12-163-PilZ complex (this study). One important difference between the three PilZ structures is that the last 11 residues of PilZ (residues 107–117) are well structured in the PilB12-163-PilZ complex, but unstructured in PilZ on its own and in the FimXEAL-PilZ complex. E) 2F0-FC electron density map (contoured at 1.0 σ) for the 1.7 Å PilB12-163-PilZ structure in the region around PilZ residue M117 and the hydrophobic pocket made up of conserved PilB and PilZ residues. F) Surface representation of the PilB12-163-PilZ complex with PilB12-163 colored in green and PilZ colored in yellow except for residues 107–117 (the conserved motif MV) in red. (TIF) [file ppat.1009808.s002.tif]

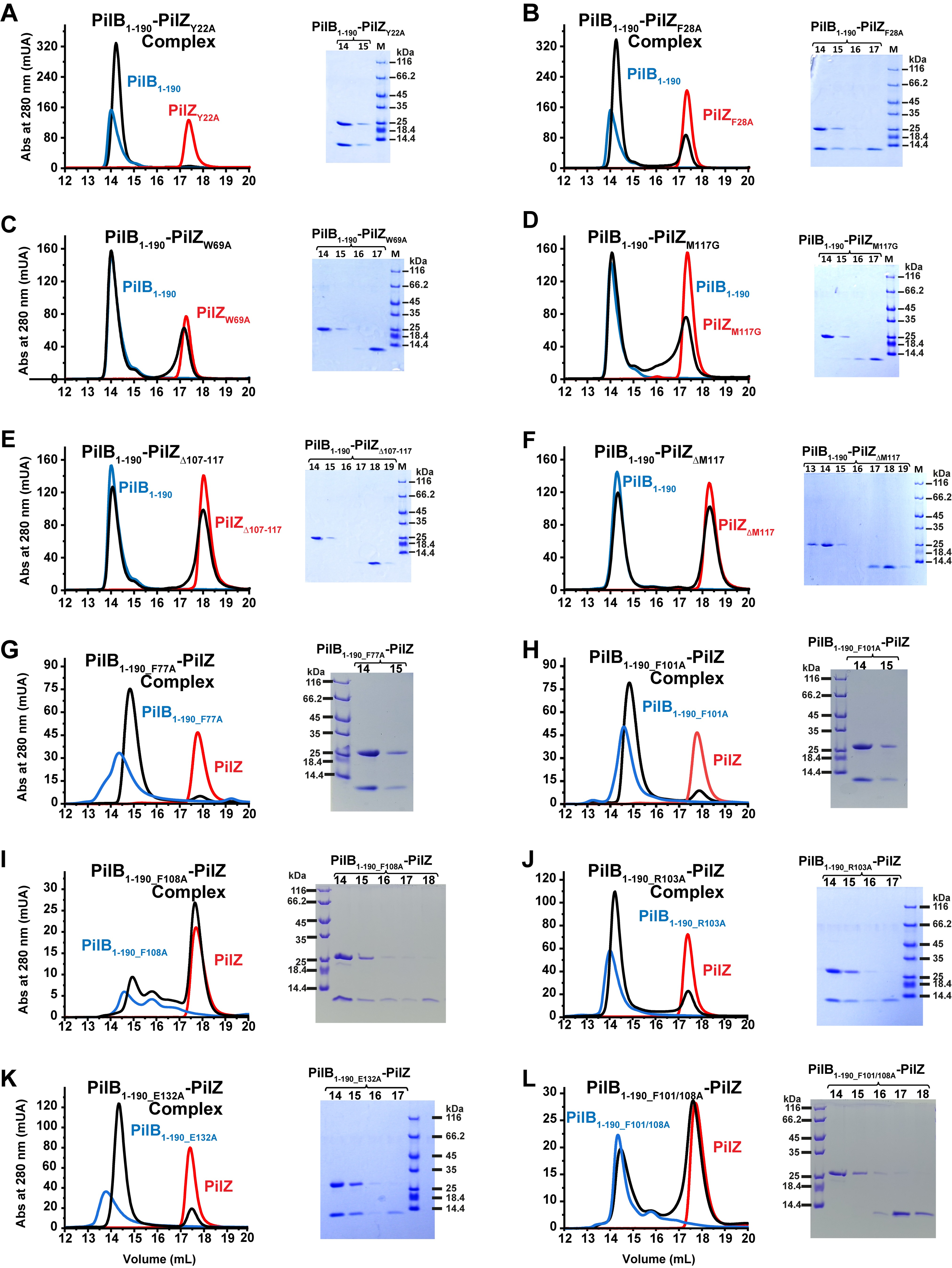

Supplement: S3 Fig — Size exclusion chromatography (Superdex 200, 10/300 column) analysis of interactions of PilB1-190 with PilZ mutants (A-F) and of PilB1-190 mutants with PilZ (G-L). In each panel, the elution profile of the PilB1-190-PilZ mixtures (1:1 molar ratio, black line) are shown on the left and SDS-PAGE analysis of representative fractions are shown on the right. The elution profiles for PilB1-190 and PilZ mutants on their own are shown in blue and red respectively. Note that, due to its partially unfolded nature, PilB1-190 alone elutes with a volume less than that of the PilB1-190-PilZ complex. PilZ mutants: A) F28A, B) Y22A, C) W69A, D) M117G, E) Δ107–117, F) ΔM117. PilB1-190 mutants: G) F77A, H) F101A, I) F108, J) R103A, K) E132A, L) F101/108A. Each experiment was performed at least three times and representative results are shown. (TIF) [file ppat.1009808.s003.tif]

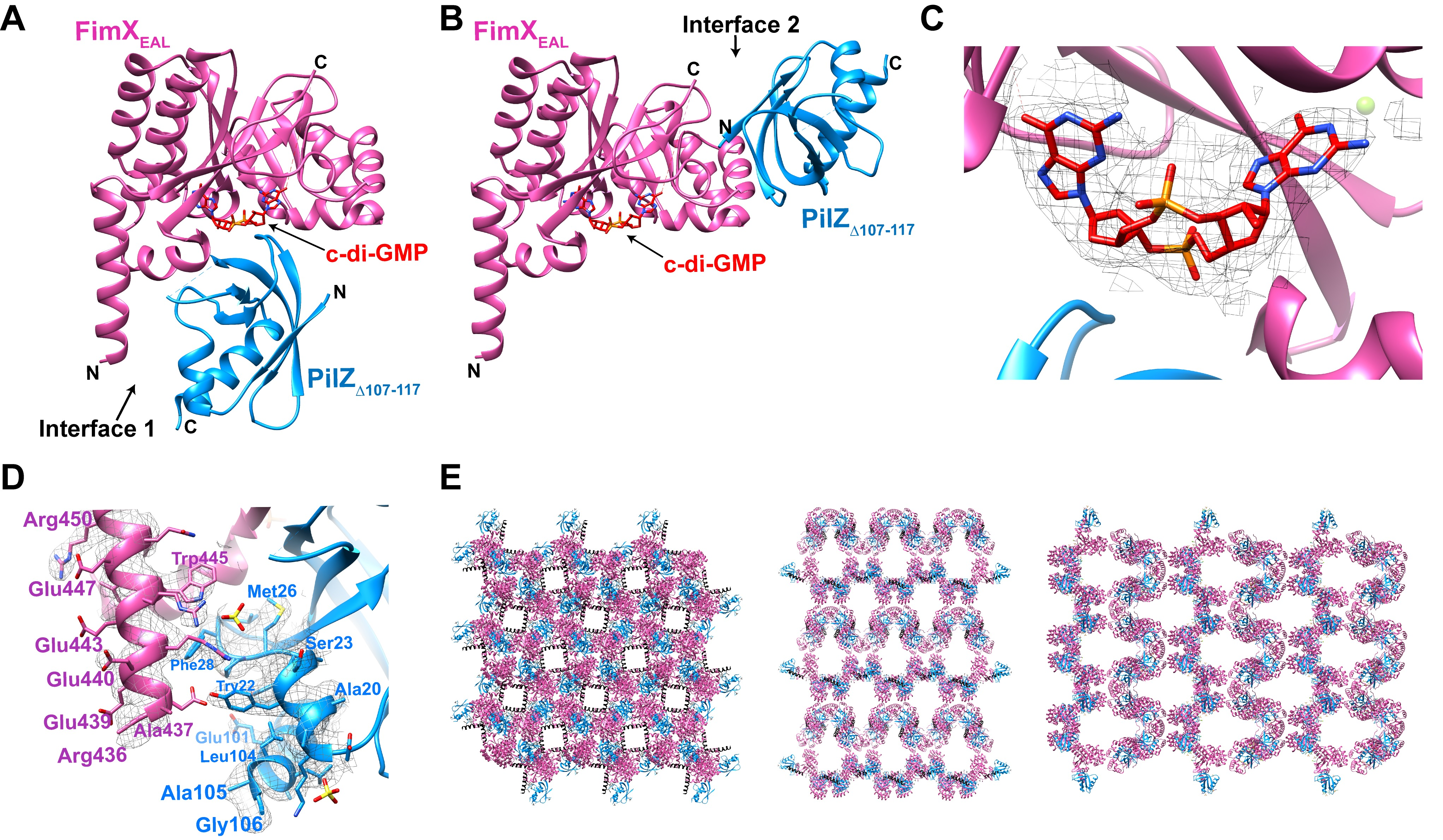

Supplement: S4 Fig — A and B) Ribbon representations of the two main modes of contact observed in the crystal lattice of the PilZΔ107-117-FimXGGDEF-EAL-c-di-GMP complex. PilZΔ107–117 is colored blue and FimXGGDEF-EAL colored magenta. A: PilZ-FimX interface 1. B: PilZ-FimX interface 2. No density for the FimX GGDEF domain was observed and is therefore missing from the model. C) 2FO-FC electron density map (contoured at 1.0 σ) for the PilZΔ107-117-FimXGGDEF-EAL-c-di-GMP structure in the region around the c-di-GMP ligand (shown in stick). D) 2FO-FC electron density map (contoured at 1.0 σ) for inter subunit contacts at interface 1 in the PilZΔ107-117-FimXGGDEF-EAL-c-di-GMP structure in the region around the c-di-GMP ligand (shown in stick). E) Depiction of the crystal lattice along the a and b (left), a and c (center) and b and c (right) axes. Here, the coloring scheme is the same as in A except that the first helix from the EAL domain (residues 436–454) are colored black. (TIF) [file ppat.1009808.s004.tif]

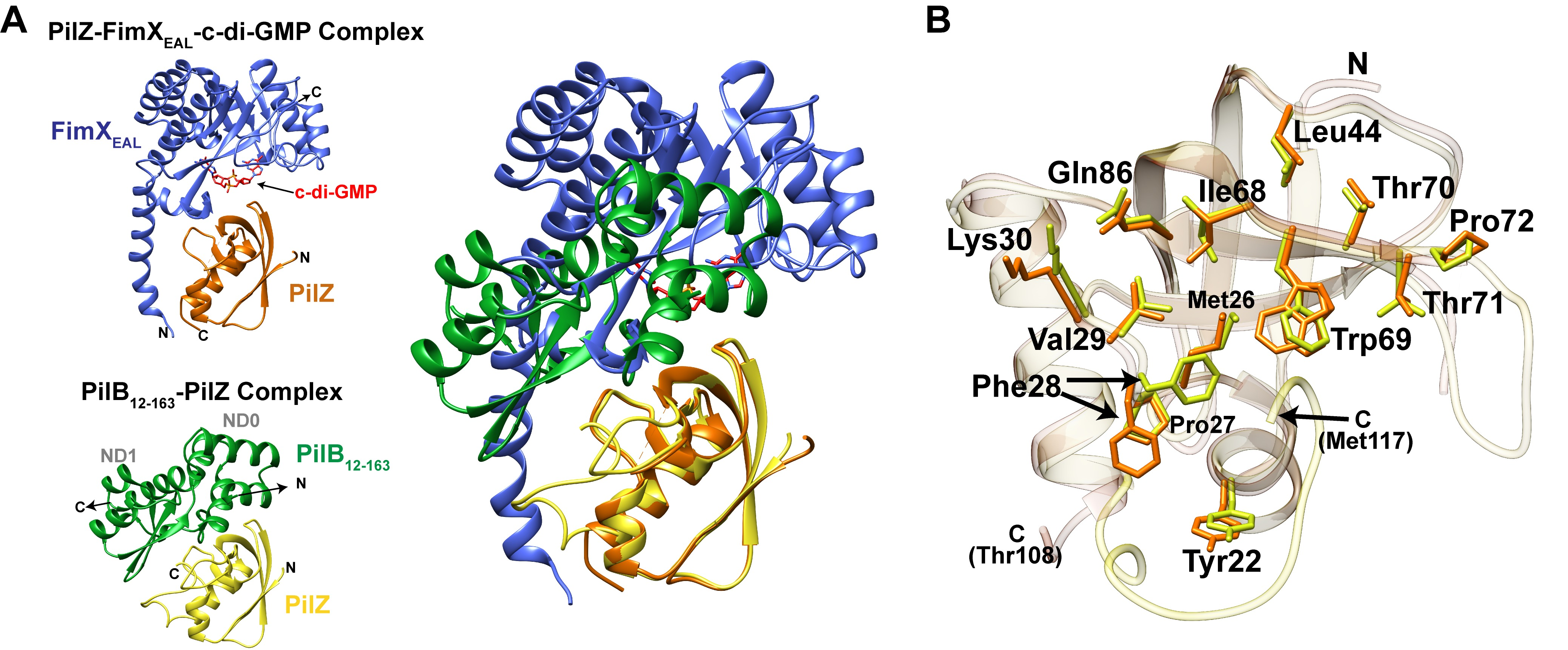

Supplement: S5 Fig — A)Left: Cartoon representations of the PilZ-FimXEAL-c-di-GMP (FimXEAL colored in blue, PilZ colored in orange and c-di-GMP (stick model) colored in red) and PilB12-163-PilZ (PilB12-163 colored in green and PilZ colored in yellow) complexes. Right: Superposition of PilB12-163-PilZ and PilZ-FimXEAL complexes using PilZ as reference. B) Structural alignment of the PilZ structures from A showing the common interface residues in both complexes as sticks. Note that in this figure, the interaction interface (interface 1) between FimXEAL and PilZ is as described previously[24]. An alternative mode of interaction (interface 2) is proposed and tested as described in the main text and detailed in Figs S6, 2F and 4. (TIF) [file ppat.1009808.s005.tif]

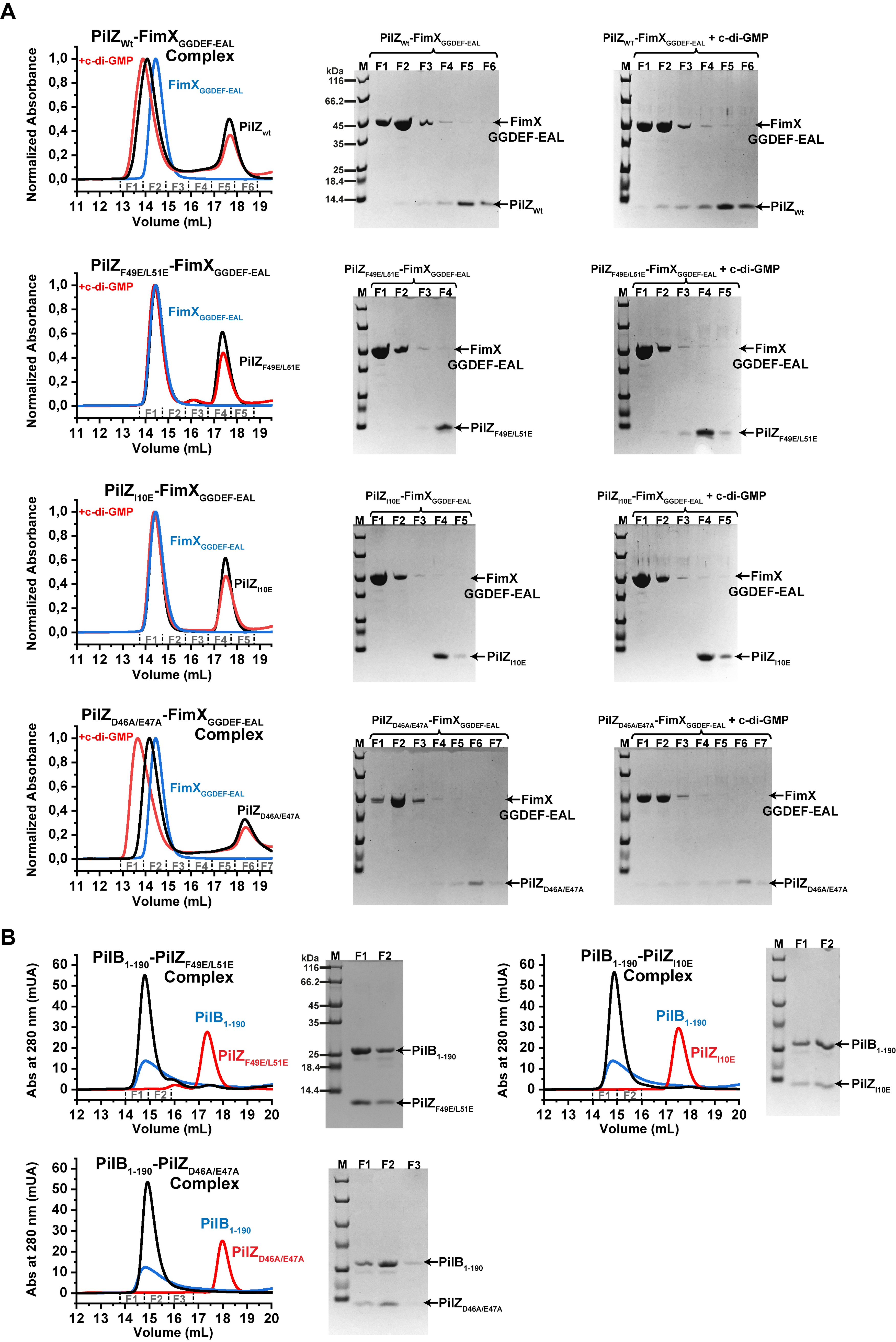

Supplement: S6 Fig — Size exclusion chromatography (Superdex 200, 10/300 column) analysis of interactions of PilZ mutants (PilZF49E/L51E, PilZI10E and PilZD46A/E47A) with FimXGGDEF-EAL (A) and PilB1-190 (B). In each chromatogram, the elution profile of the PilZ–FimXGGDEF-EAL (1.5:1molar ratio, black line) (A) and PilB1-190 –PilZ (1:1 molar ratio, black line) mixtures (B) are shown on the left and the SDS-PAGE analysis of representative fractions are shown on the right. Where indicated, c-di-GMP was added to the PilZ–FimXGGDEF-EAL mixture (2-fold excess of c-di-GMP to FimXGGDEF-EAL (continuous red line in A)). Note that the addition of c-di-GMP to the PilZwt (wild type PilZ)–FimXGGDEF-EAL and PilZD46A/E47A –FimXGGDEF-EAL mixture results in a shift in its elution profile. The elution profiles for FimXGGDEF-EAL alone is shown in blue in A. The elution profiles for PilB1-190 and PilZ mutants on their own are shown in blue and red respectively in B. In these experiments, FimXGGDEF-EAL and PilB1-190 have N-terminal 6xHis-tags. Each experiment was performed at least three times and representative results are shown. (TIF) [file ppat.1009808.s006.tif]

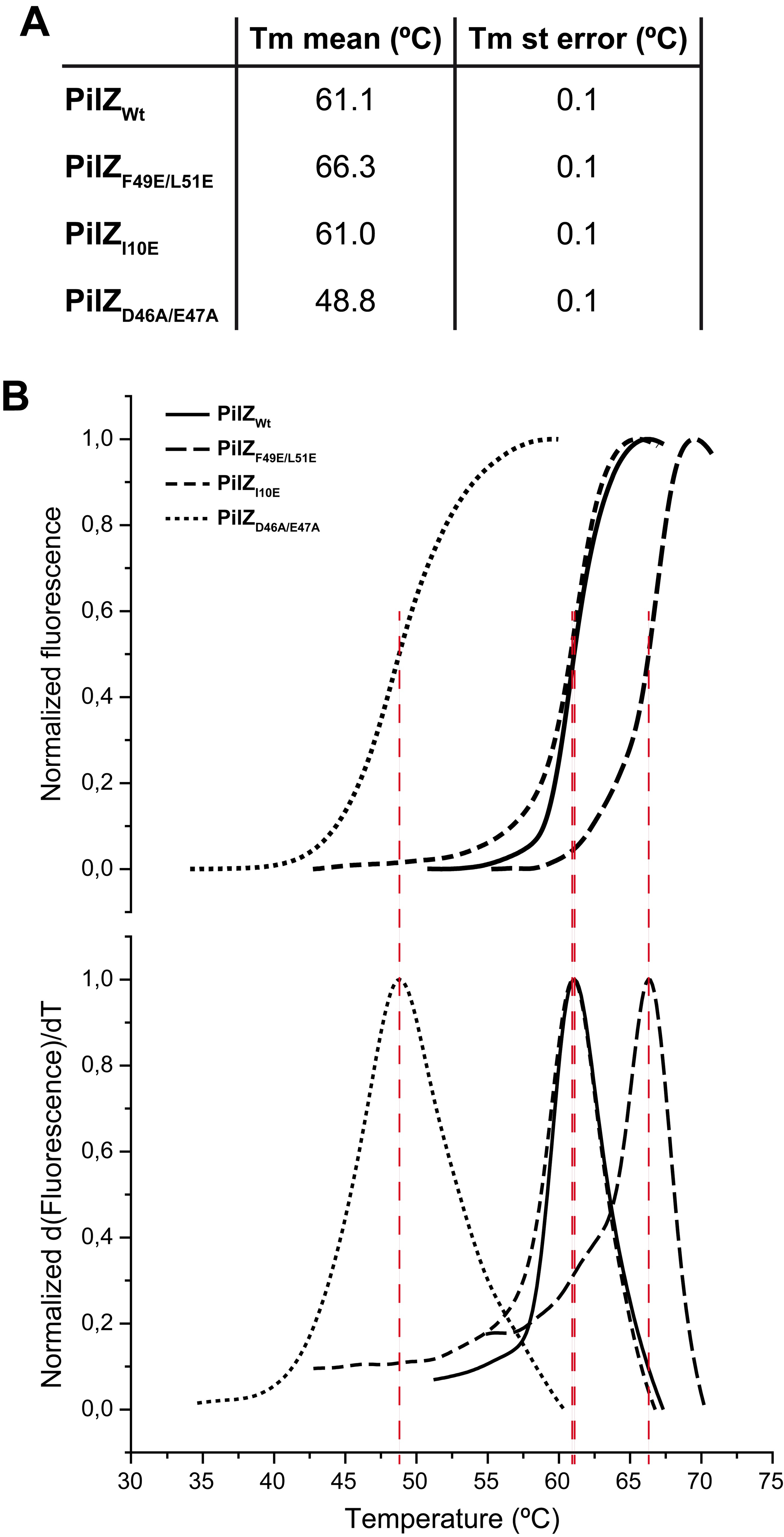

Supplement: S7 Fig — A) Calculated thermal melting temperatures (Tm) values for PilZWt (240 μM), PilZF49E/L51E (240 μM), PilZI10E (240 μM), and PilZD46A/E47A (60 μM). Tm values are reported as the mean and standard error derived from three different experiments. B) Normalized fluorescence vs temperature (upper panel) and normalized first derivative of fluorescence vs temperature (lower panel). PilZWt (solid line), PilZF49E/L51E (long dashed line), PilZI10E (short dashed line), and PilZD46A/E47A (dotted line). (TIF) [file ppat.1009808.s007.tif]

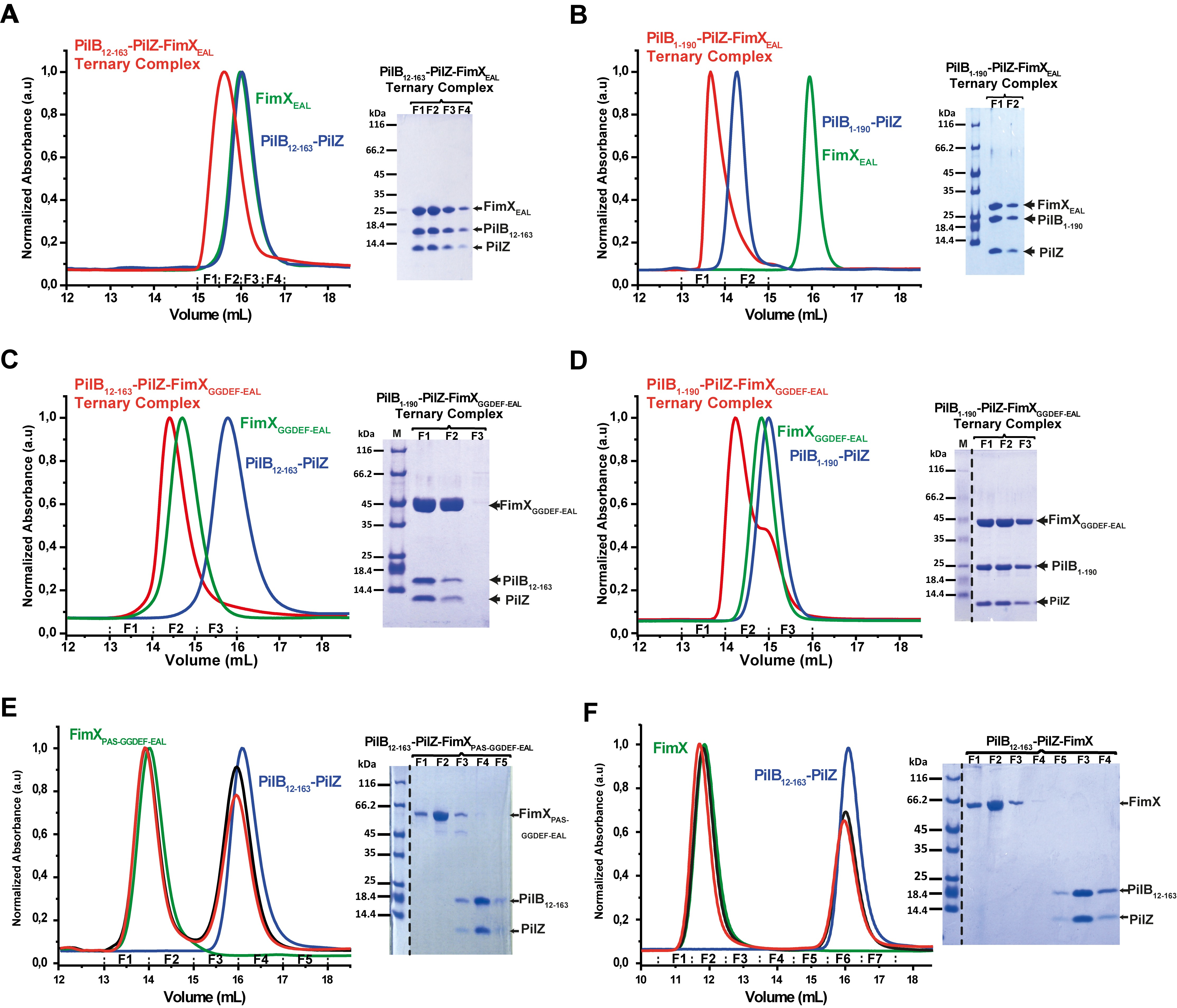

Supplement: S8 Fig — Size exclusion chromatography (Superdex 200 resin, 10/300 column) analysis of the interactions of PilB12-163-PilZ and PilB1-190-PilZ complexes with different FimX fragments. Chromatograms are shown for the PilB12-163-PilZ or PilB1-190-PilZ complexes on their own (blue), FimX fragments on their own (green) and 1:1 mixtures PilB-PilZ and FimX fragments (100 μM) in the absence (red) or presence (black) of c-di-GMP (2-fold excess of c-di-GMP to FimX). (A) PilB12-163-PilZ complex and FimXEAL. (B) PilB1-190-PilZ complex and FimXEAL. (C) PilB12-163-PilZ complex and FimXGGDEF-EAL. (D) PilB1-190-PilZ complex and FimXGGDEF-EAL. (E) PilB12-163-PilZ complex and FimXPAS-GGDEF-EAL. (F) PilB12-163-PilZ complex and full-length FimX. SDS-PAGE analysis of representative fractions is shown on the right of each panel (vertical dotted lines in D, E and F indicate parts of the gel removed between molecular mass markers and protein samples). Ternary complexes are observed when using FimXEAL and FimXGGDEF-EAL but not when using FimXPAS-GGDEF-EAL or full-length FimX. Each experiment was performed at least three times and representative results are shown. (TIF) [file ppat.1009808.s008.tif]

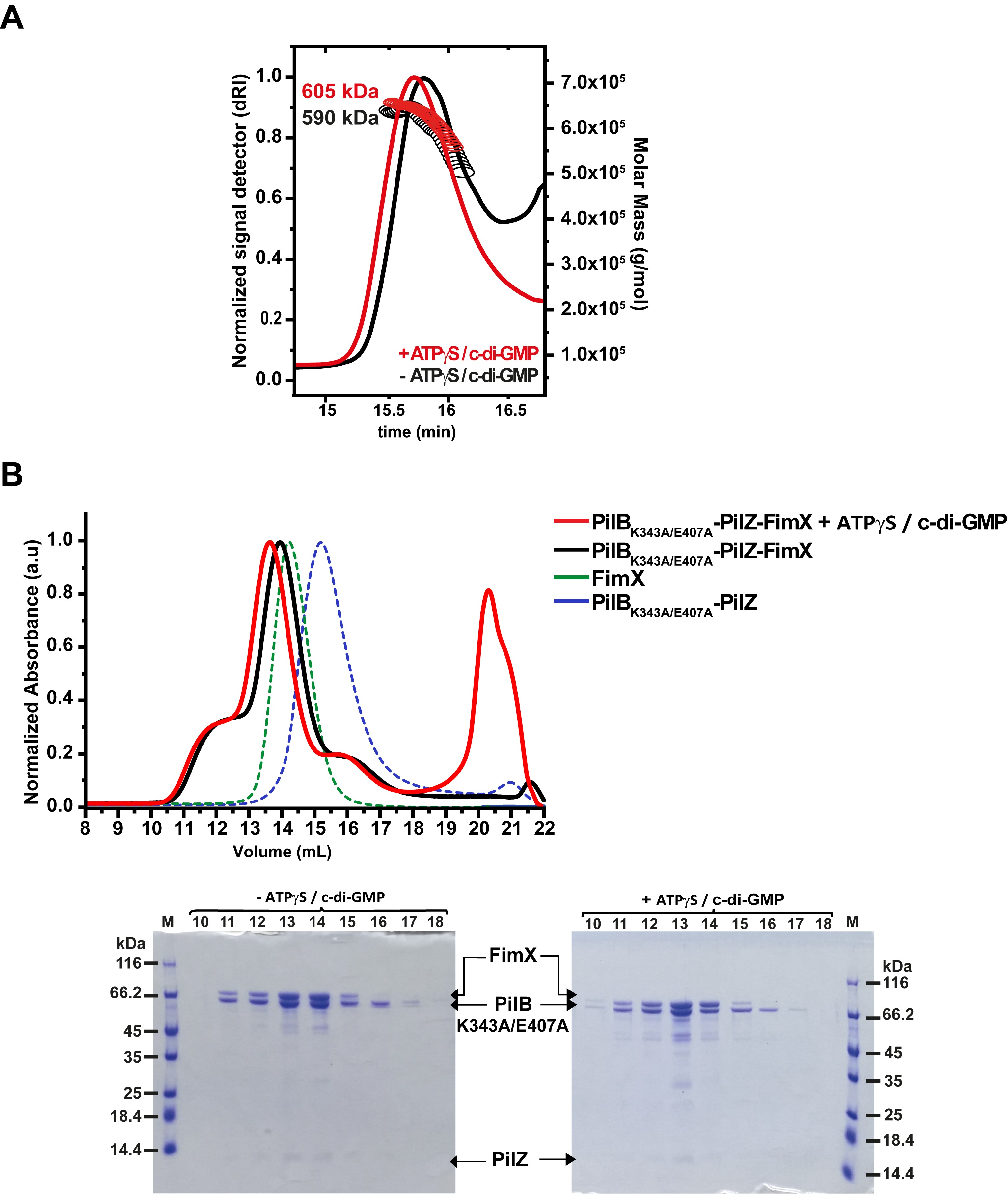

Supplement: S9 Fig — A) SEC-MALS analysis for PilB-PilZ complex and full-length FimX mixture in absence (continuous black line) and presence (continuous red line) of ATPγS and c-di-GMP. The red and black open circles show the calculated molecular mass distributions. In this experiment, a silica-based 7.8/300 column was used (WTC-050S5, Wyatt Technology). B) Above: SEC analysis of the PilBK343A/E407A-PilZ-FimX complex in the absence (continuous black line) and presence (continuous red line) of ATPγS and c-di-GMP. The elution profiles for the PilBK343A/E407A-PilZ complex (blue broken line) and FimX (green broken line) are also shown. Below: SDS-PAGE analysis of the relevant fractions eluted during SEC of the PilBK343A/E407A-PilZ-FimX complex +/- ATPγS/c-di-GMP. In this experiment, a Superose 6 column (10/300) column was used. (TIF) [file ppat.1009808.s009.tif]

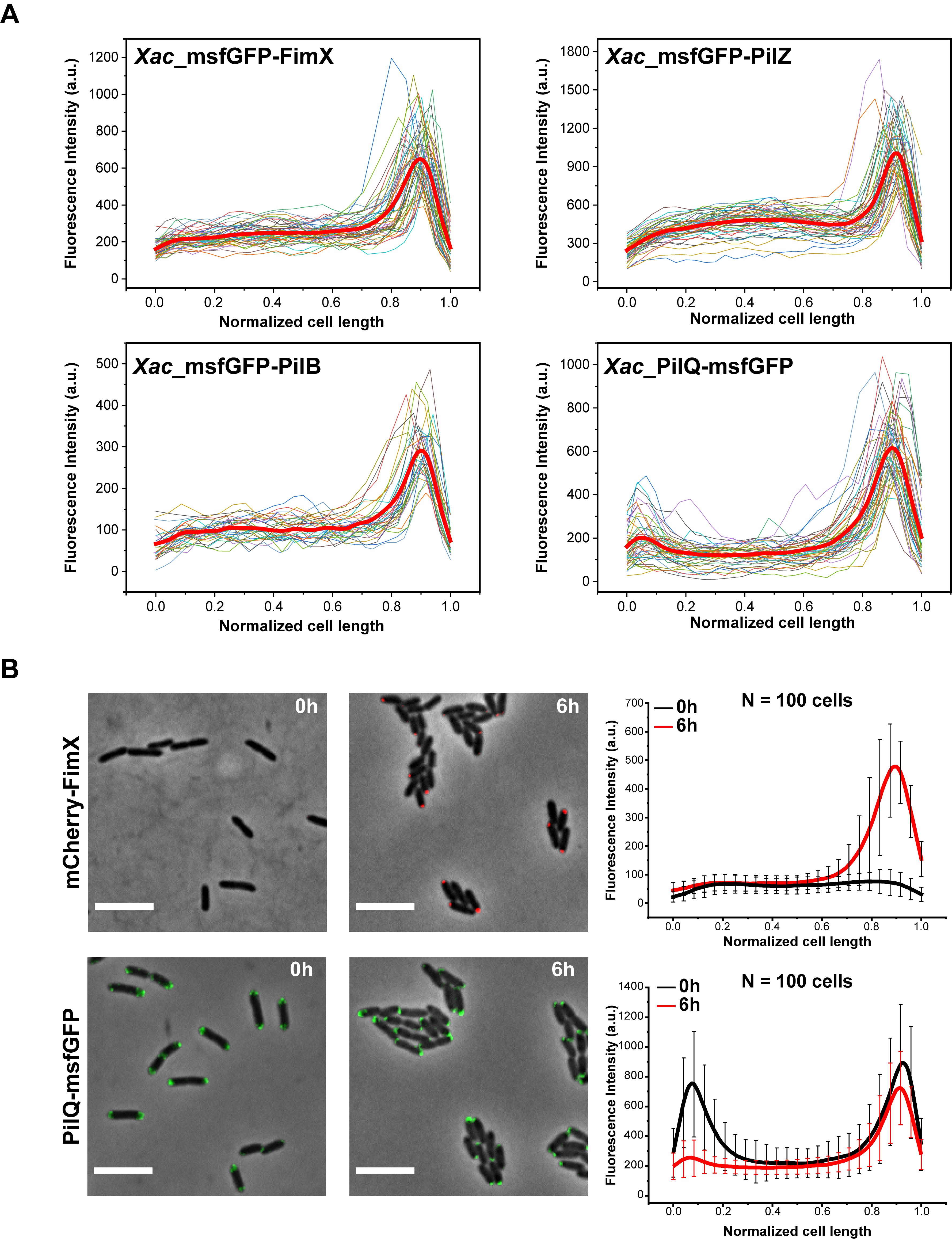

Supplement: S10 Fig — A) The fluorescence intensity profiles of 100 individual X. citri cells expressing msfGFP-FimX, msfGFP-PilZ, msfGFP-PilB or PilQ-msfGFP during growth on KB-agarose (1.5% w, using 0.2% casamino acids as nitrogen source) supplemented with 2 mM CaCl2. Cell lengths were normalized. B) Left: Fluorescence microscopy images of X. citri cells expressing mCherry-FimX or PilQ-msfGFP when grown in liquid culture (0h) or after 6 h growth (6h) on KB-agarose (1.5% w, using 0.2% casamino acids as nitrogen source) supplemented with 2 mM CaCl2. Right: Graphical representation of the fluorescence intensity profile over the length of X. citri cells expressing mCherry-FimX or PilQ-msfGFP. Note that mCherry-FimX foci are observed when grown on agarose (conditions leading to twitching) but not in liquid culture. On the other hand, PilQ-msfGFP foci are observed under both conditions with bipolar localization more common during growth in liquid media. (TIF) [file ppat.1009808.s010.tif]

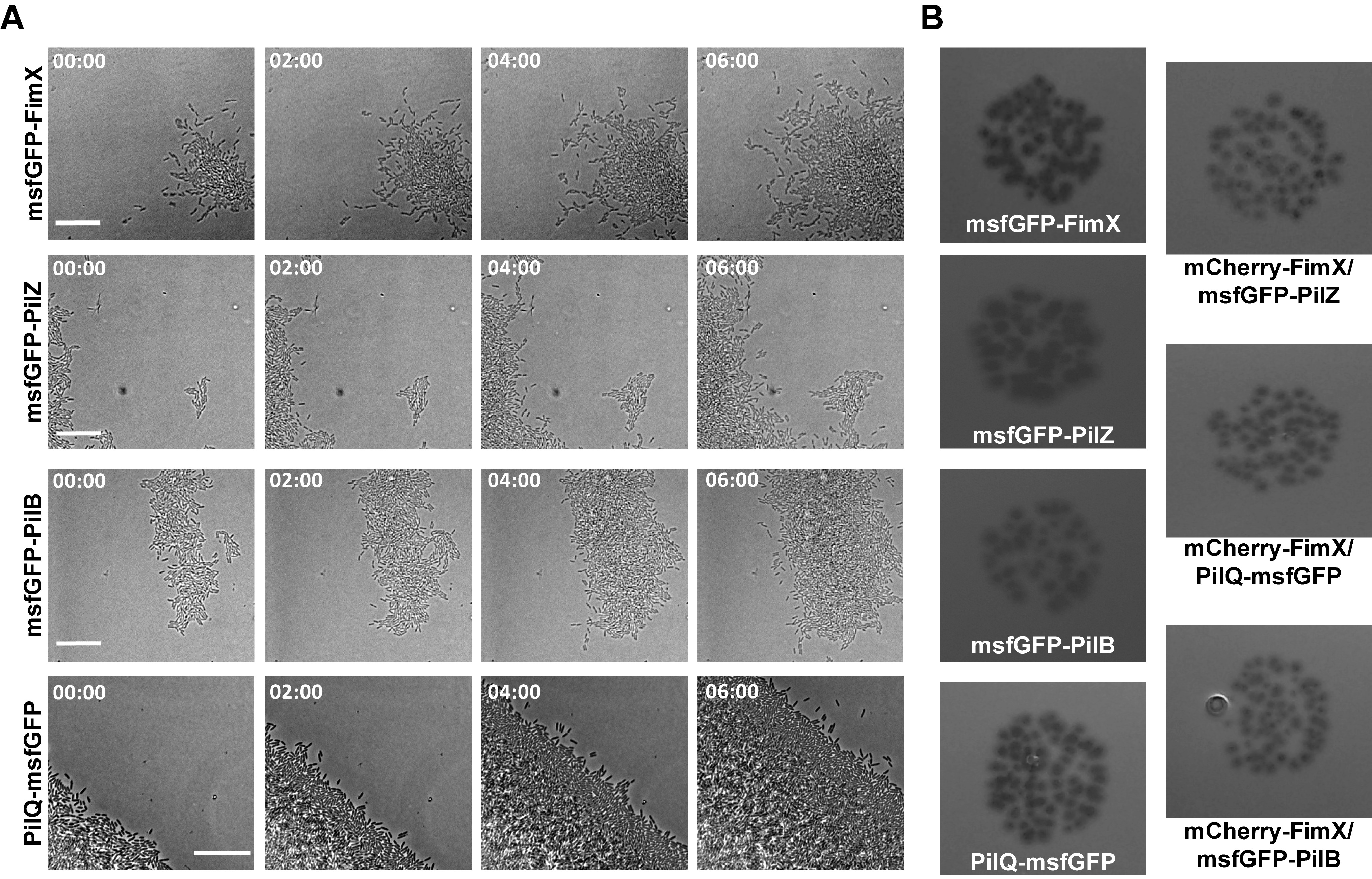

Supplement: S11 Fig — A) Time-lapse of in X. citri msfGFP-FimX, msfGFP-PilZ, msfGFP-PilB and PilQ-msfGFP strains exhibiting twitching motility. The time lapse interval (h) is indicated for each frame. Images were taken by using a phase-contrast microscope. Scale bar, 20 μm. Also see S1–S4 Movies. B) Phage ΦXacm4-11 infection assays for X. citri msfGFP-FimX, msfGFP-PilZ, msfGFP-PilB, PilQ-msfGFP, mCherry-FimX/msfGFP-PilZ, mCherry-FimX/PilQ-msfGFP and mCherry-FimX/msfGFP-PilB strains. Dark plaques are indicative of phage-induced bacterial lysis in a confluent culture background. (TIF) [file ppat.1009808.s011.tif]

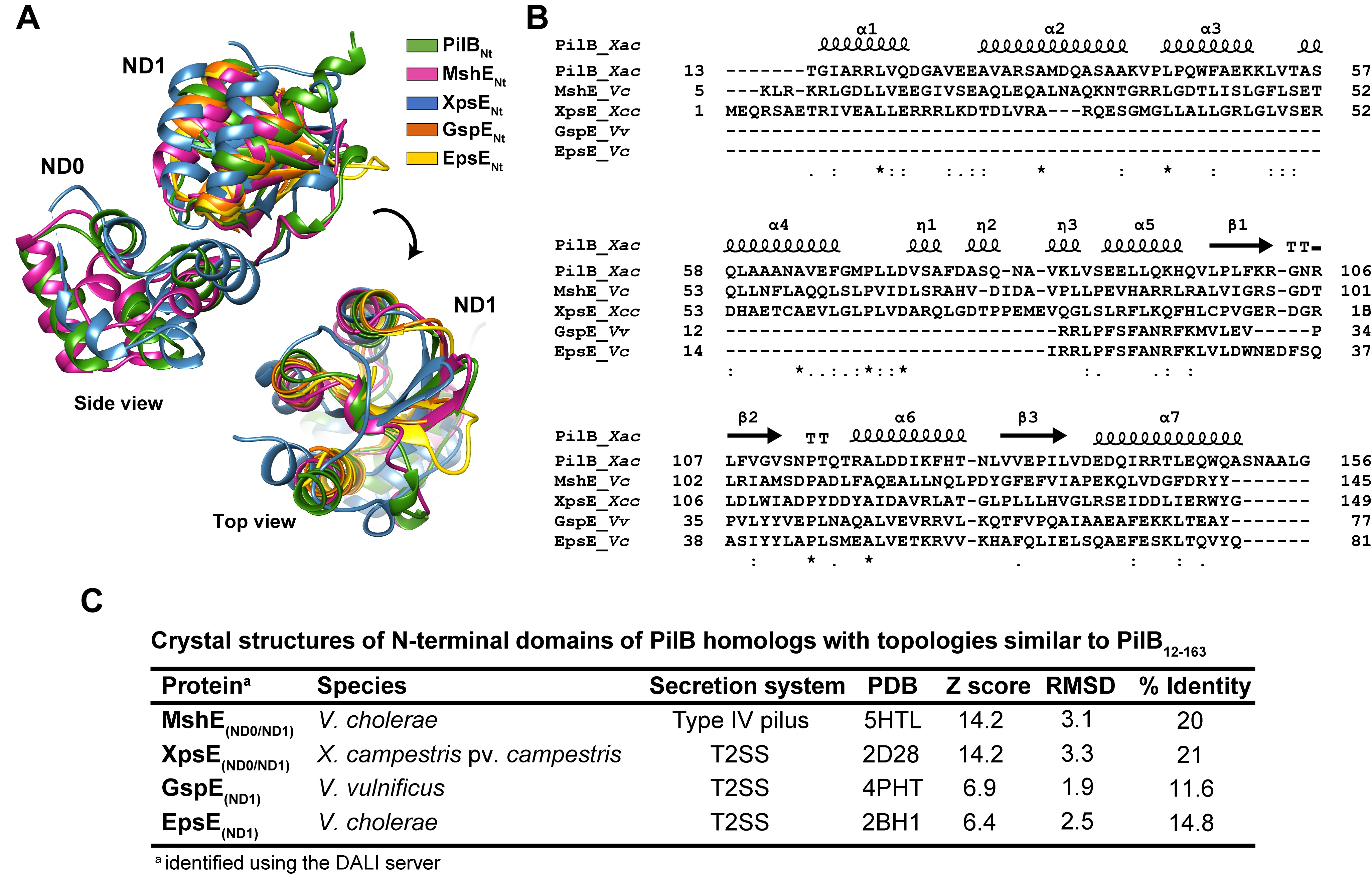

Supplement: S12 Fig — A) Structural and B) sequence alignment of the N-terminal regions of X. citri PilB (green) with homologs from the Mannose-Sensitive Haemagglutinin Type IV Pilus of V. cholerae (MshE, PDB: 5HTL, light red) and the Type II secretion systems of X. campestris (XpsE, PDB: 2D28, light blue), Vibrio vulnificus (GspE, PDB: 4PHT, orange) and Vibrio cholerae (EpsE, PDB: 2BH1, yellow). The structural alignment in A shows both side and top views. The secondary structure elements observed in the crystal structure of X. citri PilB12-163 are indicated above of the sequence alignment in B. C) Summary of the structural and sequence alignment statistics described in A and B. (TIF) [file ppat.1009808.s012.tif]

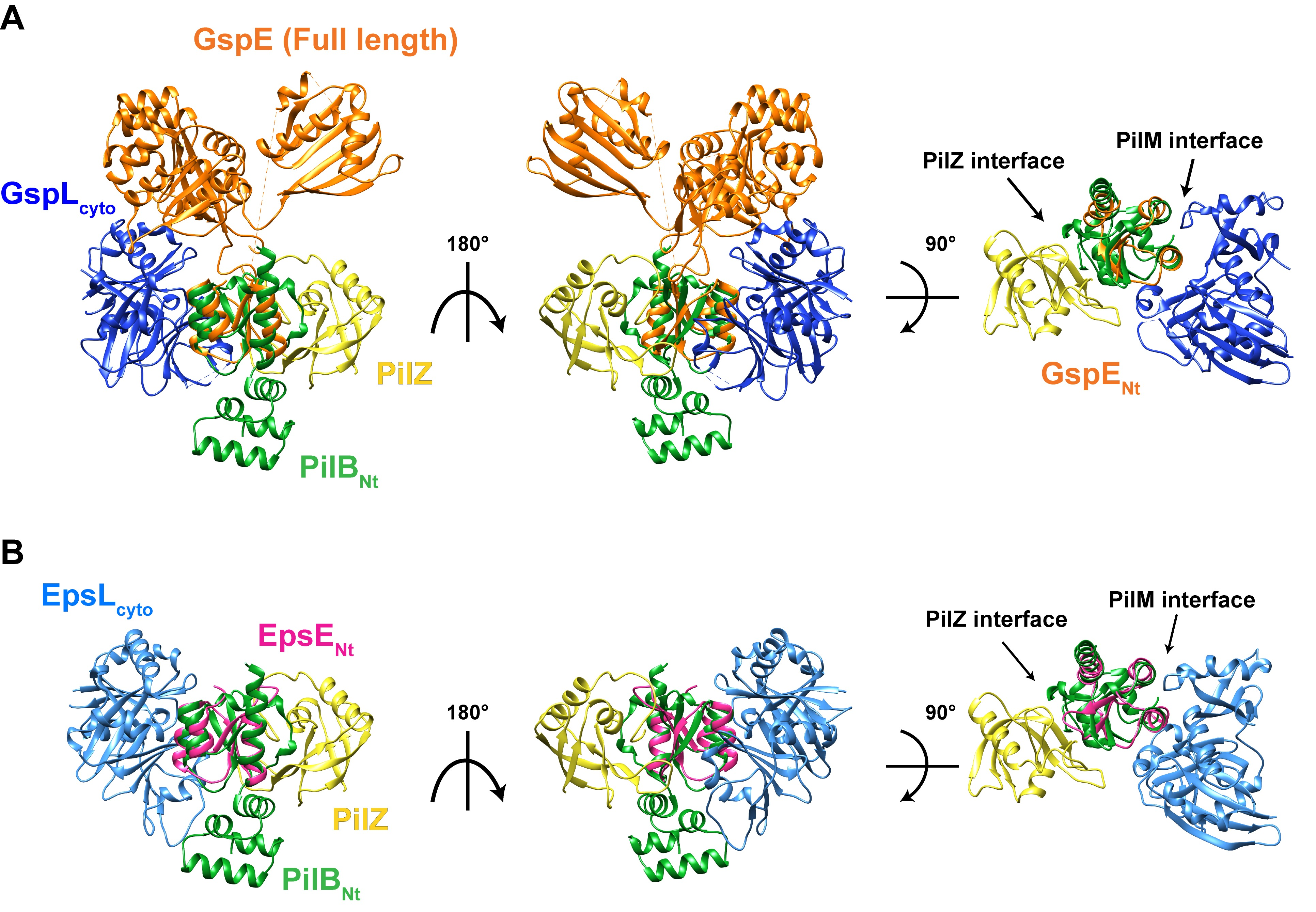

Supplement: S13 Fig — Superposition of the X. citri PilB12-163-PilZ complex (colored in green for PilB and yellow for PilZ) with (A) GspE-GspLcyto (PDB: 4PHT, colored in orange for GspE and blue for GspLcyto) and (B) EpsENt-EpsLcyto (PDB: 2BH1, colored in magenta for EpsENt and light blue for EpsLcyto). The ND1 sub-domain of X. citri PilB and de N-terminal domains of GspE and EpsE were used as reference in the alignments. (TIF) [file ppat.1009808.s013.tif]

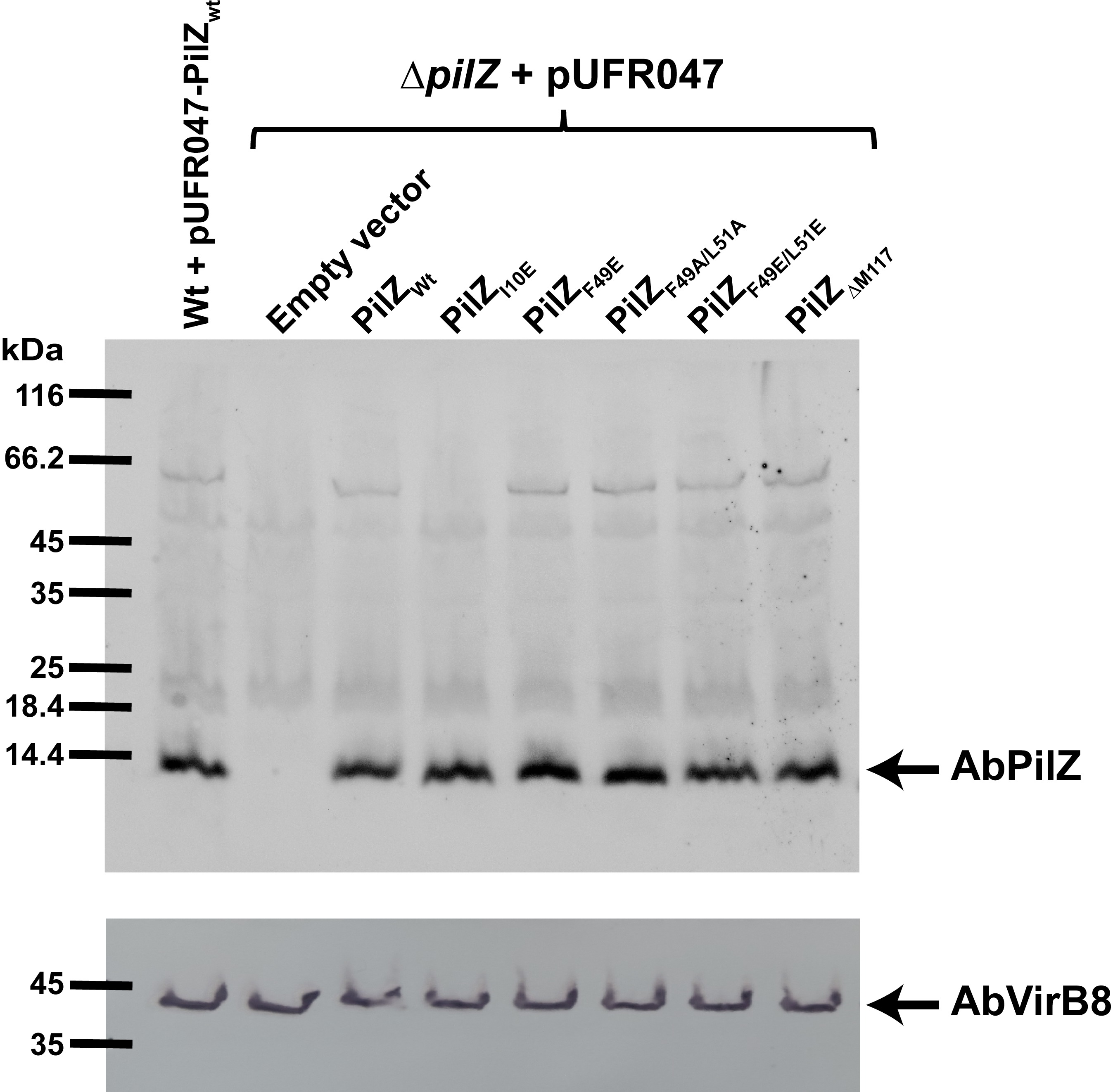

Supplement: S14 Fig — Western blot assays using polyclonal antibodies (Ab) against PilZ (above) and VirB8 (below). The first lane contains total extract from wild type X. citri strain containing the pUFR047-PilZWT vector. The following lanes contain total extracts from X. citri ΔpilZ cells carrying the empty pUFR047 vector and the vector directing the expression of PilZWt, PilZI10E, PilZF49E, PilZF49E/L51E, PilZF49A/L51A, PilZΔM117. The same amounts of total protein were loaded on the gel. Detection of the X. citri VirB8 protein (XAC2621) was used as a control. Experiments were repeated three times with similar results. (TIF) [file ppat.1009808.s014.tif]
