# Supplementary material for: The PilB-PilZ-FimX regulatory complex of the Type IV pilus from Xanthomonas citri
Source: PLoS Pathog. 2021 Aug 16;17(8):e1009808. doi: 10.1371/journal.ppat.1009808 (PMC8389850; doi:10.1371/journal.ppat.1009808)
Supplement: S1 Table — (DOCX) [file ppat.1009808.s015.docx]

**Supplementary Table 1**: Data collection and refinement statistics of the PilB_12-163_-PilZ and PilZ_Δ107-117_-FimX_GGDEF-EAL_-c-di-GMP crystal structures.

|  | **PilB_12-163_-PilZ complex** | | | **PilZ_Δ107-117_-FimX_GGDEF-EAL_ -c-di-GMP** |
| --- | --- | --- | --- | --- |
|  | SetMet Crystal (PilB_12-163__P70S) | Native Crystal  (PilB_12-163__P70S) | Native Crystal (Wild type) | Native Crystal |
| Data collection |  |  |  |  |
| Space group | P1 2_1_ 1 | P1 2_1_ 1 | P1 2_1_ 1 | P4_2_ 2_1_ 2 |
| Cell dimensions |  |  |  |  |
| a, b, c (Å) | 39.0, 122.3, 62.4 | 39.0, 122.4, 62.7 | 39.4, 123.5, 63.5 | 96.1, 96.1, 146.3 |
| α, β, γ (°) | 90.0, 98.6, 90.0 | 90.0, 99.0, 90.0 | 90.0, 99.3, 90.0 | 90.0, 90.0, 90.0 |
| Resolution (Å) | 30 – 1.7 | 28 – 2.0 | 44 – 2.9 | 50 – 3.4 |
| R_merge_ | 0.08 (0.79) | 0.15 (1.25) | 0.06 (0.19) | 0.07 (0.56) |
| I/sI | 8.8 (1.3) | 10.1 (1.7) | 9.6 | 11.0 (3.3) |
| Completeness (%) | 99.36 (99.16) | 99.6 (99.9) | 99.6 (97.6) | 100 (100.0) |
| No. reflections | 211040 | 226586 | 427980 | 139748 |
| Unique reflections | 62097 | 44979 | 13503 | 9997 |
| Redundancy | 1.8 (1.8) | 5.0 (4.8) | 2.1 (2.0) | 13.6 (14.0) |
| No. of images | 599 | 2789 | 397 | 360 |
| Oscillation per image | 0.3° | 0.1 ° | 0.5° | 1º |
| Wavelength (Å) | 0.97889 | 1.45866 | 1.5418 | 1.5418 |
| X-ray beamline | W01B-MX2 | W01B-MX2 | IQ-USP | IQ-USP |
|  |  |  |  |  |
| Refinement |  |  |  |  |
| Resolution | 26 – 1.71 | 28 – 2.0 | 44 – 2.88 | 50 – 3.4 |
| No. reflections | 61953 | 39155 | 13503 | 9962 |
| R_work_/R_free_ | 0.20/0.25 | 0.20/024 | 0.15/0.22 | 0.23/0.28 |
| No. residues or atoms |  |  |  |  |
| Protein | 513 | 507 | 512 | 348 |
| Water | 379 | 486 | 16 | --- |
| Ramachandran plot (%) |  |  |  |  |
| Most favored regions | 96.44 | 97.7 | 96,03 | 90.03 |
| Allowed regions | 3.17 | 2.3 | 3.77 | 7.33 |
| Disallowed regions | 0.40 | 0 | 0.20 | 1.2 |
| r.m.s. deviations |  |  |  |  |
| Bond lengths (Å) | 0,002 | 0.007 | 0.013 | 0.012 |
| Bond angles (°) | 2.40 | 0.96 | 1.46 | 1.45 |
| Deposited PDB code | 7LKN | 7LKM | 7LKO | 7LKQ |
