# Supplementary material for: The PilB-PilZ-FimX regulatory complex of the Type IV pilus from Xanthomonas citri
Source: PLoS Pathog. 2021 Aug 16;17(8):e1009808. doi: 10.1371/journal.ppat.1009808 (PMC8389850; doi:10.1371/journal.ppat.1009808)
Supplement: S2 Table — (DOCX) [file ppat.1009808.s016.docx]

**Supplementary Table 2**: Interface residues in the PilB_12-163_-PilZ complex.

Residues at the interface were identified using the PISA server (http://www.ebi.ac.uk).

| **PilB_12-163_-PilZ Complex** | | | |
| --- | --- | --- | --- |
| **PilZ residues (chain C)** | | **PilB_12-163_ residues (chain A)** | |
| **Residues** | **PilZ Motif*** | **Residues** | **PilB_N-terminal_**  **sub-domain** |
| Tyr22 | MI | Arg18 | ND0 |
| Met26 | MI | Asp22 | ND0 |
| Pro27 | MI | Ala61 | ND1 |
| Phe28 | MI | Val64 | ND1 |
| Val29 | MI | Ala65 | ND1 |
| Lys30 | MI | Gly68 | ND1 |
| Arg41 | - | Met69 | ND1 |
| Met43 | - | Pro70 | ND1 |
| Leu44 | - | Leu71 | ND1 |
| Lys66 | MII | Leu72 | ND1 |
| Ile68 | MII | Asp73 | ND1 |
| Trp69 | MII | Ser75 | ND1 |
| Thr70 | MII | Ala76 | ND1 |
| Thr71 | MII | Phe77 | ND1 |
| Pro72 | MII | Asp78 | ND1 |
| Ala75 | - | Gln81 | ND1 |
| Gln76 | - | Leu100 | ND1 |
| Arg79 | - | Phe101 | ND1 |
| Gln86 | - | Arg103 | ND1 |
| Thr108 | - | Gly104 | ND1 |
| Ser110 | MV | Phe108 | ND1 |
| Asp111 | MV | Glu132 | ND1 |
| Lys112 | MV | Pro133 | ND1 |
| Pro113 | MV | Ile134 | ND1 |
| Thr114 | MV |  |  |
| Thr116 | MV |  |  |
| Met117 | MV |  |  |

* See Guzzo et al. (2009)  [*J. Mol. Biol.* **393**, 848–866](http://paperpile.com/b/0CxpkD/gJMS6).
