# Supplementary material for: The PilB-PilZ-FimX regulatory complex of the Type IV pilus from Xanthomonas citri
Source: PLoS Pathog. 2021 Aug 16;17(8):e1009808. doi: 10.1371/journal.ppat.1009808 (PMC8389850; doi:10.1371/journal.ppat.1009808)
Supplement: S3 Table — (DOCX) [file ppat.1009808.s017.docx]

**Supplementary Table 3:** Selected Bacterial species in the KEGG database that code for homologs of *X. citri* PilZ, FimX, PilB and PilM.

| ***Organism*** | **PilZ** | **% Ident** | **FimX** | **% Ident** | **PilB** | **% Ident** | **% Ident (ND0/ND1)*** | **PIlM** | **% Ident** | **Order** | **Family** |
| --- | --- | --- | --- | --- | --- | --- | --- | --- | --- | --- | --- |
| *Xanthomona citri* pv*. citri* | XAC1133 |  | XAC2398 |  | XAC3239 |  |  | XAC3385 |  | Xanthomonadales | Xanthomonadaceae |
| *Stenotrophomonas maltophilia* | Smal_0879 | 88.9 | Smal_1620 | 69.4 | Smal_3167 | 86.26 | 78.5 | Smal_3240 | 89.4 | Xanthomonadales | Xanthomonadaceae |
| *Xylella fastidiosa* 9a5c | XF_0677 | 77.8 | XF_2624 | 61.2 | XF_2544 | 84.26 | 79.4 | XF_0369 | 81.7 | Xanthomonadales | Xanthomonadaceae |
| *Lysobacter sp.* TY2-98 | DWG18_08455 | 75.6 | DWG18_08230 | 55.7 | DWG18_00855 | 78.21 | 62.3 | DWG18_01755 | 78.4 | Xanthomonadales | Xanthomonadaceae |
| *Luteimonas sp.* 100111 | CNR27_10860 | 75.9 | CNR27_09350 | 53 | CNR27_05335 | 77.68 | 65.1 | CNR27_05520 | 84.4 | Xanthomonadales | Xanthomonadaceae |
| *Thermomonas sp.* HDW16 | G7079_09710 | 76.5 | G7079_09395 | 53.8 | G7079_12190 | 72.66 | 56.7 | G7079_03685 | 78.7 | Xanthomonadales | Xanthomonadaceae |
| *Dokdonella koreensis* | I596_2272 | 72.6 | I596_2151 | 42.3 | I596_2535 | 71.3 | 52.3 | I596_2114 | 75.6 | Xanthomonadales | Rhodanobacteraceae |
| *Aerosticca soli* | ALSL_1399 | 65.8 | ALSL_1755 | 41.7 | ALSL_2144 | 65.63 | 38.4 | ALSL_2080 | 75.3 | Xanthomonadales | Rhodanobacteraceae |
| *Dyella jiangningensis* | CH75_05365 | 63.5 | CH75_03455 | 40.9 | CH75_02365 | 66.15 | 46.6 | CH75_00160 | 73.8 | Xanthomonadales | Rhodanobacteraceae |
| *Luteibacter pinisoli* | FIV34_13465 | 63.1 | FIV34_14395 | 38.5 | FIV34_05995 | 68.13 | 45.4 | FIV34_02845 | 72.7 | Xanthomonadales | Rhodanobacteraceae |
| *Ahniella affigens* | C7S18_05390 | 66.6 | C7S18_13180 | 40.8 | C7S18_21390 | 66.9 | 49.3 | C7S18_16635 | 73.3 | Xanthomonadales | Rhodanobacteraceae |
| *Methylophaga nitratireducenticrescens* | Q7A_2006 | 55.2 | Q7A_611 | 30.1 | Q7A_646 | 55.6 | 36.2 | Q7A_63 | 51.1 | Thiotrichales | Piscirickettsiaceae |
| *Thioploca ingrica* | THII_2180 | 49.5 | THII_1913 | 30.6 | THII_1978 | 54.8 | 40.4 | THII_3928 | 48.3 | Thiotrichales | Thiotrichaceae |
| *Azotobacter chroococcum* | Achr_27870 | 65.5 | Achr_33770 | 31.7 | Achr_30470 | 54.02 | 38 | Achr_6600 | 58.9 | Pseudomonadales | Pseudomonadaceae |
| *Pseudomonas aeruginosa* PAO1 | PA2960 | 64.9 | PA4959 | 31.5 | PA4526 | 53.43 | 36.3 | PA5044 | 61.7 | Pseudomonadales | Pseudomonadaceae |
| *Permianibacter aggregans* | E2H98_18075 | 66.6 | E2H98_13370 | 30.6 | E2H98_04800 | 58.7 | 37.1 | E2H98_12465 | 59.8 | Pseudomonadales | Pseudomonadaceae |
| *Moraxellaceae bacterium* | HYN46_08050 | 48.6 | HYN46_06030 | 26.6 | HYN46_16760 | 55.3 | 37.4 | HYN46_03015 | 56.2 | Pseudomonadales | Moraxellaceae |
| *Hahella sp.* KA22 | ENC22_10985 | 67 | ENC22_06125 | 32.3 | ENC22_06680 | 57.96 | 46 | ENC22_25710 | 60.8 | Oceanospirillales | Hahellaceae |
| *Oleiphilus messinensis* | OLMES_3292 | 65.8 | OLMES_1812 | 30.4 | OLMES_1928 | 55.56 | 42.6 | OLMES_0785 | 59.4 | Oceanospirillales | Oleiphilaceae |
| *Ketobacter alkanivorans* | Kalk_20345 | 62.4 | Kalk_14845 | 32.5 | Kalk_17505 | 56.51 | 40.1 | Kalk_15635 | 62 | Oceanospirillales | Alcanivoracaceae |
| *Bermanella marisrubri:* | HF888_07565 | 66.1 | HF888_14900 | 31.1 | HF888_13000 | 55.17 | 39.2 | HF888_15305 | 53.8 | Oceanospirillales | Oceanospirillaceae |
| *Oleispira antarctica* | OLEAN_C19950 | 64.9 | OLEAN_C01830 | 30.2 | OLEAN_C29920 | 53.77 | 41 | OLEAN_C36400 | 56.9 | Oceanospirillales | Oceanospirillaceae |
| *Bacterioplanes sanyensis* | CHH28_14850 | 62.2 | CHH28_09375 | 31.6 | CHH28_09770 | 55.8 | 39.3 | CHH28_01695 | 59.2 | Oceanospirillales | Oceanospirillaceae |
| *Thalassolituus oleivorans* | R615_09275 | 59.1 | R615_02380 | 30.6 | R615_13960 | 57.6 | 43.9 | R615_15265 | 59 | Oceanospirillales | Oceanospirillaceae |
| *Neptunomonas concharum* | F0U83_10125 | 55.5 | F0U83_14570 | 24.7 | F0U83_04590 | 54.3 | 32.4 | F0U83_01745 | 40.6 | Oceanospirillales | Oceanospirillaceae |
| *Marinobacterium aestuarii* | A8C75_09375 | 51.8 | A8C75_19145 | 24.8 | A8C75_05340 | 54.5 | 43.8 | A8C75_01950 | 37.7 | Oceanospirillales | Oceanospirillaceae |
| *Gynuella sunshinyi* | YC6258_03291 | 60.7 | YC6258_02356 | 30 | YC6258_02406 | 56 | 43.1 | YC6258_01046 | 57.4 | Oceanospirillales | Saccharospirillaceae |
| *Reinekea forsetii* | REIFOR_01634 | 63.9 | REIFOR_00871 | 26.1 | REIFOR_02657 | 56.2 | 45.5 | REIFOR_00489 | 56.5 | Oceanospirillales | Saccharospirillaceae |
| *Solimonas sp.* K1W22B-7 | D0B54_12355 | 64.3 | D0B54_10465 | 24.1 | D0B54_18910 | 56.9 | 40.6 | D0B54_00665 | 48 | Nevskiales | Sinobacteraceae |
| *Thioalkalivibrio sulfidiphilus* | Tgr7_1827 | 67.5 | Tgr7_1229 | 31.1 | Tgr7_0787 | 60.94 | 44.6 | Tgr7_3023 | 59.5 | Chromatiales | Ectothiorhodospiraceae |
| *Acidihalobacter prosperus* | BI364_09100 | 60.5 | BI364_06205 | 30 | BI364_14440 | 55.87 | 37.5 | BI364_00955 | 58.1 | Chromatiales | Ectothiorhodospiraceae |
| *Nitrosococcus halophilus:* | Nhal_3124 | 65.8 | Nhal_0222 | 36.6 | Nhal_3847 | 59.86 | 43.4 | Nhal_3891 | 59 | Chromatiales | Chromatiaceae |
| *Alkalilimnicola ehrlichii* | Mlg_1414 | 63.4 | Mlg_0682 | 32.3 | Mlg_2079 | 60.17 | 46.9 | Mlg_2755 | 55.4 | Chromatiales | Ectothiorhodospiraceae |
| *Ectothiorhodospira sp.* BSL-9 | ECTOBSL9_2685 | 64.9 | ECTOBSL9_2055 | 33 | ECTOBSL9_0390 | 58.99 | 41.6 | ECTOBSL9_0545 | 58.9 | Chromatiales | Ectothiorhodospiraceae |
| *Marichromatium purpuratum* | MARPU_07710 | 55.6 | MARPU_11090 | 24.9 | MARPU_14625 | 56.67 | 39.1 | MARPU_16500 | 57.2 | Chromatiales | Chromatiaceae |
| *Wenzhouxiangella marina* | WM2015_1579 | 51.8 | WM2015_1708 | 30 | WM2015_1886 | 57.4 | 33.8 | WM2015_2487 | 49.7 | Chromatiales | Wenzhouxiangellaceae |
| *Zhongshania aliphaticivorans* | AZF00_09490 | 64.3 | AZF00_03550 | 23.9 | AZF00_14490 | 57.56 | 38.5 | AZF00_04275 | 57.6 | Cellvibrionales | Spongiibacteraceae |
| *Teredinibacter turnerae:* | TERTU_1728 | 65.2 | TERTU_0664 | 27.3 | TERTU_3034 | 58.49 | 39 | TERTU_3585 | 58.9 | Cellvibrionales | Cellvibrionaceae |
| *Saccharophagus degradans* | Sde_1653 | 65.2 | Sde_1079 | 27.7 | Sde_0863 | 56.77 | 43 | Sde_2691 | 58.5 | Cellvibrionales | Cellvibrionaceae |
| *Simiduia agarivorans:* | M5M_03615 | 62.7 | M5M_10020 | 29.4 | M5M_17885 | 58.81 | 42.4 | M5M_14725 | 61.6 | Cellvibrionales | Cellvibrionaceae |
| *Microbulbifer sp.* THAF38 | FIU95_09070 | 63 | FIU95_01555 | 27.6 | FIU95_15490 | 56.49 | 37.6 | FIU95_01055 | 57.6 | Cellvibrionales | Microbulbiferaceae |
| *Oceanicoccus sagamiensis* | BST96_16360 | 63.4 | BST96_05150 | 25.6 | BST96_02255 | 57.6 | 40 | BST96_08775 | 59.4 | Cellvibrionales | Spongiibacteraceae |
| *Cellvibrio sp.* PSBB006 | CBR65_02310 | 68.7 | CBR65_09925 | 27.7 | CBR65_08180 | 55.7 | 42.3 | CBR65_16290 | 61.5 | Cellvibrionales | Cellvibrionaceae |
| *Microbulbifer sp.* A4B17 | BTJ40_13320 | 63 | BTJ40_01855 | 27.2 | BTJ40_16835 | 56.6 | 35.9 | BTJ40_01310 | 55.7 | Cellvibrionales | Microbulbiferaceae |
| *Marinobacter hydrocarbonoclasticus* VT8 | Maqu_1573 | 66.1 | Maqu_2785 | 31.7 | Maqu_2681 | 57.02 | 43.6 | Maqu_0826 | 59.1 | Alteromonadales | Alteromonadaceae |
| *Hydrocarboniclastica marina* | soil367_09915 | 62.3 | soil367_12215 | 31.9 | soil367_11680 | 56.79 | 44.4 | soil367_03455 | 58.5 | Alteromonadales | Alteromonadaceae |
| *Sulfurifustis variabilis* | SVA_1870 | 66.1 | SVA_2896 | 27.1 | SVA_0710 | 56.69 | 43.9 | SVA_0049 | 53.8 | Acidiferrobacterales | Acidiferrobacteraceae |
| *Sulfuricaulis limicola* | SCL_1378 | 66.1 | SCL_0794 | 27.4 | SCL_0565 | 57.44 | 40.4 | SCL_0058 | 54.3 | Acidiferrobacterales | Acidiferrobacteraceae |
| *Thiohalobacter thiocyanaticus* | FOKN1_1849 | 68.5 | FOKN1_1215 | 34.9 | FOKN1_2648 | 59.41 | 46.7 | FOKN1_0068 | 61.5 | Chromatiales |  |
| *Thiolapillus brandeum* | TBH_C1440 | 62.5 | TBH_C0650 | 26.4 | TBH_C0576 | 56 | 39.5 | TBH_C0162 | 57 | unclassified | unclassified |
| *Natrialbaceae archaeon* XQ-INN 246 ** | HC341_09930 | 64.9 | HC341_04835 | 33.1 | HC341_14290 | 62.22 | 48.9 | HC341_01050 | 57.5 | Natrialbales | Natrialbaceae |

* The alignment was based on residues 1-190 of *X. citri* PilB that includes the acidic linker region (residues 158-190) between ND1 and ND2.

**All species in this table belong to the Class Gammaproteobacteria with the exception of this archeal species belonging to the Class Halobacteria.
