# Supplementary material for: The PilB-PilZ-FimX regulatory complex of the Type IV pilus from Xanthomonas citri
Source: PLoS Pathog. 2021 Aug 16;17(8):e1009808. doi: 10.1371/journal.ppat.1009808 (PMC8389850; doi:10.1371/journal.ppat.1009808)
Supplement: S4 Table — (DOCX) [file ppat.1009808.s018.docx]

**Supplementary Table 4**. Cellular strains

| Strain | Description/purpose | Reference |
| --- | --- | --- |
| *Escherichia coli* |  |  |
| DH5α | For cloning | Invitrogen |
| BL21 (DE3) | For protein expression | Novagen |
| Cy BL21 (DE3) pLys | Incorporation of the 5OH-Thrytophan | Wong and Eftink, 1997^a^ |
| XL10-Gold | For introducing point mutations | Stratagene |
|  |  |  |
| *Saccharomyces cerevisiae* |  |  |
| PJ694-a | MATa trp1-901 leu2-3,112 ura3-52 his3-200 gal4Δ gal80Δ LYS2::GAL1-HIS3 GAL2-ADE2 met2::GAL7-lacZ | James, 1996^b^ |
|  |  |  |
| *Xanthomonas citri* pv. citri |  |  |
| *X. citri*_Wt | *Xanthomonas axonopodis* pv. citri str. 306 | De Silva et al., 2002^c^ |
| *X. citri*_ *ΔpilZ* | *X. citri* with deletion of the *pilZ_XAC1133_* | Guzzo et al., 2009^d^ |
| *X. citri_msfGFP-FimX* | *X. citri* with translational fusion de *msfgfp* at N-terminal of *fimX_XAC2398_* | This study |
| *X. citri_msfGFP-FimX_ΔpilZ* | *X. citri_msfGFP-FimX* with *pilZ_XAC1133_* deletion | This study |
| *X. citri_msfGFP-FimX_ΔpilB* | *X. citri_msfGFP-FimX* with *pilB_XAC3239_* deletion | This study |
| *X. citri_msfGFP-PilB* | *X. citri* with translational fusion de *msfgfp* at N-terminal of *pilB_XAC3239_* | This study |
| *X. citri_msfGFP-PilB_ΔpilZ* | *X. citri_msfGFP-PilB* with *pilZ_XAC1133_* deletion | This study |
| *X. citri_msfGFP-PilB_ΔfimX* | *X. citri_msfGFP-PilB* with *fimX_XAC_*_2398_ deletion | This study |
| *X. citri_msfGFP-PilB/mCherry-FimX* | *X. citri* with translational fusion de *msfgfp* at N-terminal of *pilB_XAC3239_ and mcherry at N-terminal of fimX_XAC2398_* | This study |
| *X. citri_msfGFP-PilZ* | *X. citri* with translational fusion de *msfgfp* at N-terminal of *pilZ_XAC1133_* | This study |
| *X. citri_msfGFP-PilZ_ΔpilB* | *X. citri_msfGFP-PilZ* with *pilB_XAC3239_* deletion | This study |
| *X. citri_msfGFP-PilZ_ΔfimX* | *X. citri_msfGFP-PilZ* with *fimX_XAC2398_* deletion | This study |
| *X. citri_msfGFP-PilZ / mCherry-FimX* | *X. citri* with translational fusion de *msfgfp* at N-terminal of *pilZ_XAC1133_ and mcherry at N-terminal of fimX_XAC2398_* | This study |
| *X. citri_PilQ-msfGFP* | *X. citri* with translational fusion de *msfgfp* at C-terminal of *pilQ_XAC3381_* | This study |
| *X. citri_PilQ-msfGFP_ΔpilZ* | *X. citri_PilQ-msfGFP* with *pilZ_XAC1133_* deletion | This study |
| *X. citri_PilQ-msfGFP_ΔpilB* | *X. citri_PilQ-msfGFP* with *pilB_XAC3239_* deletion | This study |
| *X. citri_PilQ-msfGFP_ΔfimX* | *X. citri_PilQ-msfGFP* with *fimX_XAC2398_* deletion | This study |
| *X. citri_PilQ-msfGFP / mCherry-FimX* | *X. citri* with translational fusion de *msfgfp* at N-terminal of *pilQ_XAC3381_ and mcherry at N-terminal of fimX_XAC2398_* | This study |

^a^ Wong and Eftink, (1997) *Protein Sci.* **6**, 689-697

^b^ da Silva et al. (2002) *Nature* **417**, 459-463

^c^ James et al. (1996) Genetics 144, 1425-1436

^d^ Guzzo et al. (2009)  [*J. Mol. Biol.* **393**, 848–866](http://paperpile.com/b/0CxpkD/gJMS6).
