# Supplementary material for: The PilB-PilZ-FimX regulatory complex of the Type IV pilus from Xanthomonas citri
Source: PLoS Pathog. 2021 Aug 16;17(8):e1009808. doi: 10.1371/journal.ppat.1009808 (PMC8389850; doi:10.1371/journal.ppat.1009808)
Supplement: S5 Table — (DOCX) [file ppat.1009808.s019.docx]

**Supplementary Table 5.** Oligonucleotides and plasmids used in this study

| **Target** | **Primer name** | **Sequence 5’-3’** | |
| --- | --- | --- | --- |
| **Cloning of PilB constructions** |  |  | |
|  | **PilB_1_F** | AAAA **CCATGG** TA **CATATG**AATAGCGTCGTGACCG | |
|  | **PilB_12_F** | AAAA **CATATG** ATAACGGGCATCGCGCGTCGTC | |
|  | **PilB_159_F** | AAAA **CCATGG** TA **CATATG**CTTGGTGACGACGAGGAG | |
|  | **PilB_163_R** | TTTT **AAGCTT** TTATTCGTCGTCACCGAGCGCG | |
|  | **PilB_190_R** | TTTT **AAGCTT CTCGAG** TTAGCCCTTGGCATCAACCC | |
|  | **PilB_578_R** | TTTT **AAGCTT CTCGAG** TTATTCGTCGTCACCGAGCGCG | |
| **Cloning of FimX constructions** |  |  | |
|  | **FimX_1_F** | TTAT **CATATG** CAAAAAGGCAAAGATCTCACCC | |
|  | **FimX_153_F** | TTAT **CATATG** TCTCGTGACCCGATCGCCTAC | |
|  | **FimX_255_F** | AAAA **CATATG** GAGGAGTTCGACCCGGAAC | |
|  | **FimX_260_F** | AAAA **CATATG** GAACTGGCGCGCGAGGTCGAAGAC | |
|  | **FimX_689_R** | TT **GGATCC** TCAGCCGAAGTCGTAATTCATCAAC | |
|  |  |  | |
| **Cloning of PilZ constructions** |  |  | |
|  | **PilZ_1_F** | CTGA **CATATG** AGTGCAATGAATG | |
|  | **PilZ_ΔM117_R** | CAGT **GGATCC** CTCGAGTTACGTATGCGTCGGCTTG | |
|  | **PilZ_M117G_R** | CAGT **GGATCC** CTCGAGTTAGCCCGTATGCGTCG | |
|  |  |  | |
| **Insertion of point mutations** |  |  | |
|  | **PilB_F77A_F** | GTTGGACGTGTCGGCGGCCGACGCCAGCCAAAAC | |
|  | **PilB_F77A_R** | GTTTTGGCTGGCGTCGGCCGCCGACACGTCCAAC | |
|  | **PilB_F101A_F** | CAAGTGCTACCGCTGGCCAAGCGCGGCAACCG | |
|  | **PilB_F101A_F** | CGGTTGCCGCGCTTGGCCAGCGGTAGCACTTG | |
|  | **PilB_R103A_F** | CCGCTGTTCAAGGCCGGCAACCGGCTG | |
|  | **PilB_R103A_R** | CAGCCGGTTGCCGGCCTTGAACAGCGG | |
|  | **PilB_F108A_F** | GGCAACCGGCTGGCCGTAGGGGTGAG | |
|  | **PilB_F108A_R** | CTCACCCCTACGGCCAGCCGGTTGCC | |
|  | **PilB_E132A_F** | GAACTTGGTAGTCGCGCCCATCCTTGTGG | |
|  | **PilB_E132A_R** | CCACAAGGATGGGCGCGACTACCAAGTTC | |
|  | **PilB_K343A_F** | GACCGGCTCGGGCGCGACGGTGTCGTTG | |
|  | **PilB_K343A_R** | CAACGACACCGTCGCGCCCGAGCCGGTC | |
|  | **PilB_E407A_F** | CATCATGGTCGGCGCAATCCGTGACCTGG | |
|  | **PilB_E407A_R** | CCAGGTCACGGATTGCGCCGACCATGATG | |
|  | **PilZ_I10E_F** | AATGAATGCACGCCAAGGCGAGTTGTCGCTGGCGTTGAAAG | |
|  | **PilZ_I10E_R** | CTTTCAACGCCAGCGACAACTCGCCTTGGCGTGCATTCATT | |
|  | **PilZ_F49E/L51E_F** | ATGTTGGGCGATGAAGTCGAGCTGGAGCTGACCCTGCCGGACTC | |
|  | **PilZ_F49E/L51E_R** | GAGTCCGGCAGGGTCAGCTCCAGCTCGACTTCATCGCCCAACAT | |
|  | **PilZ_F49A/L51A_F** | GTTGGGCGATGAAGTCGCCCTGGCGCTGACCCTGCCGGAC | |
|  | **PilZ_ F49A/L51A_R** | GTCCGGCAGGGTCAGCGCCAGGGCGACTTCATCGCCCAAC | |
|  | **PilZ_D46A/E47A_F** | CGCTACATGTTGGGCGCTGCAGTCTTCCTGCTGCTG | |
|  | **PilZ_D46A/E47A_R** | CAGCAGCAGGAAGACTGCAGCGCCCAACATGTAGCG | |
|  |  |  | |
| **Construct in pNPTS138 for** **genomic insertion of *msfgfp* or *mcherry*** |  |  | |
| ***msfgfp*** |  |  | |
|  | **CW30_F** | GAACTGTTCACCGGTGTTG | |
|  | **CW168_R** | GCTGCCACCGCCACC TTTGTAGAGTTCATCCATGC | |
| ***liker*-*msfgfp*** |  |  | |
|  | **CW128_F** | AGCGGTGGCGGTGGCAGTAA | |
|  | **CW31_R** | TTTGTAGAGTTCATCCATGC | |
| ***mcherry*** |  |  | |
|  | **EL146_F** | GTGAGCAAGGGCGAGGAG | |
|  | **EL147_R** | CTTGTACAGCTCGTCCATG | |
| ***msfgfp-fimX*** |  |  | |
|  | **5׳CW164_F** | TCACTTAAGGCCTTGACTAGAGGGTC GACAGATCCGCACCCAGTGC | |
|  | **5׳CW165_R** | ACCGGTGAACAGTTCTTCACCTTTACT CATGCGCGGCTCCCTAG | |
|  | **3׳CW166_F** | ATGAACTCTACAAAGGTGGCGGTGGCAGC CAAAAAGGCAAAGATCTCACC | |
|  | **3׳CW167_R** | CAGGATATCTGGATCCACGAATTCGCTAG GAAACTGTGTTCGCCGAAG | |
|  | **EL13_F** | CAGTTGTTCTGCGACATCTCC | |
|  | **EL14_R** | CGTCCCCACGCTGAACAC | |
| ***mcherry-fimX*** |  |  | |
|  | **5׳EL148_R** | CTCGCCCTTGCTCACCATGCGCGGCTCCCTAG | |
|  | **3׳EL149_F** | CGAGCTGTACAAG GGTGGCGGTGGCAGC CAAAAAGGCAAAGATCTCACC | |
| ***msfgfp-PilB*** |  |  | |
|  | **5׳EL2_R** | ACCGGTGAACAGTTCTTCACCTTTACT CATCATTGTTCCCAATTGGC | |
|  | **3׳EL3_F** | ATGAACTCTACAAAGGTGGCGGTGGCAGC AATAGCGTCGTGACCGC | |
|  | **3׳EL4_R** | CAGGATATCTGGATCCACGAATTCGCTAG CACCAGCACCATGCCATAG | |
|  | **EL5_F** | CATGAAGAAGCAACAGGGTTTC | |
|  | **EL6_R** | CAGGCAAGCGGATTTCGAC | |
| ***msfgfp-pilZ*** |  |  | |
|  | **5׳EL7_F** | TCACTTAAGGCCTTGACTAGAGGGTC GAGAGCCTATGCCATGACCGC | |
|  | **5׳EL8_R** | ACCGGTGAACAGTTCTTCACCTTTACT CATCGAGTTCCCCTAGAACGTG | |
|  | **3׳EL9_F** | ATGAACTCTACAAAGGTGGCGGTGGCAGC AGTGCAATGAATGCACGCC | |
|  | **3׳EL10_R** | CAGGATATCTGGATCCACGAATTCGCTAG CCATCGCAACCAGAGCAC | |
|  | **EL11_F** | CTGTATTTCGTGGCGGTGG | |
|  | **EL12_R** | CTTTTCCGACTTCACATCGAC | |
|  |  |  | |
|  |  |  | |
| ***pilQ*-*msfgfp*** |  |  | |
|  | **5׳EL140_F** | TCACTTAAGGCCTTGACTAGAGGGTC GAGATCAACTACCACAACGCTGC | |
|  | **5׳EL141_R** | GCCACCGCCACCGCT TTTAGTAGCACCAGCAACACG | |
|  | **3׳EL142_F** | GGATGAACTCTACAAA TAAATAGTCCTGAATTTTTGATGGG | |
|  | **3׳EL143_R** | CAGGATATCTGGATCCACGAATTCGCTAG CAGCGAGGTGAATTGACGGTG | |
|  | **EL144_F** | CTGGACAAGCGACGCGATGG | |
|  | **EL145_R** | CACGGATGGCGGTGGTCTTG | |
|  |  |  | |
| **Construct in pNPTS138 for gene deletion** |  |  | |
| *pilB_XAC3239_* deletion |  |  | |
|  | **5׳EL31_F** | TCACTTAAGGCCTTGACTAGAGGGTCGA CGCTGCCCCAGTATCAGAAC | |
|  | **5׳EL32_R** | AACTGCTTCTTCGACCGC | |
|  | **3׳EL33_F** | GCGGTCGAAGAAGCAGTT AATGCGATGCAGATCGCC | |
|  | **3׳EL34_R** | CAGGATATCTGGATCCACGAATTCGCTAGGCCCTTCGTCCTTGGTTAC | |
|  | **EL74** | CAGTTGTCCAGACAAAACAG | |
| *fimX_XAC2398_* deletion |  |  | |
|  | **5׳EL91_F** | TCACTTAAGGCCTTGACTAGAGGGTCGA CTTGGCCAAGCAGTTTGG | |
|  | **5׳EL92_R** | CAGTTCTTCCTGGCTTTGC | |
|  | **3׳EL93_F** | GCAAAGCCAGGAAGAACTG ATTCTGACAATGGCCGAATTC | |
|  | **3׳EL94_R** | CAGGATATCTGGATCCACGAATTCGCTAG GTCGTTGCTTGGCGTCAATC | |
|  | **EL95_F** | GATGTGCTGTCGGTCCAGC | |
|  | **EL96_R** | CTGAATCGTCGGTCAGGTG | |
| *pilZ_XAC1133_* deletion |  |  | |
|  | **5׳EL174_F** | CAACGCCAGCGACAAAATGC | |
|  | **5׳EL175_R** | GCATTTTGTCGCTGGCGTTG GGATTGACCACCTCGGACAAG | |
|  | **3׳EL176_F** | CAGGATATCTGGATCCACGAATTCGCTAG CAATCCAAACGACATGAGGC | |
|  | **3׳EL177_R** | CATGCATGCATGACTTCCAC | |
|  |  |  | |
| **Plasmid** | **Description / Purpose** | | **Reference*** |
| pET28a | For expression of N-terminally 6xHis-tagged proteins with a thrombin site. Kn^R^ | | Novagen |
| pET3a | For protein expression. Amp^R^ | | Novagen |
| pETDuet-1 | For protein co-expression. Amp^R^ | | Novagen |
| pURF047 | For complementation of *X. citri* knockout strains*.* Gm^R^ | | Guzzo et al, 2009^a^ |
| pNPTS138 | Suicide vector for specific genomic insertion or deletion in *X. citri*. KnR | | Guzzo et al, 2013^b^ |
| pBBR1-MCS2-GFP | Constitutive GFP expression in *X. citri* strains. Kn^R^ | | Dunger et al., 2014^c^ |
| pilZ_pOAD | For Two-hybrid assay. Prey vector | | Guzzo et al, 2009^a^ |
| pilZ_pURF047 | For complementation of *X. citri* Δ*pilZ_XAC1133_.* Gm^R^ | | Guzzo et al, 2009 ^a^ |
| PilZ_pET3a | For heterologous expression | | Guzzo et al, 2009 ^a^ |
| PilZ_Δ107-117__pET3a |  |  | Guzzo et al, 2009 ^a^ |
| FimX_EAL__pET3a |  |  | Guzzo et al, 2013 ^b^ |
