# Supplementary material for: The PilB-PilZ-FimX regulatory complex of the Type IV pilus from Xanthomonas citri
Source: PLoS Pathog. 2021 Aug 16;17(8):e1009808. doi: 10.1371/journal.ppat.1009808 (PMC8389850; doi:10.1371/journal.ppat.1009808)
Supplement: S6 Table — (DOCX) [file ppat.1009808.s020.docx]

**Supplementary Table 6.** Conditions for protein expression

| **Protein** | **Vector** | **Strain** | **Conditions** |
| --- | --- | --- | --- |
| FimX_GGDEF-EAL_ | pET28a | E. coli BL21 (DE3) | 22 °C / 6 h |
| FimX_PAS-GGDEF-EAL_ | pET28a | E. coli BL21 (DE3) | 22 °C / 6 h |
| FimX_GGDEF-EAL_ - PilZ_Δ107-117_ complex | pET28a / pET3a | E. coli BL21 (DE3) STAR | 22 °C / 4 h |
| PilB_12-163_ | pET28a | E. coli BL21 (DE3) RIL | 22 °C / 6 h |
| PilB_1-190_ and mutants | pET28a | E. coli BL21 (DE3) RIL | 18°C / overnight |
| PilB_12-163_-PilZ complex | pET28a / pET3a | E. coli BL21 (DE3) RIL | 18°C / overnight |
| PilB_1-190_-PilZ complex | pET28a / pET3a | E. coli BL21 (DE3) RIL | 18°C / overnight |
| PilB-PilZ | pETDuet | E. coli BL21 (DE3) | 18°C / overnight |
| PilZ_5OH_W69_ | pET3a | Cy BL21 (DE3) pLys | 37°C / 4 h |
